# PAMAM-calix-dendrimers: Synthesis and Thiacalixarene Conformation Effect on DNA Binding

**DOI:** 10.3390/ijms222111901

**Published:** 2021-11-02

**Authors:** Olga Mostovaya, Pavel Padnya, Igor Shiabiev, Timur Mukhametzyanov, Ivan Stoikov

**Affiliations:** A.M. Butlerov’ Chemistry Institute, Kazan Federal University, 420008 Kazan, Russia; olga.mostovaya@mail.ru (O.M.); shiabiev.ig@yandex.ru (I.S.); timmie.m@gmail.com (T.M.)

**Keywords:** thiacalixarene, DNA, dendrimer, PAMAM, DNA binding, DNA storage

## Abstract

A convenient method for the synthesis of the first generation PAMAM dendrimers based on the thiacalix[4]arene has been developed for the first time. Three new PAMAM-calix-dendrimers with the macrocyclic core in *cone*, *partial cone*, and *1,3-alternate* conformations were obtained with high yields. The interaction of the obtained compounds with salmon sperm DNA resulted in the formation of the associates of the size up to 200 nm, as shown by the UV-Vis spectroscopy, DLS, and TEM. It was demonstrated by the CD method that the structure of the DNA did not undergo significant changes upon binding. The PAMAM-calix-dendrimer based on the macrocycle in *cone* conformation stabilized DNA and prevented its degradation.

## 1. Introduction

In recent years, research aimed at developing DNA vaccines has shown great potential [1]. These medicinal products are already successfully used in veterinary medicine [2]. Recently, Inovio Pharmaceuticals Corporation (Plymouth Meeting, PA) proposed a DNA vaccine for the prevention of SARS COVID-19 [3]. A significant increase in research on the use of DNA-based drugs for the treatment of other diseases has also been noted in recent years [4]. Wound-healing, anti-inflammatory, and antithrombotic drugs have been clinically tested and are now commercially available. For example, Jazz Pharmaceuticals proposed the drug Defitelio^®®^ (defibrotide sodium), which is the oligonucleotide obtained by depolymerization of porcine intestinal mucosal DNA for the treatment of hepatic veno-occlusive disease [5]. In addition, polynucleotides obtained from marine fish exert a wide therapeutic use [6,7,8].

The area of scientific research devoted to the development of DNA storage systems has become important due to the widespread application of DNA macromolecules [9]. Preservation of nucleic acid-based drugs from enzymatic degradation in the bloodstream is considered an important point [10]. The necessity of the storing of biomolecules is not limited to disease therapy. Intensive research has been carried out recently into preserving genetic material long-term to establish kinship and store information [11,12]. Among the methods of DNA storing, freezing is of great importance. However, chemical encapsulation is under intensive study as well. It is well known that DNA is able to bind with small molecules in three different ways, i.e., by electrostatic interactions, intercalation, or groove binding. All of these are important in different applications [13,14,15]. It is obvious that the structure of DNA can be preserved most closely to the native one during binding via electrostatic interactions; therefore, this is the most convenient method for DNA storing.

**Figure 1:**
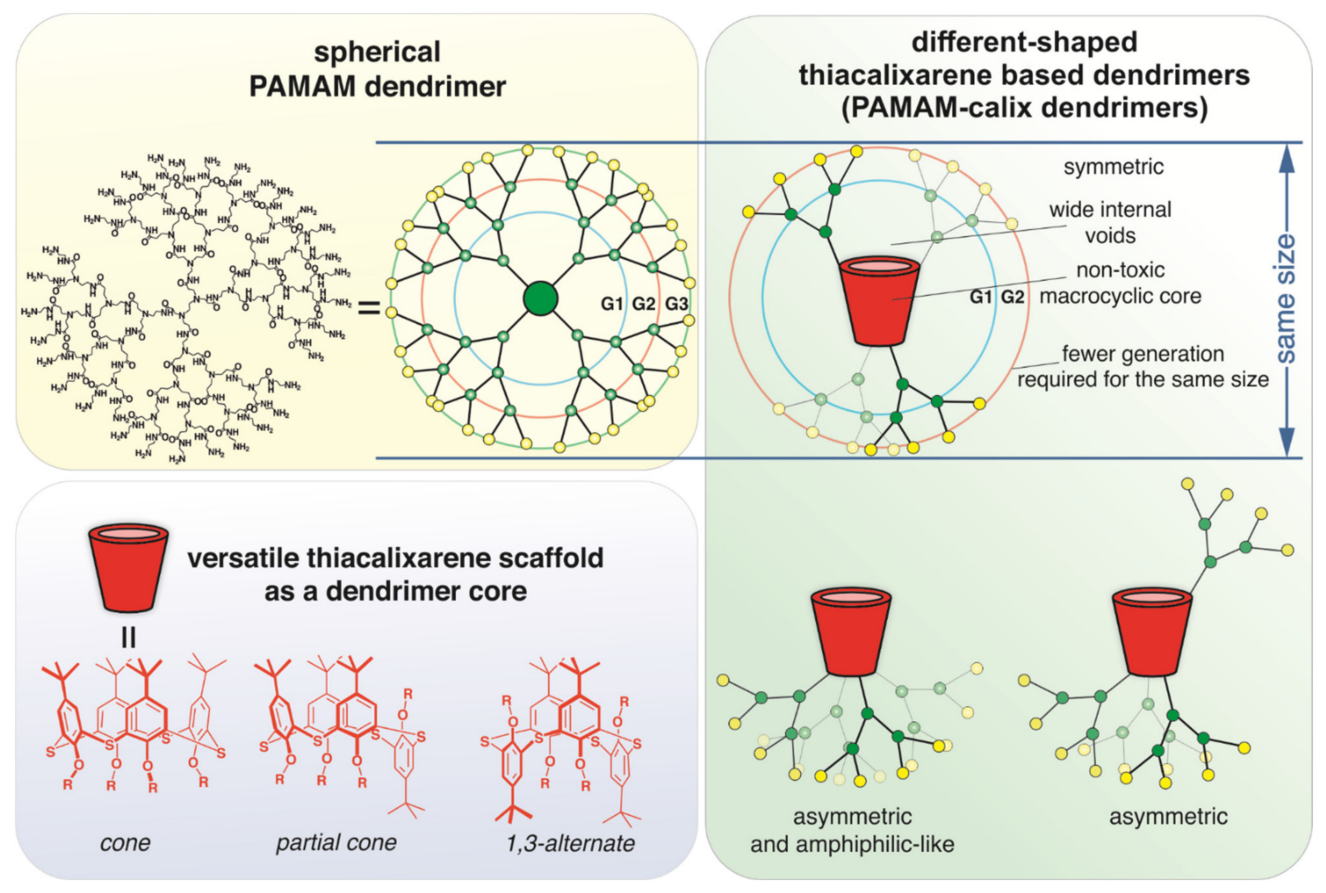
Schematic representation of the design of thiacalix[4]arene-based dendrimers.

Currently, synthetic non-viral DNA delivery and storage systems are intensively developing. Dendrimers capable of forming dendriplexes with DNA are the most promising variants of the synthetic vectors for DNA. Their ability to pH-responsive osmotic swelling and endosomal membrane rupture is undoubtedly important for the transfection of genetic material [10,16]. The search for new dendrimeric compounds with fewer disadvantages is an important area of synthetic chemistry. Macrocyclic compounds such as cyclodextrins, pillararenes, and the (thia)calixarenes with positively charged groups are promising for use as a dendrimer core for binding DNA as a biological polyanion [17,18,19]. We used thiacalix[4]arene as a core of dendrimer compounds. (Thia)calixarene scaffold is non-toxic. It is known that the covalent binding of various toxic molecules with (thia)calixarene scaffold decreases their toxicity [20,21]. The possibility of easy synthesis of stereoisomers with different spatial arrangements of substituents is another advantage of the thiacalixarene platform. Relatively rigid spatial fixation of binding groups provides high selectivity of guest molecule binding [22,23,24]. It should be noted that the thiacalixarene scaffold exerts a multivalent binding effect [25,26] by fixing binding sites in space, which is very important in the case of DNA [27]. The use of thiacalix[4]arene in *cone*, *partial cone*, and *1,3-alternate* conformation will lead to different shapes of the resulting dendrimers. True symmetric dendrimer shape in case of the core with the macrocycle in *1,3-alternate* conformation, as well as asymmetrical shape [10] in case of those in *cone* and *partial cone* conformation, can be expected (Figure 1). In the dendrimer with the core in *cone* conformation (Figure 1), the hydrophilic dendritic part and lipophilic macrocyclic part will be strictly parted. The uniqueness of this structure is that it is amphiphilic, contrary to classical dendrimers. By using dendrimers of a different shape described above for the DNA binding, we will be able not only to achieve the multivalency effect of binding but also to obtain dendriplexes of different structures.

Previously, we established the binding of salmon sperm DNA by thiacalix[4]arene derivatives containing four amidoammonium fragments at the lower rim resulted in the formation of associates of the size within 200 nm [17]. In this work, first-generation dendrimers based on the macrocycle (PAMAM-calix-dendrimers **G1**) with a large number of binding sites (12 amide and 12 ammonium fragments) were synthesized for the first time. The interaction of the obtained compounds with DNA was studied to establish the mechanism and the effect of the conformation (*cone*, *partial cone*, and 1,3-*alternate*) on the DNA binding and stability over time.

## 2. Results and Discussion

### 2.1. Synthesis of (Poly)Amidoamine Dendrimers Based on the p-tert-Buthylthiacalix[4]arene (PAMAM-Calix-Dendrimer) in Different Conformations

Synthesis of the poly(amidoamine) (PAMAM) dendrimers is well described in the literature [28,29]. The divergent approach is the optimal one to their synthesis. It involves the use of repetitive sequences of reactions. First, the core containing primary amino groups is introduced into the Michael addition reaction with methyl acrylate for branching. Then, the obtained precursor (called half-generation) is involved in the reaction with an excess of the diamine. This leads to the formation of a full generation of the PAMAM dendrimer. Side reactions during the synthesis of dendrimers often cause defects in their structure. The literature [30,31] describes both the range of possible side reactions and the main methods for preventing their occurrence.

Despite a large number of advantages in the application of the PAMAM dendrimers [16,29], they have a number of significant disadvantages and limitations. The most significant is the increase of in vivo and in vitro cytotoxicity of dendrimers in the direction from lower to higher generation and, accordingly, the high toxicity of upper generations dendrimers [32]. Cytotoxicity of the high generation dendrimers is due to a large number of positive charges on their surface, which can induce morphological changes in cells or cause cell lysis by disrupting the integrity of the negatively charged phospholipid bilayers of the cell membranes [33]. The structure of dendrimeric molecules with classical core not capable of serious conformational changes is another limitation. Therefore, the shape of such dendrimer molecules is close to spherical. In addition, despite the fact that currently, PAMAM dendrimers are commercially available up to generation 10, the cost of upper generation (G > 3) dendrimers is high [34].

The size of thiacalix[4]arene [22] is comparable to that of the PAMAM dendrimers of lower generations with ethylenediamine (EDA) core [29]. The PAMAM dendrimers of 1st–3rd generation with thiacalix[4]arene core are assumed to be comparable in size with the dendrimers with the EDA core of higher generations. In addition, the interior void space of the thiacalix[4] arene-based dendrimer will be significantly larger. This allows obtaining dendrimers that can effectively bind biopolymers and other guest molecules using fewer steps of the synthesis.

Our approach combines the advantages of dendrimers and thiacalixarenes and reduces their disadvantages. It will make it possible to obtain low-toxic dendrimer macromolecules with a wide internal void that are capable of efficient multi-point interaction with substrates.

There are few examples of the synthesis of low generation dendrimers based on classical calix[4]arene in the literature [35,36]. They are limited by the use of calixarene in one spatial conformation, either *cone* or *1,3-alternate*. It is also worth noting that there are no examples of the synthesis of high generation (G > 2) PAMAM dendrimers based on the thiacalix[4]arene platform.

In one recent publication [37], the authors described an improved approach to the synthesis and purification of the PAMAM dendrimers with a 1,4-diaminobutane core based on Tomalia’s previous works [29,38]. This approach allows to minimize the probability of side reactions and to obtain the PAMAM dendrimers in high yields. We have adapted this method to the synthesis of thiacalixarene-based dendrimers.

**Scheme 1:**
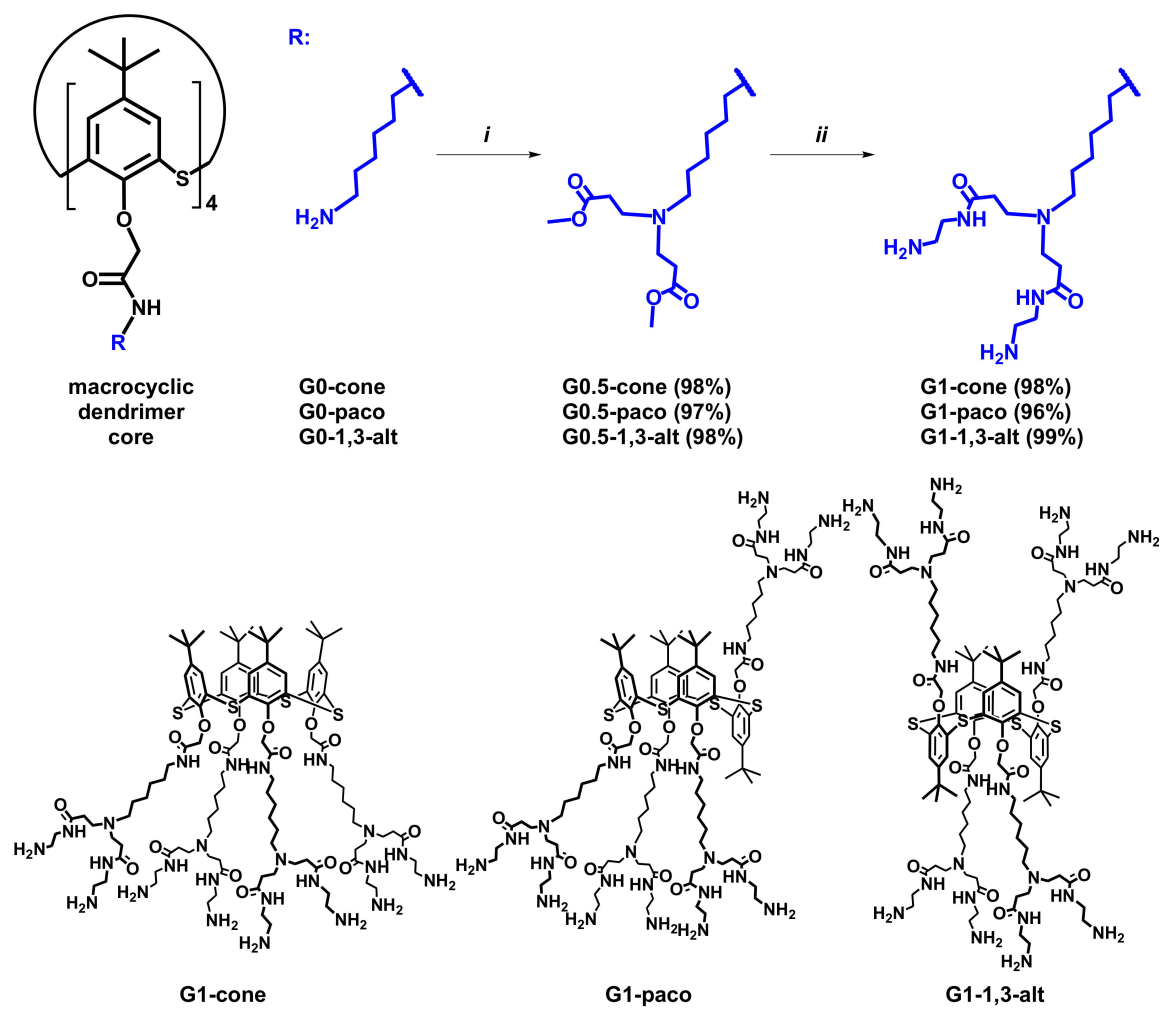
Reagents and conditions: (*i*) methyl acrylate, methanol, r.t., 12 h; (*ii*) ethylenediamine, methanol, r.t., 70 h.

We have chosen the macrocyclic compounds **G0** in *cone* (**G0-cone**), *partial cone* (**G0-paco**), and *1,3-alternate* (**G0-1,3-alt**) conformation [26] as cores for the synthesis of the PAMAM dendrimers based on thiacalix[4]arene. Hereby they are named as PAMAM-calix-dendrimers. Starting compounds contain four primary amino groups in their structure. This makes it possible to effectively apply a divergent approach for the dendrimer synthesis (Scheme 1). In order to minimize the possibility of side reactions, the temperature did not exceed 30 °C at all stages of the synthesis, isolation, and purification.

At the first stage of the synthesis, the compounds **G0-cone**, **G0-paco,** and **G0-1,3-alt** were involved in the reaction with methyl acrylate for branching creation. The reaction was carried out for 12 h at room temperature. As a result, the half-generation of PAMAM-calix-dendrimers, the compounds **G0.5-cone**, **G0.5-paco**, and **G0.5-1,3-alt**, were obtained with high yields (97–98%). Synthesis of the first generation dendrimer was the second stage. We used a large EDA excess (20 EDA equivalents per one ester group) to prevent side reactions of intra- and intermolecular crosslinking. Also, the complete removal of the EDA excess after the reaction is important in the synthesis of the PAMAM dendrimers. Otherwise, the residual EDA leads to trailing generation dendrimers due to the reaction between the EDA and methyl acrylate in the next stages. Taking these factors into account, a solution of the compounds **G0.5-cone**, **G0.5-paco**, and **G0.5-1,3-alt** in methanol was added to that of EDA in ice-cooled methanol. The reaction mixture was stirred for 70 h to ensure complete reaction of all the ester groups. Typically, azeotropic distillation in methanol:toluene (1:9) mixture was used to remove EDA from the reaction mixture completely [26,38]. After removal of the solvent and EDA from the reaction mixture, the residue was dissolved in methanol:toluene mixture, and all volatiles was removed under reduced pressure. This procedure was repeated seven times. Then the excessive toluene was removed by azeotropic distillation with methanol. As a result, the PAMAM-calix-dendrimers of the first generation, the compounds **G1-cone**, **G1-paco**, and **G1-1,3-alt**, were obtained (Scheme 1) with quantitative yields (96–99%).

Thus, an efficient method for the synthesis of the first generation PAMAM-calix-dendrimers dendrimers **G1** and their precursors (half-generation) **G0.5** has been developed. The structure and composition of the obtained compounds **G0.5** and **G1** were confirmed by ^1^H, ^13^C NMR, IR spectroscopy, mass spectrometry, and elemental analysis (Figures S1–S24, See Supplementary Materials).

NMR spectroscopy is a classic technique for establishing the structure of macromolecules as well as for evaluating the purity of these compounds [39]. The PAMAM dendrimers have a low number of signals in the ^1^H and ^13^C NMR due to their highly symmetrical and regular structure. It is known that various conformations of thiacalix[4]arene differ in chemical shifts of the proton signals due to the influence of the macrocyclic platform [39,40,41]. That allows to unequivocally determine the conformation of the macrocycle by ^1^H NMR spectroscopy. The strongest differences were observed in the chemical shifts of the proton signals of amidomethylene and oxymethylene fragments closest to the *p*-tert-butylthiacalix[4]arene platform. In the case of the amphiphilic-like compound **G1-cone** with asymmetric structure (Figure 1), these signals were located at 3.33 and 4.89 ppm, respectively (Figure S13). In the case of symmetric dendrimer **G1-1,3-alt**, these groups were shielded by neighboring aromatic rings of the macrocycle, and their signals appeared at 3.25 and 4.15 ppm (Figure S21). Smaller differences were observed in the chemical shifts of the proton signals of the *tert*-butyl and aryl fragments. Compared to **G1-1,3-alt**, chemical shifts of the aryl protons in the **G1-cone** drifted upfield (7.45 ppm for **G1-cone** against 7.59 ppm for **G1-1,3-alt**) due to the shielding effect of neighboring aryl fragments on the aryl protons of the macrocycle ring. Similarly, the peaks of the *tert*-butyl groups of the **G1-cone** were also shifted upfield (1.14 ppm) compared to corresponding peaks for the **G1-1,3-alt** (1.28 ppm).

In the case of the asymmetric compound **G1-paco**, symmetry of the macrocyclic core was disturbed, and this complicated the spectra. The peaks of the *tert*-butyl fragment appeared as two singlets at 1.09 and 1.35 ppm with a ratio of 1:1, peaks of protons of aromatic and oxymethylene groups formed two singlets and AB system (Figure S17).

It should be noted that many chemical shifts of branching proton signals coincided in comparison to the ^1^H NMR spectra of three compounds **G1-cone**, **G1-paco**, and **G1-1,3-alt**. This occurs even in the NMR spectrum of the compound **G1-paco**, although the NMR spectra of the thiacalix[4]arene derivatives in *partial cone* conformation are usually complicated due to the disturbance of the symmetry of the macrocyclic platform. Thus, in all three stereoisomers, the proton signals of the methylene groups of branching appeared in the same areas (2.36, 2.46, 2.65–2.80, and 3.24 ppm). This is probably due to the decreasing influence of the macrocycle conformation resulting from the distancing of mentioned groups from the macrocyclic platform. Besides, the obtained NMR data indicates the purity of the synthesized first-generation PAMAM-calix-dendrimers and the absence of defects in their structure.

The obtained compounds **G0.5** and **G1** were also characterized by mass spectrometry (Figures S3, S7, S11, S15, S19 and S23). For example, ESI high-resolution mass spectra of the compounds **G1** showed peaks corresponding to molecular ions with three, four, five and six protons ([M + 3H]^3+^, [M + 4H]^4+^, [M + 5H]^5+^, and [M + 6H]^6+^).

Thus, the novel first generation of PAMAM-calix-dendrimers was designed and synthesized with high yields. The structure of the obtained compounds was proven by modern physical methods.

### 2.2. The Study of Binding of the Obtained PAMAM-Calix-Dendrimers G1 with Salmon Sperm DNA and the Effect on DNA Temporal Stability

**Figure 2:**
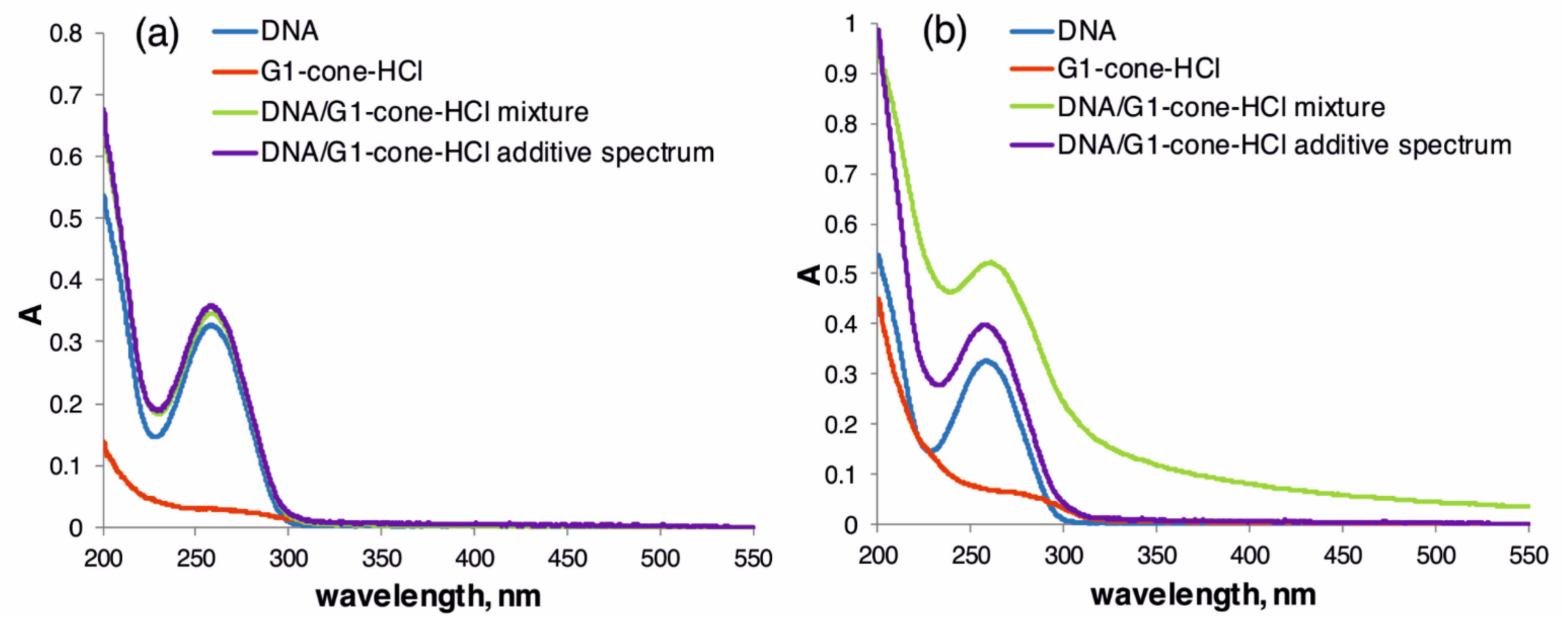
UV-Vis absorption spectra of DNA (1.855 × 10^−5^ M base pairs), **G1-cone-HCl** and **G1-cone-HCl**/DNA mixture in 10 mM Tris-HCl buffer (pH = 7.4) for **G1-cone-HCl** concetrations: (**a**) 8.33 × 10^−7^ M; (**b**) 3.33 × 10^−5^ M.

It is well known that polyamines are capable of DNA condensation with the formation of the associates of the diameter of about 100 nm. They can be used to deliver a biomolecule into a cell [42]. In this regard, we first applied the UV-Vis spectroscopy method to establish the possibility of interaction between the compounds **G1** (**G1-cone**, **G1-paco,** and **G1-1,3-alt**) and DNA. We have chosen salmon sperm DNA, which is widely used as a model biomolecule [6,17,18]. The amine groups of the studied compounds **G1** were converted into the cationic form (**G1-HCl**) by reaction with hydrochloric acid in order to increase the affinity to the biopolymer. DNA has maximum absorption in the region up to 300 nm [17,18]. The fact that the synthesized compounds have absorption due to π-π* transitions of aromatic rings of the platform and n-π* transitions of carbonyl groups in the same range (200–315 nm) complicated the study [26]. The UV-Vis spectra of the **G1-cone-HCl** and **G1-paco-HCl** are identical (Figure S25), while the UV-Vis spectrum of the **G1-1,3-alt-HCl** differs by the presence of a typical shoulder for this conformation (Figure S25) [26,43]. The hypochromic effect observed in the absorption spectra of all obtained compounds in the presence of DNA indicates their interaction with DNA (Figure 2 and Figures S26–S32). In the case of very dilute solutions (8.33 × 10^−7^ M) of **G1-HCl** compounds, a weak hypochromic effect was observed (Figure 2a). However, with an increase in the concentration of the compounds **G1-HCl** (3.33 × 10^−5^ M and 3.33 × 10^−6^ M), the spectra started being significantly complicated because of an increase in the baseline of the spectrum in the long-wavelength region. This additionally indicated that the association of the macrocycles with the biomolecule resulted in the formation of the nanoparticles (Figures S28–S32) [18,44]. Such character of spectral changes is common for biopolymers when they bind by macrocyclic compounds [45,46]. Extrapolation of the linear fragment of the baseline made it possible to account for the contribution of the Rayleigh light scattering to the spectral pattern [47] and to establish a retaining hypochromic effect. Thus, the observed deviation from the additivity of the spectra of individual components of the mixture confirms the interaction of the ammonium forms of the synthesized dendrimers with DNA.

Further, DLS experiments confirm the formation of the nanosized PAMAM-calix-dendrimer/DNA aggregates. It was observed that the ammonium derivatives of the G1-HCl did not form stable self-associates in the concentration range 5 × 10^−4^–5 × 10^−6^ M (Table S1). However, in the presence of salmon sperm DNA (0.0384 mg/mL, 5.565 × 10^−5^ M base pairs), monodisperse systems with small particle sizes were formed (Table 1, Figures S55–S69). This correlates well with the known fact that a condensed DNA state prevails in the dendriplexes [16]. The largest particles (265 nm) were formed in the case of the **G1-1,3-alt-HCl** (5 × 10^−5^ M, PDI 0.162) (Figure S57). For the **G1-paco-HCl**, the largest particles were also observed at 5 × 10^−5^ M (Figure S67). Meanwhile, the **G1-cone-HCl** was an exception, with the maximum size of the particles at 1 × 10^−5^ M (Figure S63). The size of the particles remained close and lower than 200 nm for all the studied concentrations. Undoubtedly, this fact deserves attention because particles with the size of 30–200 nm (maximum 500 nm) are allowed for medical purposes [48,49]. Macrophages do not react with such particles, and meanwhile, they are large enough not to filter in the kidneys [48,49]. Measurement of the electrokinetic ζ-potentials showed that the formed particles were stable. At high concentrations of **G1-HCl,** they were positively charged. With decreasing **G1-HCl** concentration, the charge of the associates changed to a negative one (Table 1, Figures S39–S53).

**Figure 3:**
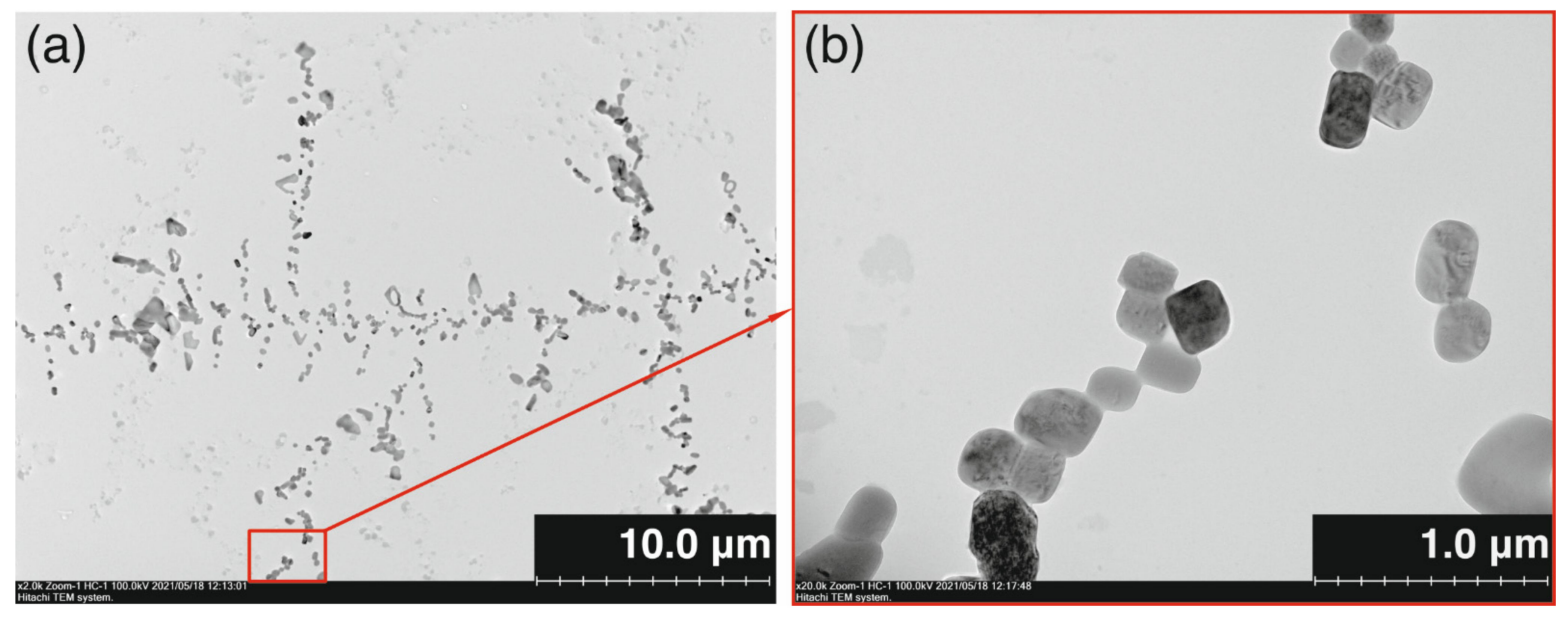
TEM images of associates **G1-cone-HCl**/DNA (5 × 10^−5^ M of **G1-cone-HCl**, 5.565 × 10^−5^ M DNA base pairs). Scale bar (**a**) 10 μm; (**b**) 1 μm.

The formation of the nanoparticles was also confirmed by TEM. The TEM images of the PAMAM-calix-dendrimer **G1-cone-HCl** with DNA showed that the particles with the size close to 200 nm coalesced with each other (Figure 3). However, the TEM images of individual solutions of the DNA and the **G1-cone-HCl** were absolutely different from the images of their mixture (Figures S70 and S71). They contained amorphous particles with high polydispersity. This was in good agreement with the DLS data (Table S1).

**Figure 4:**
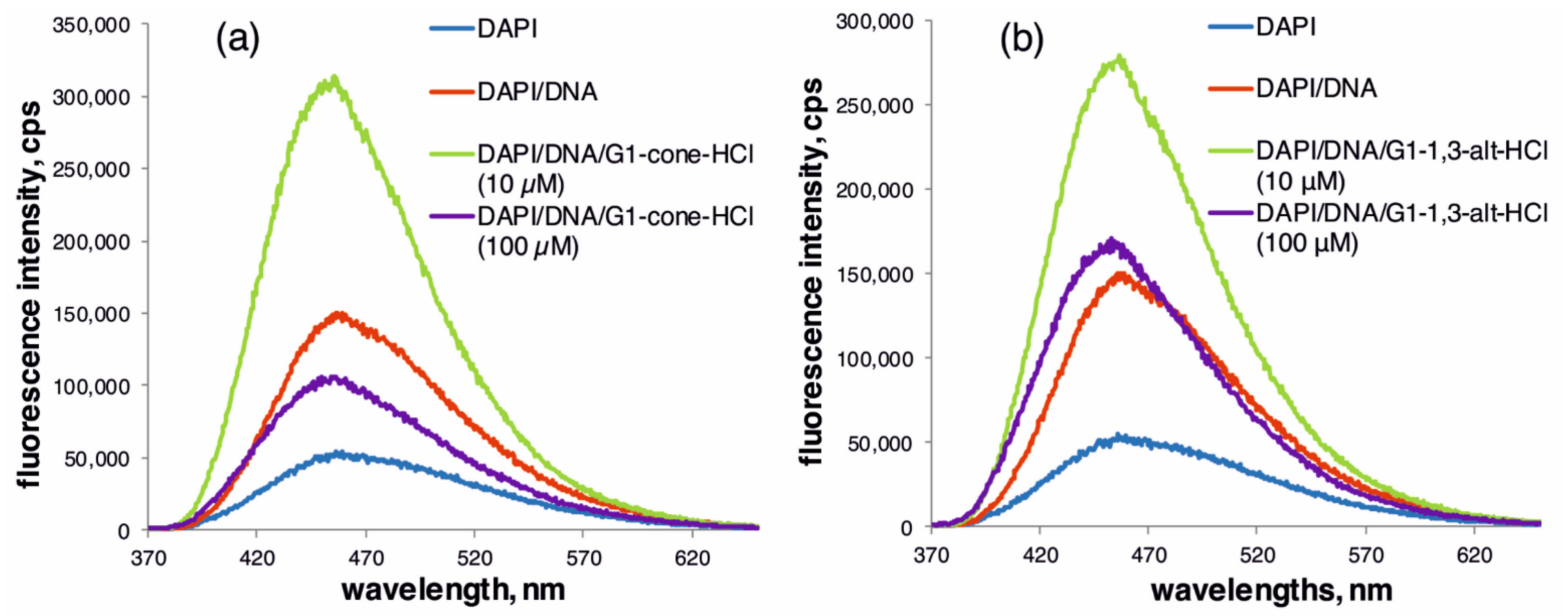
Fluorescence spectra of DAPI (1 × 10^−5^ M), DAPI/DNA (5.565 × 10^−5^ M base pairs) and DAPI/DNA in the presence of 10 μM and 100 μM of: (**a**) **G1-cone-HCl** and (**b**) **G1-1,3-alt-HCl**, in 10 mM Tris-HCl buffer, pH = 7.4.

In order to confirm the interaction between the compounds **G1-HCl** and DNA, fluorescence spectroscopy was also applied. It is widely used to study a variety of systems [50]. Since DNA and the studied compounds **G1-HCl** do not possess fluorescent properties, we chose the DAPI fluorescent dye with an emission maximum at 462 nm. It is known that DAPI is capable of binding to both DNA grooves and via intercalation [51,52]. Taking into account the DLS data, we selected several concentrations of the PAMAM-calix-dendrimers, at which associates with different charges are formed. The DAPI fluorescence spectra recorded in the presence of 10 μM and 100 μM concentrations of **G1-HCl** compounds showed no effect on the dye emission (Figure S35). However, the addition of low concentrations (1 × 10^−5^ M) of **G1-HCl** compounds in all conformations of the dendrimer core to the DAPI mixed with DNA results in the fluorescence enhancement (Figure 4 and Figure S37). We have demonstrated such behavior for the systems of DNA with polyelectrolytes in our previous work [18]. This is explained by the shielding of the dye bonded to the DNA by ammonium derivatives from the aqueous medium. Unexpectedly, an increase in the concentration of the **G1-HCl** to 1 × 10^−4^ M dramatically changed the spectrum, and emission quenching was observed (Figure 4, Figures S36 and S37).

The largest change in the fluorescence spectra was observed for the **G1-cone-HCl**. In this case, the emission intensity turned out to be even lower than that of the DAPI/DNA system in the absence of the PAMAM-calix-dendrimer (Figure 4). It can result from displacing the DAPI molecules by the macrocycles from the DNA groove. This mechanism explains quenching of the DAPI fluorescence in most works [53,54,55]. Thus, we assume that the **G1-HCl** form at least two types of complexes with DNA, i.e., by binding to the biopolymer at phosphate groups and in the groove. According to the literature, the formation of the dendriplexes with DNA is possible via the binding of terminal amino groups of the PAMAM dendrimers to the phosphate groups of nucleic acids at physiological pH [56]. The structure of the dendrimers makes intercalation impossible. It is also important to note that changing the order of the components addition, namely, the **G1-HCl** solution added to the already formed DAPI/DNA complex (Figure 4 and Figure S37), or DAPI added to the **G1-HCl**/DNA complex (Figure S36), did not significantly affect the results. This indicates the equilibrium of all three components of the mixture.

**Figure 5:**
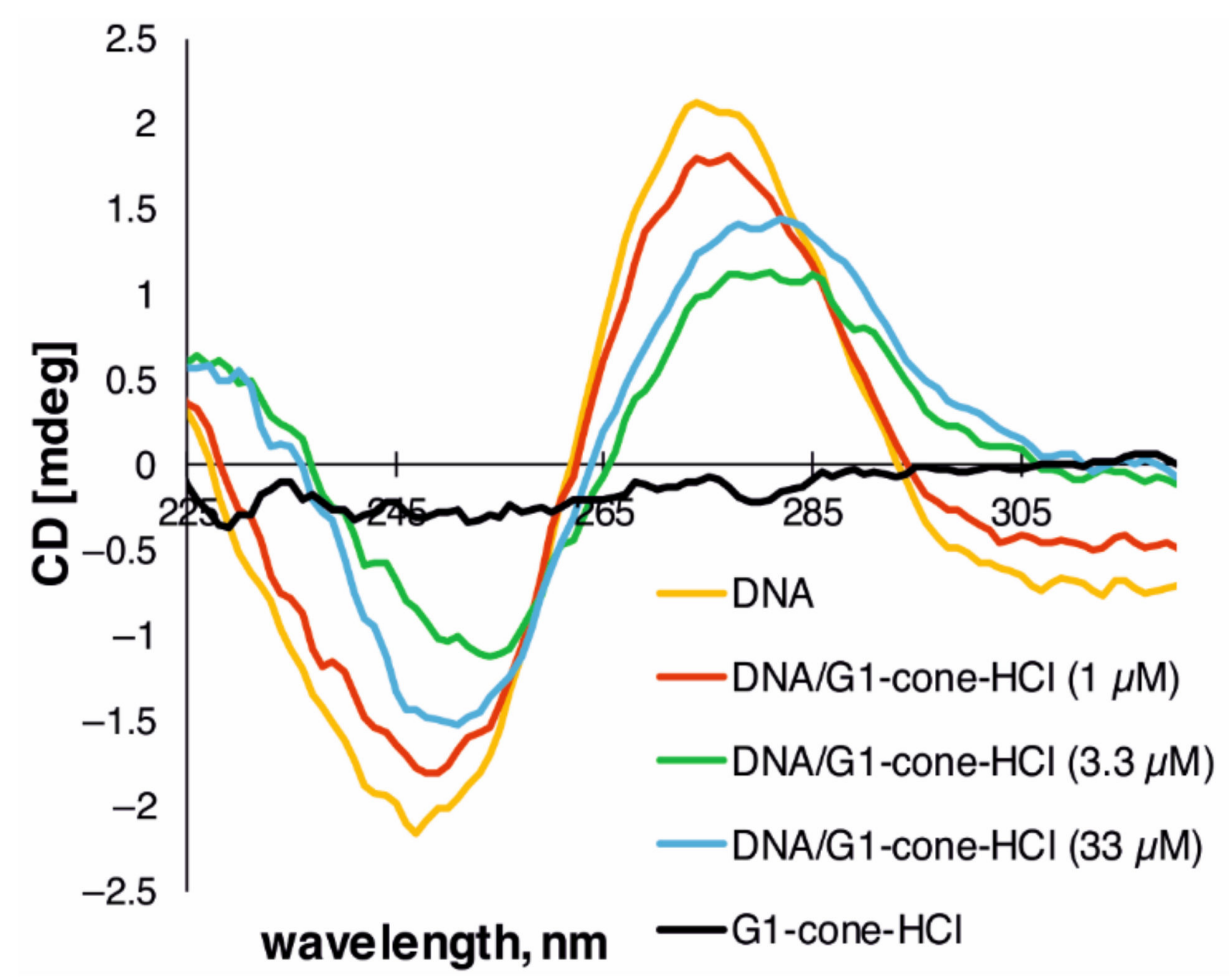
CD spectra of DNA (1.855 × 10^−5^ M base pairs) in the presence of various concentrations of **G1-cone-HCl**.

Additional confirmation of the absence of intercalation was obtained by CD spectroscopy. It is a convenient method for tracking changes in the DNA structure [47]. The CD spectrum of the DNA had a positive signal at 274 nm and a negative one at 247 nm, which is typical for the mixture of canonical A- and B-forms of DNA [57,58] (Figure 5). The investigated compounds **G1-HCl** had no signals in the CD spectra since they are devoid of chirality. When the compounds **G1-HCl** (1 × 10^−6^ M) were added to the DNA, a slight decrease in the amplitude of the values of both signals was observed, which indicates a decrease in the asymmetry of the biopolymer molecule. In addition, a slight red shift of both maxima was observed (from 274 to 284 nm and from 247 to 255 nm). As in the fluorescence spectra, the largest changes at the concentration of 1 × 10^−6^ M were found in the case of the **G1-cone-HCl**. The addition of the macrocycles did not lead to significant changes in the structure of the DNA while its canonical forms prevailed. Thus, such small changes in the CD spectra allow us to state that the intercalation of the studied compounds into the biopolymer is not carried out [59,60]. It is interesting to note that the CD spectra, like the fluorescence spectra, changed differently with the increasing concentration of the **G1-HCl**. At first (3.3×10^−6^ M of **G1-HCl**), a further decrease in the signal amplitude at 274 nm was observed. However, at a macrocycle concentration of 3.3 × 10^−5^ M, an unexpected increase in the amplitude of the positive signal occurred with a further red shift of the band of the spectra (Figure 5 and Figure S38). This correlates well with the data obtained by fluorescence spectroscopy. As a result, the minimal effect of the **G1-HCl** on the CD spectra of the DNA indicates their external binding to the DNA phosphate groups [18,53,54]. Change in the nature of the effect (hyperchromia) with a growth in the bathochromic shift with an increase of the **G1-HCl** concentration made it possible to conclude that the structure of the complexes changed due to further binding along the DNA groove [55].

**Figure 6:**
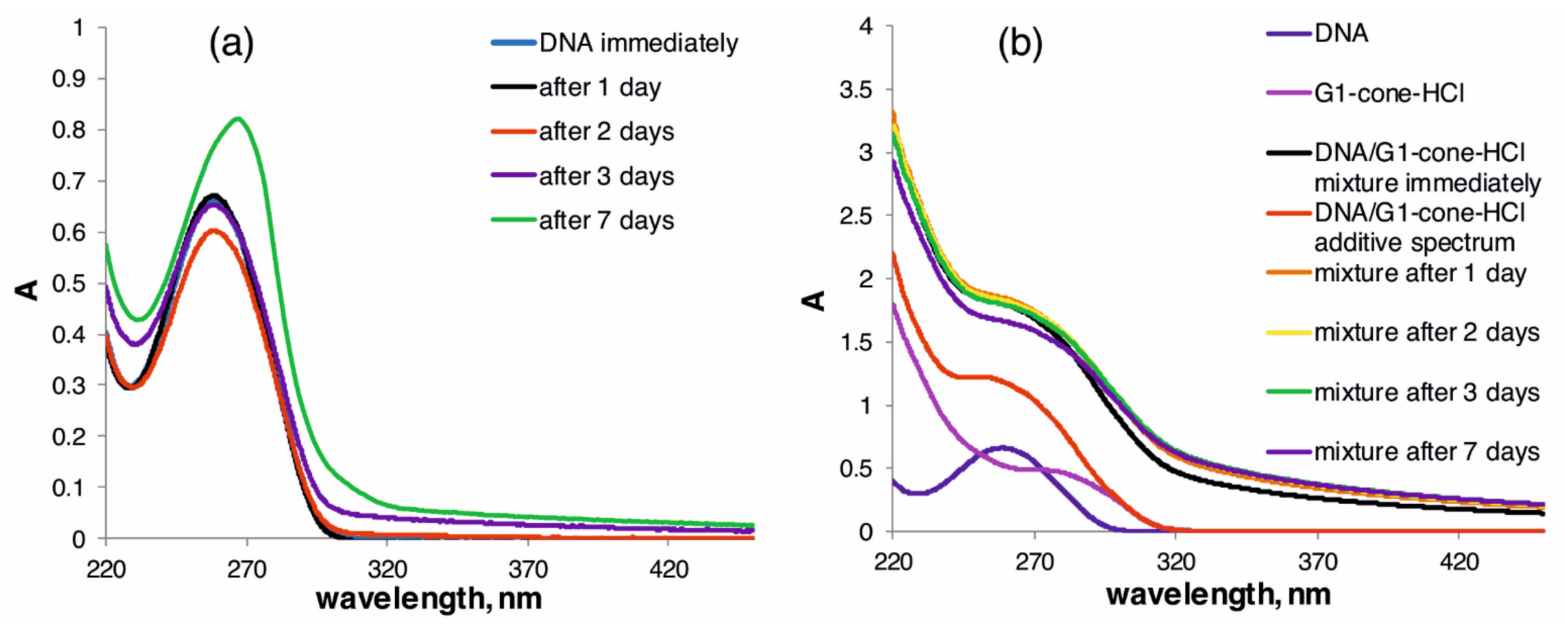
UV-Vis spectra of (**a**) pure DNA (3.710 × 10^−5^ M base pairs), (**b**) DNA (3.710 × 10^−5^ M base pairs) in the presence of **G1-cone-HCl** (10 mM Tris-HCl buffer, pH 7.4).

Since it was established by the CD spectroscopy that the binding of the compounds **G1-HCl** did not significantly change the DNA structure, we suggested that such binding could lead to stabilization of the biopolymer structure and protect it from degradation. In order to confirm this, we carried out an additional study of the DNA solutions with the PAMAM-calix-dendrimers over time at room temperature by the UV-Vis spectroscopy (Figure 6). In the case of pure DNA, at first, a slight decrease in the optical density of the solution was observed within three days. Then, optical density significantly increased obviously due to the degradation of the biomolecule [47]. Meanwhile, in the case of the **G1-cone-HCl**, the spectra remained constant for seven days (Figure 6). This indicates the stability of the formed complexes. For the other two compounds (**G1-paco-HCl** and **G1-1,3-alt-HCl**), the structure of the complexes noticeably changed over time, as followed by a significant change in the UV-Vis spectra of their mixtures with DNA (Figure S34). It should also be noted that the spectra of all individual compounds **G1-HCl** remained constant for seven days (Figure S33). Thus, binding of the DNA to the **G1-cone-HCl** prevents DNA degradation that the biopolymer can undergo due to the action of nucleases and blood serum components by reducing the negative surface charge [61,62], as shown by electrophoretic light scattering (Table 1). An important point here is the fundamental difference between the structure of the **G1-cone-HCl** and other compounds. We assume that the presence of a lipophilic (hydrophobic) part in the **G1-cone-HCl** molecule isolated DNA from an aqueous solution, thereby protecting DNA from the effects of destructive factors.

Thus, it was shown that all the obtained PAMAM-calix-dendrimers are capable of efficient binding with the salmon sperm DNA, thus forming nanoparticles with a size of up to 200 nm. It was also shown that the compound **G1-cone-HCl** is capable of stabilizing the DNA molecules for seven days. It is important to note the differences between our synthesized PAMAM-calix-dendrimers and classical PAMAM dendrimers. It is well known that dendrimers effectively interact with the DNA only at their optimal generations (usually 4–5 generations) because low generations of dendrimers interact weakly with biopolymer due to a low surface charge [56,63,64]. The replacement of the classical amine core of PAMAM dendrimers with the macrocyclic thiacalixarene has shown the prospects of using the proposed PAMAM-calix-dendrimers already in the first generation for binding, stabilizing, and storing DNA.

## 3. Materials and Methods

### 3.1. General Experimental Information

All chemicals were purchased from Acros (Fair Lawn, NJ, USA), and most of them were used as received without additional purification. Ethylenediamine (EDA) and organic solvents were purified by standard procedures. ^1^H NMR and ^13^C NMR spectra were obtained on the Bruker Avance-400 spectrometer (Bruker Corp., Billerica, MA, USA) (^13^C{^1^H} 100 MHz and ^1^H 400 MHz). Chemical shifts were determined against the signals of residual protons of deuterated solvent (CDCl_3_, CD_3_OD). Concentrations of the compounds were equal to 3–5% in all the records. The FTIR ATR spectra were recorded on the Spectrum 400 FT-IR spectrometer (Perkin–Elmer, Seer Green, Llantrisant, UK) with the Diamond KRS-5 attenuated total internal reflectance attachment (resolution 0.5 cm^−1^, accumulation of 64 scans, recording time 16 s in the wavelength range 400–4000 cm^−1^). Elemental analysis was performed on the Perkin–Elmer 2400 Series II instruments (Perkin–Elmer, Waltham, MA, USA). Melting points were determined using the Boetius Block apparatus (VEB Kombinat Nagema, Radebeul, Germany). The MALDI mass spectra were recorded on the Ultraflex III mass spectrometer (Bruker Daltonik GmbH, Bremen, Germany). *p*-Nitroaniline was used as the matrix. ESI HRMS experiments were performed at Agilent 6550 iFunnel Q-TOF LC/MS (Agilent Technologies, Santa Clara, CA, USA), equipped with Agilent 1290 Infinity II LC.

The dendrimer cores **G0** (thiacalix[4]arene derivatives in *cone* (**G0-cone**), *partial cone* (**G0-paco**), and *1,3-alternate* (**G0-1,3-alt**) conformation) were synthesized by the previously described procedure [26].

### 3.2. General Procedure for the Synthesis of the Compounds G0.5 (Generation 0.5 Dendrimers with Thiacalix[4]arene Core in Cone (G0.5-cone), Partial Cone (G0.5-paco) and 1,3-Alternate (G0.5-1,3-Alt) Conformation)

Methyl acrylate (1.65 mL, 18 mmol) was added to the solution of the compound **G0** (1.55 g, 1.15 mmol) in methanol (35 mL). The reaction mixture was stirred for 12 h at room temperature. Then the solvent and the excess methyl acrylate were removed under reduced pressure on the rotary evaporator. The residue was dried under reduced pressure.

#### 3.2.1. G0.5-cone, 5,11,17,23-Tetra-*tert*-butyl-25,26,27,28-tetrakis[*N*-(6-(*N*,*N*-di(methoxycarbonylethyl)amino)hexyl)carbamoylmethoxy]-2,8,14,20-tetrathiacalix[4]arene

Translucent yellowish oil, yield: 2.31 g (98%).

^1^H NMR (CDCl_3_, δ, ppm, *J*/Hz): 1.10 (s, 36H, (CH_3_)_3_C), 1.28 (m, 16H, C(O)NHCH_2_CH_2_CH_2_CH_2_, C(O)NHCH_2_CH_2_CH_2_CH_2_), 1.39 (m, 8H, CH_2_CH_2_N), 1.56 (m, 8H, C(O)NHCH_2_CH_2_CH_2_CH_2_), 2.30–2.46 (m, 24H, CH_2_CH_2_N, NCH_2_CH_2_C(O)OCH_3_), 2.73 (m, 16H, NCH_2_CH_2_C(O)OCH_3_), 3.33 (m, 8H, C(O)NHCH_2_), 3.64 (s, 24H, C(O)OCH_3_), 4.80 (s, 8H, OCH_2_C(O)), 7.33 (s, 8H, Ar-H), 7.85 (br.t, 4H, C(O)NH).

^13^C NMR (CDCl_3_, δ, ppm): 27.15, 27.19, 27.25, 29.85, 31.21, 32.57, 34.40, 39.52, 49.27, 51.68, 53.81, 74.69, 128.03, 135.02, 147.62, 157.75, 168.18, 173.23.

FTIR ATR (ν, cm^−1^): 3357 (N-H), 3300 (N-H), 1734 (C=O), 1652 (C(O)NH, Amide I), 1551 (C(O)NH, Amide II), 1248 (C(O)NH, Amide III), 1036 (C_Ph_OCH_2_).

Elemental analysis. Calculated for C_104_H_160_N_8_O_24_S_4_ C, 61.39; H, 7.93; N, 5.51; S, 6.30; Found: C, 61.24; H, 8.03; N, 5.61; S, 6.19.

MALDI, Calculated [M + H]^+^ = 2035.053. Found [M + H]^+^ = 2035.078.

#### 3.2.2. G0.5-paco, 5,11,17,23-Tetra-*tert*-butyl-25,26,27,28-tetrakis[*N*-(6-(*N*,*N*-di(methoxycarbonylethyl)amino)hexyl)carbamoylmethoxy]-2,8,14,20-tetrathiacalix[4]arene

Translucent yellowish oil, yield: 2.28 g (97%).

^1^H NMR (CDCl_3_, δ, ppm, *J*/Hz): 1.03 (s, 18H, (CH_3_)_3_C), 1.20–1.50 (m, 24H, C(O)NHCH_2_CH_2_CH_2_CH_2_, C(O)NHCH_2_CH_2_CH_2_CH_2_, CH_2_CH_2_N), 1.29 (s, 9H, (CH_3_)_3_C), 1.32 (s, 9H, (CH_3_)_3_C), 1.63 (m, 8H, C(O)NHCH_2_CH_2_CH_2_CH_2_), 2.29–2.45 (m, 24H, CH_2_CH_2_N, NCH_2_CH_2_C(O)OCH_3_), 2.66–2.78 (m, 16H, NCH_2_CH_2_C(O)OCH_3_), 3.12 (m, 2H, C(O)NHCH_2_), 3.22–3.33 (m, 2H, C(O)NHCH_2_), 3.34–3.46 (m, 4H, C(O)NHCH_2_), 3.58–3.69 (m, 24H, C(O)OCH_3_), 4.35 (d, 2H, OCH_2_C(O), ^2^*J*_HH_ = 14.7), 4.42 (s, 2H, OCH_2_C(O)), 4.84 (d, 2H, OCH_2_C(O), ^2^*J*_HH_ = 14.7), 4.96 (s, 2H, OCH_2_C(O)), 7.05 (d, 2H, Ar-H, ^4^*J*_HH_ = 2.2), 7.32 (br.t, 1H, C(O)NH), 7.47 (d, 2H, Ar-H, ^4^*J*_HH_ = 2.2), 7.62 (s, 2H, Ar-H), 7.78 (s, 2H, Ar-H), 7.91 (br.t, 2H, C(O)NH), 8.62 (br.t, 1H, C(O)NH).

^13^C NMR (CDCl_3_, δ, ppm): 26.84, 27.08, 27.16, 27.19, 27.37, 29.61, 29.90, 30.00, 31.01, 31.12, 31.21, 31.35, 31.40, 31.47, 32.56, 34.27, 34.38, 34.48, 39.09, 39.42, 39.59, 49.27, 51.67, 53.77, 53.82, 69.96, 74.01, 74.60, 125.70, 125.87, 127.45, 127.66, 133.41, 135.08, 135.29, 136.50, 146.56, 146.65, 147.54, 155.74, 158.19, 159.51, 168.06, 168.42, 168.73, 173.19, 173.21.

FTIR ATR (ν, cm^−1^): 3288 (N-H), 1734 (C=O), 1648 (C(O)NH, Amide I), 1543 (C(O)NH, Amide II), 1247 (C(O)NH, Amide III), 1035 (C_Ph_OCH_2_).

Elemental analysis. Calculated for C_104_H_160_N_8_O_24_S_4_ C, 61.39; H, 7.93; N, 5.51; S, 6.30; Found: C, 61.43; H, 7.76; N, 5.38; S, 6.27.

ESI, Calculated [M + 2H]^2+^ *m/z* = 1018.0, [M + 3H]^3+^ *m/z* = 679.0, [M + 4H]^4+^ *m/z* = 509.5. Found [M + 2H]^2+^ *m/z* = 1018.0, [M + 3H]^3+^ *m/z* = 679.0, [M + 4H]^4+^ *m/z* = 509.6.

#### 3.2.3. G0.5-1,3-alt, 5,11,17,23-Tetra-*tert*-butyl-25,26,27,28-tetrakis[*N*-(6-(*N*,*N*-di(methoxycarbonylethyl)amino)hexyl)carbamoylmethoxy]-2,8,14,20-tetrathiacalix[4]arene

^1^H NMR (CDCl_3_, δ, ppm, *J*/Hz): 1.21 (s, 36H, (CH_3_)_3_C), 1.30 (m, 16H, C(O)NHCH_2_CH_2_CH_2_CH_2_, C(O)NHCH_2_CH_2_CH_2_CH_2_), 1.41 (m, 8H, CH_2_CH_2_N), 1.59 (m, 8H, C(O)NHCH_2_CH_2_CH_2_CH_2_), 2.32–2.47 (m, 24H, CH_2_CH_2_N, NCH_2_CH_2_C(O)OCH_3_), 2.75 (m, 16H, NCH_2_CH_2_C(O)OCH_3_), 3.25 (m, 8H, C(O)NHCH_2_), 3.65 (s, 24H, C(O)OCH_3_), 4.06 (s, 8H, OCH_2_C(O)), 7.54 (s, 8H, Ar-H), 7.80 (br.t, 4H, C(O)NH).

^13^C NMR (CDCl_3_, δ, ppm): 27.21, 27.29, 27.35, 29.84, 31.23, 32.52, 34.42, 39.60, 49.29, 51.74, 53.83, 71.33, 127.22, 133.59, 147.43, 156.99, 168.14, 173.12.

FTIR ATR (ν, cm^−1^): 3318 (N-H), 1734 (C=O), 1666 (C(O)NH, Amide I), 1533 (C(O)NH, Amide II), 1263 (C(O)NH, Amide III), 1025 (C_Ph_OCH_2_).

Elemental analysis. Calculated for C_104_H_160_N_8_O_24_S_4_ C, 61.39; H, 7.93; N, 5.51; S, 6.30; Found: C, 61.12; H, 8.15; N, 5.71; S, 6.11.

MALDI, Calculated [M + H]^+^ *m/z* = 2035.053, [M + Na]^+^ *m/z* = 2057.036. Found [M + H]^+^ *m/z* = 2035.941, [M + Na]^+^ *m/z* = 2058.021.

### 3.3. General Procedure for the Synthesis of the Compounds G1 (Generation 1 Dendrimers with Thiacalix[4]arene Core in Cone (G1-Cone), Partial Cone (G1-Paco), and 1,3-Alternate (G1-1,3-alt) Conformation)

Freshly distilled EDA (7.09 mL, 106 mmol) was dissolved in methanol (7 mL) and cooled with an ice bath to 0 °C. Solution of the compound **G0.5** (1.35 g, 0.66 mmol) in methanol (10 mL) was added dropwise to the solution of EDA with the rate of one drop per three seconds. The ice bath was kept for another 1 h; afterward, the reaction mixture was stirred for 70 h at room temperature. After that, methanol was removed under reduced pressure. Excess of EDA was removed by azeotropic distillation with the mixture methanol:toluene (1:9). The procedure was repeated seven times. Then, the residue amount of toluene was removed by azeotropic distillation with methanol. The residue was dried under reduced pressure over phosphorous pentoxide.

#### 3.3.1. G1-cone, 5,11,17,23-Tetra-tert-butyl-25,26,27,28-tetrakis[N-(6-(N,N-di(N-(2-aminoethyl)carbamoylethyl)amino)hexyl)carbamoylmethoxy]-2,8,14,20-tetrathiacalix[4]arene

White solid foam, yield 1.47 (98%). M.p. 58 °C

^1^H NMR (CD_3_OD, δ, ppm, *J*/Hz): 1.14 (s, 36H, (CH_3_)_3_C), 1.35 (m, 16H, C(O)NHCH_2_CH_2_CH_2_CH_2_, C(O)NHCH_2_CH_2_CH_2_CH_2_), 1.48 (m, 8H, CH_2_CH_2_NH_2_), 1.60 (m, 8H, C(O)NHCH_2_CH_2_CH_2_CH_2_), 2.36 (m, 16H, NCH_2_CH_2_C(O)NH), 2.46 (m, 8H, CH_2_CH_2_N), 2.65–2.80 (m, 32H, CH_2_NH_2_, NCH_2_CH_2_C(O)NH), 3.24 (m, 16H, C(O)NHCH_2_), 3.33 (m, 8H, C(O)NHCH_2_), 4.89 (s, 8H, OCH_2_C(O)), 7.45 (s, 8H, Ar-H).

^13^C NMR (CD_3_OD, δ, ppm): 27.85, 28.09, 28.37, 30.66, 31.64, 34.52, 35.23, 40.36, 42.07, 43.00, 50.89, 54.52, 75.32, 129.79, 136.06, 148.87, 159.26, 170.56, 175.21.

FTIR ATR (ν, cm^−1^): 3286 (NH_2_), 3069 (N-H), 1641 (C(O)NH, Amide I), 1541 (C(O)NH, Amide II), 1265 (C(O)NH, Amide III), 1025 (C_Ph_OCH_2_).

Elemental analysis. C_112_H_192_N_24_O_16_S_4_ C, 59.55; H, 8.57; N, 14.88; S, 5.68; Found: C, 59.34; H, 8.83; N, 14.91; S, 5.64.

ESI, Calculated [M + 3H]^3+^ *m/z* = 753.8028, [M + 4H]^4+^ *m/z* = 565.6039, [M + 5H]^5+^ *m/z* = 452.6846, [M + 6H]^6+^ *m/z* = 377.4050. Found [M + 3H]^3+^ *m/z* = 753.7989, [M + 4H]^4+^ *m/z* = 565.6018, [M + 5H]^5+^ *m/z* = 452.6836, [M + 6H]^6+^ *m/z* = 377.4040.

#### 3.3.2. G1-paco, 5,11,17,23-Tetra-tert-butyl-25,26,27,28-tetrakis[N-(6-(N,N-di(N-(2-aminoethyl)carbamoylethyl)amino)hexyl)carbamoylmethoxy]-2,8,14,20-tetrathiacalix[4]arene

White solid foam, yield 1.45 (96%). M.p. 57 °C

^1^H NMR (CDCl_3_, δ, ppm, *J*/Hz): 1.09 (s, 18H, (CH_3_)_3_C), 1.30–1.72 (m, 32H, C(O)NHCH_2_CH_2_CH_2_CH_2_, C(O)NHCH_2_CH_2_CH_2_CH_2_, C(O)NHCH_2_CH_2_CH_2_CH_2_, CH_2_CH_2_N), 1.35 (s, 18H, (CH_3_)_3_C), 2.36 (m, 16H, NCH_2_CH_2_C(O)NH), 2.45 (m, 8H, CH_2_CH_2_N), 2.66–2.80 (m, 32H, NCH_2_CH_2_C(O)NH, CH_2_NH_2_), 3.14 (m, 2H, C(O)NHCH_2_), 3.24 (m, 16H, C(O)NHCH_2_), 3.28–3.42 (m, 6H, C(O)NHCH_2_), 4.25 (d, 2H, OCH_2_C(O), ^2^*J*_HH_ = 13.7), 4.61 (s, 2H, OCH_2_C(O)), 4.85 (s, 2H, OCH_2_C(O)), 5.00 (d, 2H, OCH_2_C(O), ^2^*J*_HH_ = 13.7), 7.12 (d, 2H, Ar-H, ^4^*J*_HH_ = 2.3), 7.61 (d, 2H, Ar-H, ^4^*J*_HH_ = 2.3), 7.66 (s, 2H, Ar-H), 7.84 (s, 2H, Ar-H).

^13^C NMR (CD_3_OD, δ, ppm): 27.82, 27.85, 28.00, 28.08, 28.11, 28.27, 28.37, 28.43, 30.52, 30.65, 30.68, 31.69, 31.78, 31.87, 34.48, 35.15, 35.19, 35.28, 39.97, 40.34, 40.40, 42.05, 43.01, 50.83, 50.86, 54.46, 54.51, 54.60, 70.69, 74.04, 74.55, 127.99, 128.06, 129.45, 130.01, 134.71, 135.52, 135.92, 137.46, 147.14, 147.58, 148.84, 157.87, 159.35, 160.86, 170.13, 170.50, 170.84, 175.14, 175.24.

FTIR ATR (ν, cm^−1^): 3286 (NH_2_), 3068 (N-H), 1642 (C(O)NH, Amide I), 1540 (C(O)NH, Amide II), 1265 (C(O)NH, Amide III), 1025 (C_Ph_OCH_2_).

Elemental analysis. C_112_H_192_N_24_O_16_S_4_ C, 59.55; H, 8.57; N, 14.88; S, 5.68; Found: C, 59.50; H, 8.62; N, 14.79; S, 5.57.

ESI, Calculated [M + 3H]^3+^ *m/z* = 753.8028, [M + 4H]^4+^ *m/z* = 565.6039, [M + 5H]^5+^ *m/z* = 452.6846, [M + 6H]^6+^ *m/z* = 377.4050. Found [M + 3H]^3+^ *m/z* = 753.7994, [M + 4H]^4+^ *m/z* = 565.6018, [M + 5H]^5+^ *m/z* = 452.6843, [M + 6H]^6+^ *m/z* = 377.4045.

#### 3.3.3. G1-1,3-alt, 5,11,17,23-Tetra-tert-butyl-25,26,27,28-tetrakis[N-(6-(N,N-di(N-(2-aminoethyl)carbamoylethyl)amino)hexyl)carbamoylmethoxy]-2,8,14,20-tetrathiacalix[4]arene

White solid foam, yield 1.49 (99%). M.p. 59–60 °C

^1^H NMR (CD_3_OD, δ, ppm, *J*/Hz): 1.28 (s, 36H, (CH_3_)_3_C), 1.33 (m, 16H, C(O)NHCH_2_CH_2_CH_2_CH_2_, C(O)NHCH_2_CH_2_CH_2_CH_2_), 1.42–1.63 (m, 16H, CH_2_CH_2_NH_2_, C(O)NHCH_2_CH_2_CH_2_CH_2_), 2.37 (m, 16H, NCH_2_CH_2_C(O)NH), 2.46 (m, 8H, CH_2_CH_2_N), 2.64–2.82 (m, 32H, CH_2_NH_2_, NCH_2_CH_2_C(O)NH), 3.12–3.29 (m, 24H, C(O)NHCH_2_), 4.15 (s, 8H, OCH_2_C(O)), 7.59 (s, 8H, Ar-H).

^13^C NMR (CD_3_OD, δ, ppm): 27.82, 28.15, 28.33, 30.71, 31.72, 34.50, 35.34, 40.73, 42.05, 42.99, 50.85, 54.53, 71.44, 129.17, 133.04, 148.91, 157.80, 169.91, 175.23.

FTIR ATR (ν, cm^−1^): 3286 (NH_2_), 3060 (N-H), 1641 (C(O)NH, Amide I), 1535 (C(O)NH, Amide II), 1265 (C(O)NH, Amide III), 1026 (C_Ph_OCH_2_).

Elemental analysis. C_112_H_192_N_24_O_16_S_4_ C, 59.55; H, 8.57; N, 14.88; S, 5.68; Found: C, 59.82; H, 8.38; N, 14.53; S, 5.62.

ESI, Calculated [M + 3H]^3+^ *m/z* = 753.8028, [M + 4H]^4+^ *m/z* = 565.6039, [M + 5H]^5+^ *m/z* = 452.6846, [M + 6H]^6+^ *m/z* = 377.4050. Found [M + 3H]^3+^ *m/z* = 753.7992, [M + 4H]^4+^ *m/z* = 565.6017, [M + 5H]^5+^ *m/z* = 452.6835, [M + 6H]^6+^ *m/z* = 377.4043.

### 3.4. Preparation of the Compounds G1-HCl

All compounds **G1** were converted into the ammonium form. For the preparation of the 1 mM solution of **G1-HCl**, 5µmol of the **G1** were dissolved in 240 µL of HCl (0.25 M), after that buffer solution (10 mM Tris-HCl, pH 7.4) was added up to 5 mL.

### 3.5. UV-Visible Spectroscopy

UV-Visible spectra were recorded on the Shimadzu UV-3600 spectrophotometer (Kyoto, Japan) using 1 cm quartz cuvette at 293 K. Salmon sperm DNA (Sigma) was used as received. The purity of the DNA was checked by the ratio of the absorbance A260/A280 > 1.8, indicating the DNA was sufficiently free from protein. Concentration of DNA was 0.0128 mg/mL (1.855 × 10^−5^ M base pairs). Concentrations of **G1-HCl** were 8.33 × 10^−7^ M, 3.33 × 10^−5^ M, and 3.33 × 10^−6^ M. UV-Vis spectra were registered in 10 mM Tris-HCl buffer (pH = 7.4) in 30 min after preparation of the solutions.

### 3.6. Study of DNA Stability in the Presence of G1-HCl Compounds by UV-Visible Spectroscopy

The concentration of the DNA was 0.0256 mg/mL (3.710 × 10^−5^ M base pairs), that of **G1-HCl** 3.33 × 10^−5^ M. Solutions of pure salmon sperm DNA and that with **G1-HCl** were prepared in 10 mM Tris-HCl buffer (pH = 7.4). UV-Vis spectra were recorded at 293 K right after preparation and then after 1, 2, 3, and 7 days. The solutions were stored at 293 K between the measurements.

### 3.7. Fluorescence Spectroscopy

Fluorescence spectra were recorded on the Fluorolog 3 luminescent spectrometer (Horiba Jobin Yvon, Longjumeau, France). The excitation wavelength was selected as 350 nm. The emission scan range was 370–650 nm. Excitation and emission slits were 1 nm. Quartz cuvettes with an optical path length of 10 mm were used. The cuvette was placed at the front face position to avoid the inner filter effect. Fluorescence spectra were automatically corrected by the Fluorescence program. Spectra were recorded at 293 K in 10 mM Tris-HCl buffer (pH = 7.4). The concentration of the 4′,6-diamidino-2-phenylindole (DAPI) hydrochloride was 10 µM. The concentration of the base pairs of salmon sperm DNA was 5.565 × 10^−5^ M (0.0384 mg/mL). Concentrations of the PAMAM-calix-dendrimers **G1-HCl** were 1 × 10^−4^ M and 1 × 10^−5^ M.

### 3.8. Dynamic Light Scattering (DLS) Determination of the Hydrodynamic Size of the Particles

The particle size was determined by the Zetasizer Nano ZS instrument (Malvern Instruments, Worcestershire, UK) at 293 K. The instrument contains the 4 mW He-Ne laser operating at the wavelength of 633 nm and incorporates noninvasive backscatter optics (NIBS). The measurements were performed at the detection angle of 173°, and the measurement position within the quartz cuvette was automatically determined by the software. The results were processed with the DTS (Dispersion Technology Software 4.20) software package. Deionized water with resistivity > 18.0 MΩ cm (Millipore-Q) was used for the preparation of the solutions. The experiments were carried out in 10 mM Tris-HCl buffer (pH = 7.4). During the experiments, the DNA concentration (0.0384 mg/mL, 5.565 × 10^−5^ M base pairs) remained constant while the concentration of the receptors was varied from 5 × 10^−6^ to 5 × 10^−4^ M. The determination of the particle size was carried out in one hour after the sample preparation. To assess the kinetic stability of the systems, the measurements were also carried out under similar conditions after 3 and 5 h.

### 3.9. Electrokinetic Potentials

Electrokinetic (ζ) potentials were determined by electrophoretic light scattering on the Zetasizer Nano ZS (Malvern Instruments, Worcestershire, UK). Samples were prepared for the DLS measurements and transferred with the syringe to the disposable folded capillary cell for measurement. The ζ potentials were measured using the Malvern M3-PALS method and averaged from five measurements.

### 3.10. Circular Dichroism (CD) Studies

Changes in the intensity of the CD signal of the DNA, alone and in the presence of the compounds **G1**, were recorded from 223 nm to 323 nm at 293 K using the Jasco J-1500 spectropolarimeter (Tokyo, Japan) in the quartz cuvette with the 10 mm optical path length. The experiment was carried out in 10 mM Tris-HCl buffer (pH = 7.4). The concentration of the DNA was 0.0128 mg/mL (1.855 × 10^−5^ M base pairs); those of **G1-HCl** 1.0 × 10^−6^, 3.3 × 10^−6^, 33 × 10^−6^ M.

### 3.11. Transmission Electron Microscopy (TEM)

TEM analysis was carried with the Hitachi HT7700 Exalens microscope (Tokyo, Japan). The DNA concentration was 0.0384 mg/mL (5.565 × 10^−5^ M base pairs), the concentration of **G1-cone** was 5×10^−5^ M. The recording of the images of the mixture of DNA with thiacalixarene was carried out in 1 h after mixing the solutions at 293 K.

## 4. Conclusions

A convenient method for the synthesis of first-generation PAMAM dendrimers based on thiacalix[4]arene has been developed for the first time. Novel symmetric and asymmetric PAMAM-calix-dendrimers with the macrocyclic core in three conformations (*cone*, *partial cone*, and *1,3-alternate*) were obtained with high yields (96–99%). The interaction of the ammonium form of the obtained compounds with salmon sperm DNA with the formation of associates with the size of up to 200 nm is shown by UV-Vis spectroscopy, DLS, and TEM. It was found that binding is carried out by electrostatic interactions with the phosphate groups of the biopolymer, as well as at the DNA groove. It was shown by the CD method that the structure of the DNA does not undergo significant changes upon binding. The PAMAM-calix-dendrimer based on the macrocycle in *cone* conformation has stabilizing effect on the DNA and prevents its degradation. This effect is explained by the amphiphilicity of the **G1-cone-HCl** structure, while the binding is accompanied by the isolation of the DNA molecules from the aqueous medium. The results obtained open up high prospects for practical use in the development of delivery systems, stabilization, and storage of DNA.

## Figures and Tables

**Table 1:** Size distribution (intensity) and values of ζ-potential of **G1-HCl** with salmon sperm DNA (5.565 × 10^−5^ M) in 10 mM Tris-HCl buffer, pH 7.4.

Compound	G1 Concentration, M	d, nm (PDI)	ζ-Potential, mV
**G1-1,3-alt-HCl**	5 × 10^−4^	115.6 ± 1.4 (0.167 ± 0.011)	44.9 ± 2.6
	1 × 10^−4^	169.4 ± 1.1 (0.099 ± 0.009)	30.8 ± 1.1
	5 × 10^−5^	265.2 ± 5.2 (0.162 ± 0.013)	21.1 ± 0.7
	1 × 10^−5^	204.3 ± 2.5 (0.250 ± 0.009)	−33.0 ± 1.2
	5 × 10^−6^	149.4 ± 1.4 (0.197 ± 0.010)	−44.6 ± 2.9
**G1-cone-HCl**	5 × 10^−4^	122.9 ± 0.9 (0.181 ± 0.007)	49.0 ± 1.7
	1 × 10^−4^	162.4 ± 1.2 (0.176 ± 0.014)	44.8 ± 2.9
	5 × 10^−5^	170.4 ± 0.6 (0.188 ± 0.011)	33.5 ± 1.1
	1 × 10^−5^	196.3 ± 3.2 (0.254 ± 0.007)	−28.0 ± 2.4
	5 × 10^−6^	129.8 ± 1.1 (0.178 ± 0.012)	−37.7 ± 4.3
**G1-paco-HCl**	5 × 10^−4^	112.2 ± 0.4 (0.155 ± 0.007)	45.2 ± 2.2
	1 × 10^−4^	159.8 ± 0.8 (0.118 ± 0.016)	36.9 ± 1.6
	5 × 10^−5^	196.1 ± 1.9 (0.099 ± 0.014)	25.7 ± 0.7
	1 × 10^−5^	172.7 ± 1.0 (0.107 ± 0.020)	−30.7 ± 2.3
	5 × 10^−6^	152.6 ± 1.8 (0.174 ± 0.011)	−34.4 ± 1.3

## Data Availability

The data presented in this study are available in Supplementary Materials.

## Supplementary Materials

The following are available online at https://www.mdpi.com/article/10.3390/ijms222111901/s1, Figures S1–S24: NMR, IR and mass spectra of synthesized compounds; Figures S25–S34: UV-Vis spectra; Figures S35–S37: Fluorescence spectra; Figure S38: CD spectra; Figures S39–S69: DLS data; Figures S70–S71: TEM images; Table S1: Size distributions (by intensity) of G1-HCl aggregates (10 mM Tris-HCl, pH 7.4).
