# Supplementary material for: PAMAM-calix-dendrimers: Synthesis and Thiacalixarene Conformation Effect on DNA Binding
Source: Int J Mol Sci. 2021 Nov 2;22(21):11901. doi: 10.3390/ijms222111901 (PMC8585033; doi:10.3390/ijms222111901)
Supplement: Supplementary file 1 [file ijms-22-11901-s001.zip › ijms-1446935-supplementary.pdf]

Electronic Supplementary Information

PAMAM-calix-dendrimers: Synthesis and Thiacalixarene Conformation Effect on DNA Binding

Olga Mostovaya [1], Pavel Padnya [1,*], Igor Shiabiev [1], Timur Mukhametzyanov [1] and Ivan Stoikov [1,*]

1. NMR, IR and mass spectra of synthesized compounds 2

2. Complexation investigation 14

2.1 UV-Vis spectra 14

2.2 Fluorescence spectra 20

2.3 CD spectra 24

2.4 DLS data 25

2.5 TEM images 36

1. NMR, IR and mass spectra of synthesized compounds

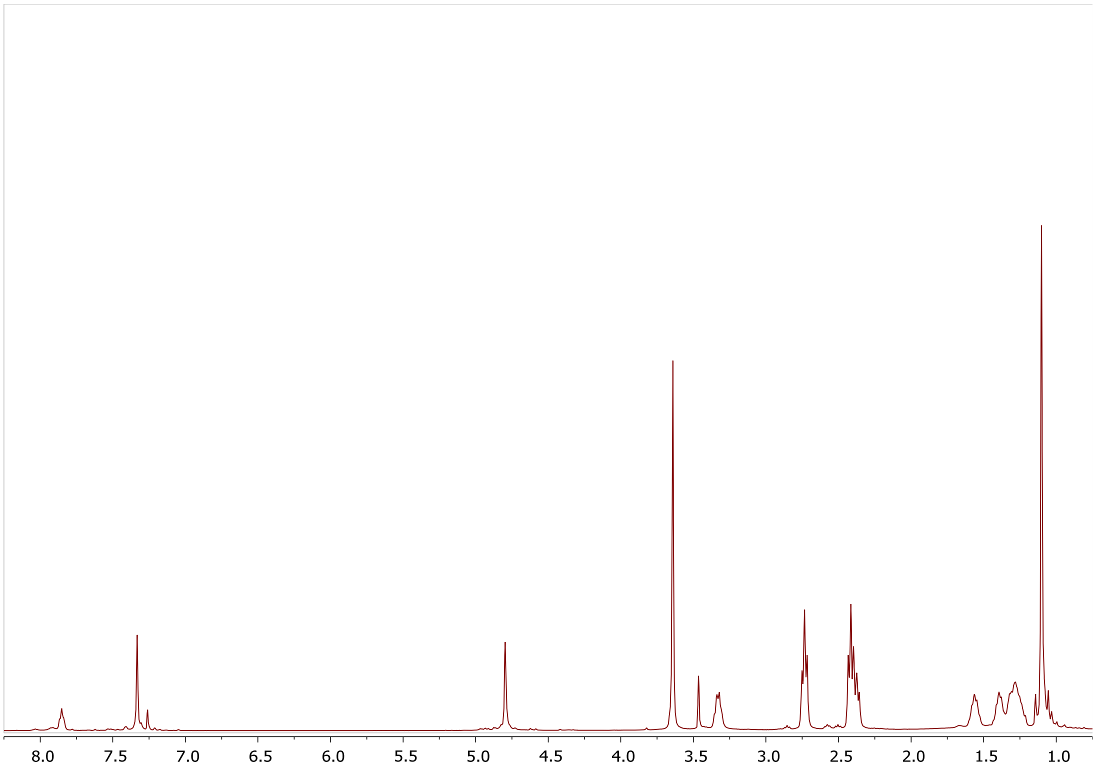

Fig. S1. ^{1}H NMR spectrum of **G0.5-cone**, $CDCl_3$, 298 K, 400 MHz

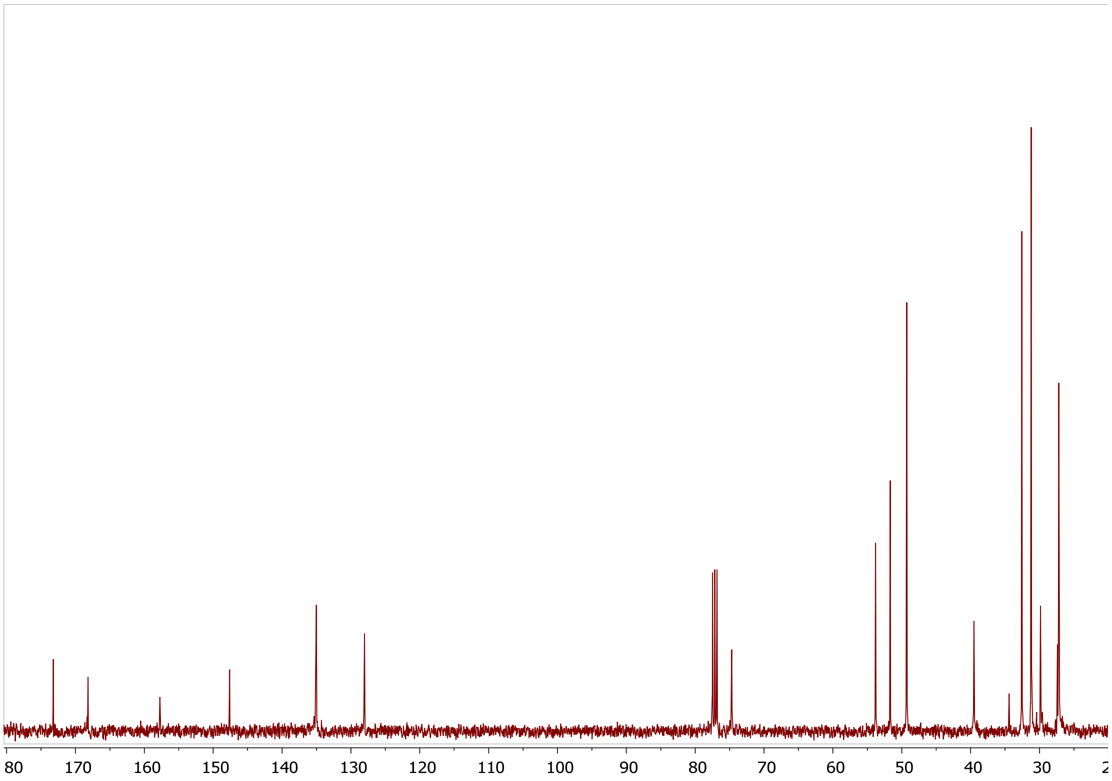

Fig. S2. $^{13}C\{^{1}H\}$ NMR spectrum of **G0.5-cone**, $CDCl_3$, 298 K, 100 MHz

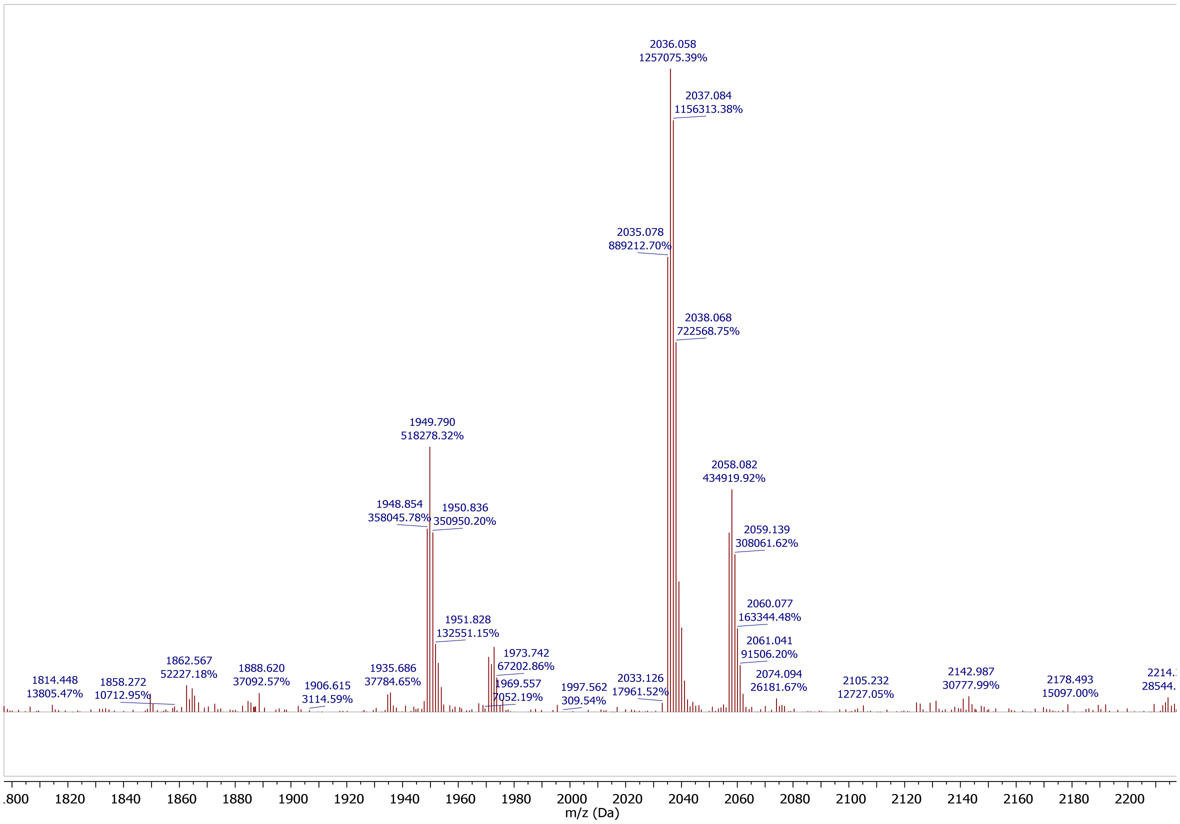

Fig. S3. Mass spectrum (MALDI TOF, 4-nitroaniline matrix) of **G0.5-cone**

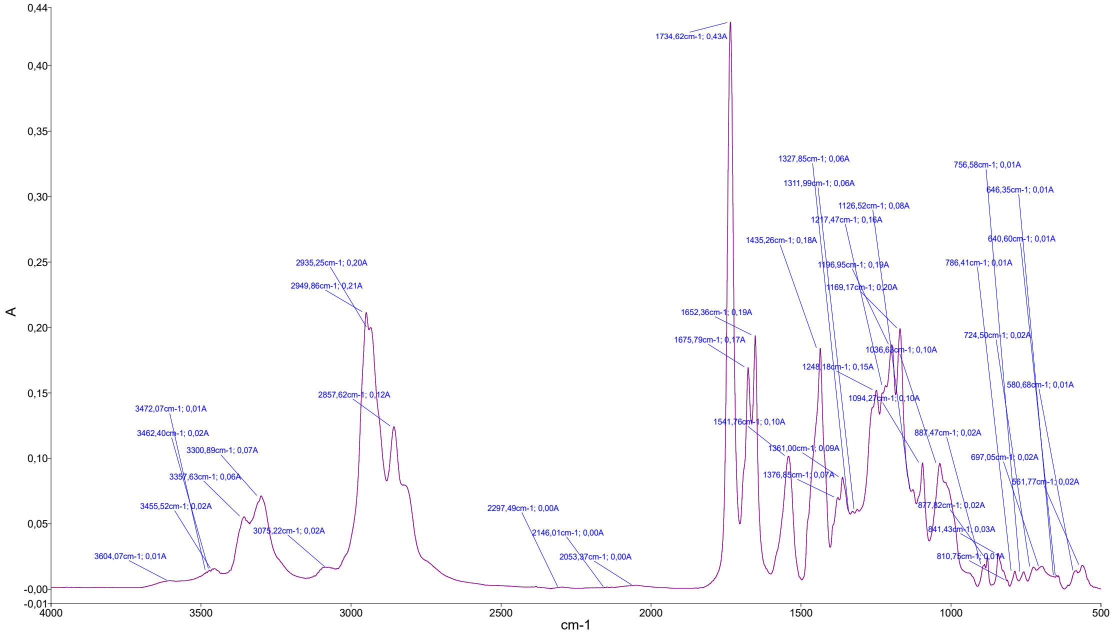

Fig. S4. FTIR-ATR spectrum of **G0.5-cone**

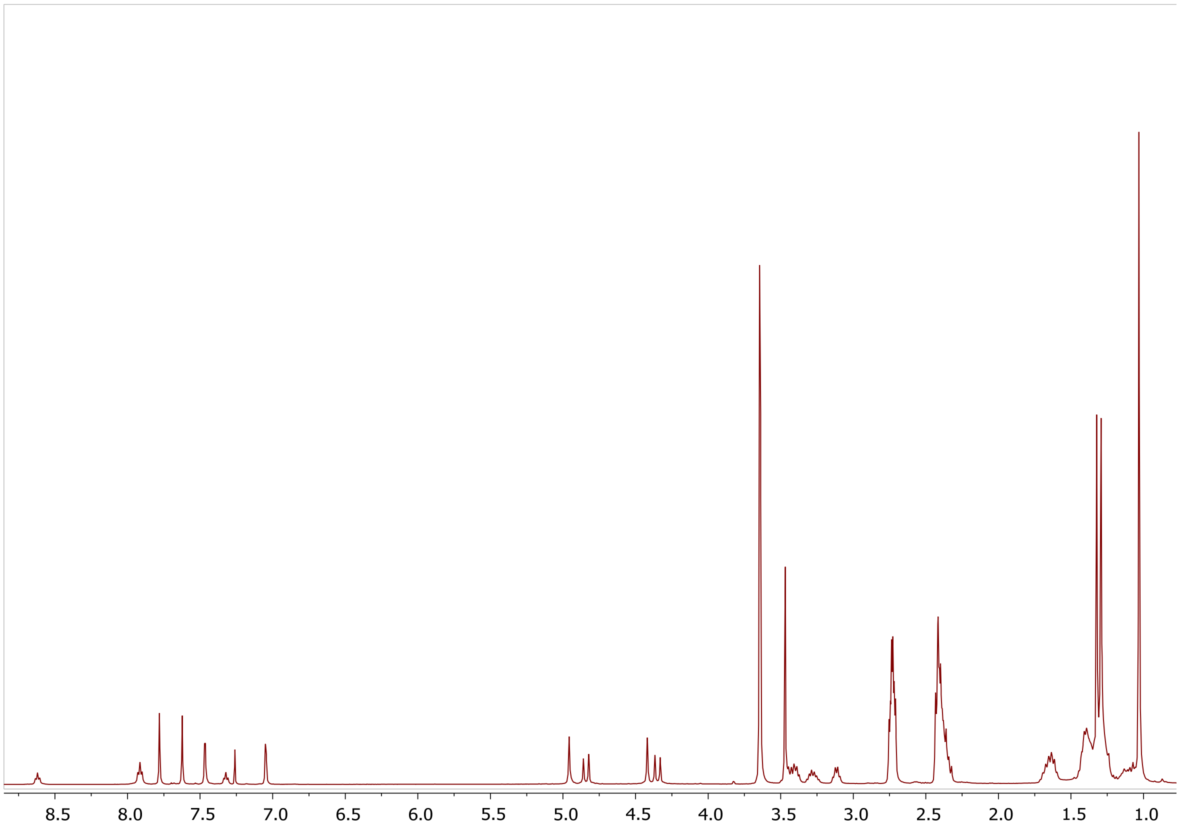

Fig. S5. ^{1}H NMR spectrum of **G0.5-paco**, $CDCl_3$, 298 K, 400 MHz

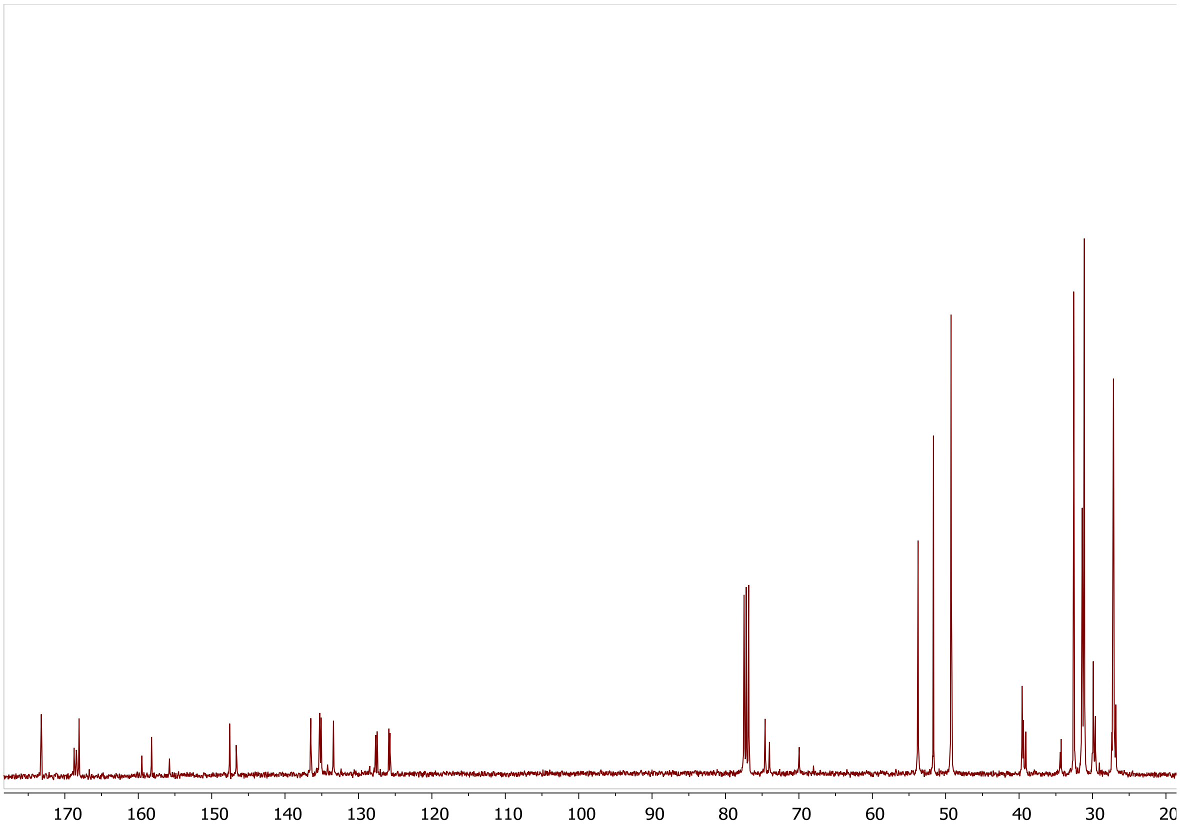

Fig. S6. $^{13}C\{^{1}H\}$ NMR spectrum of **G0.5-paco**, $CDCl_3$, 298 K, 100 MHz

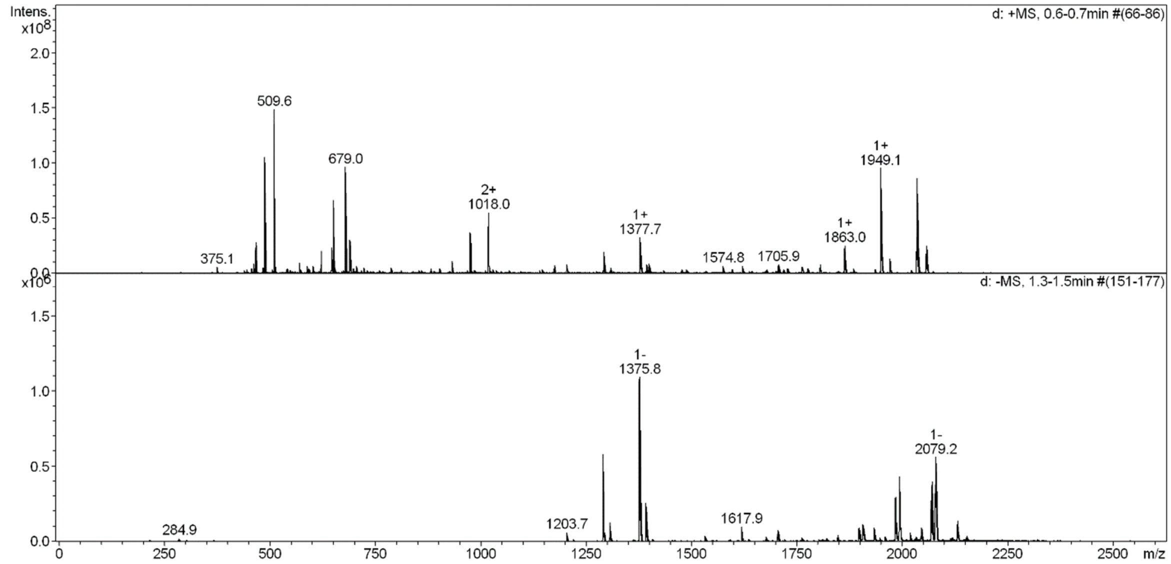

Fig. S7. Mass spectrum (ESI) of **G0.5-paco**

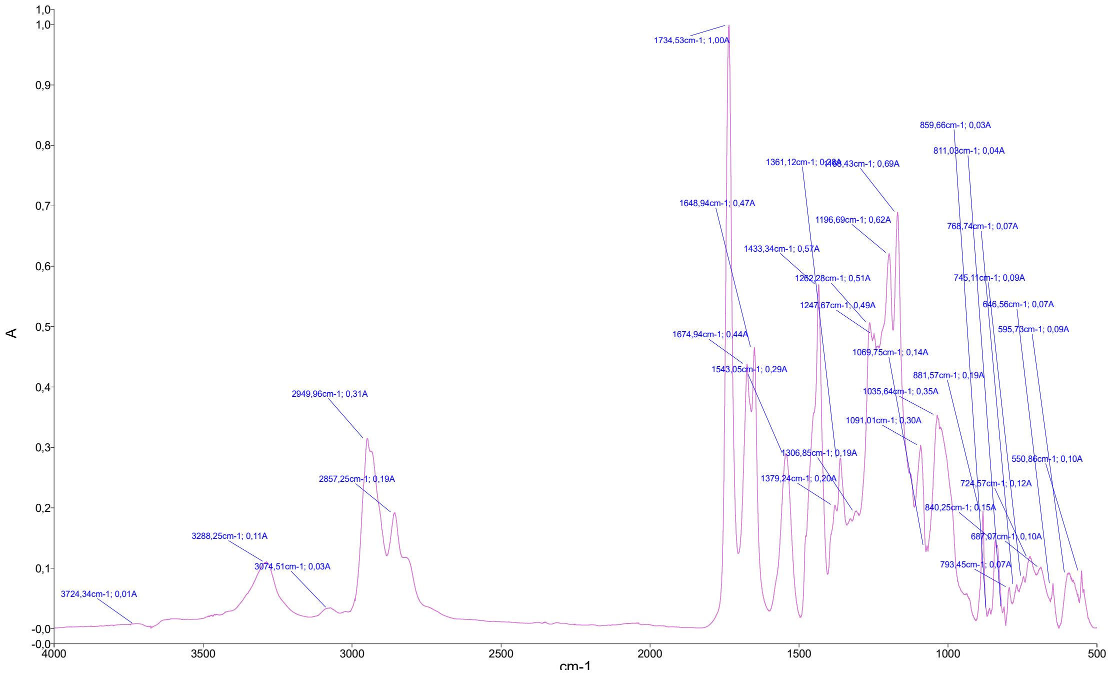

Fig. S8. FTIR-ATR spectrum of **G0.5-paco**

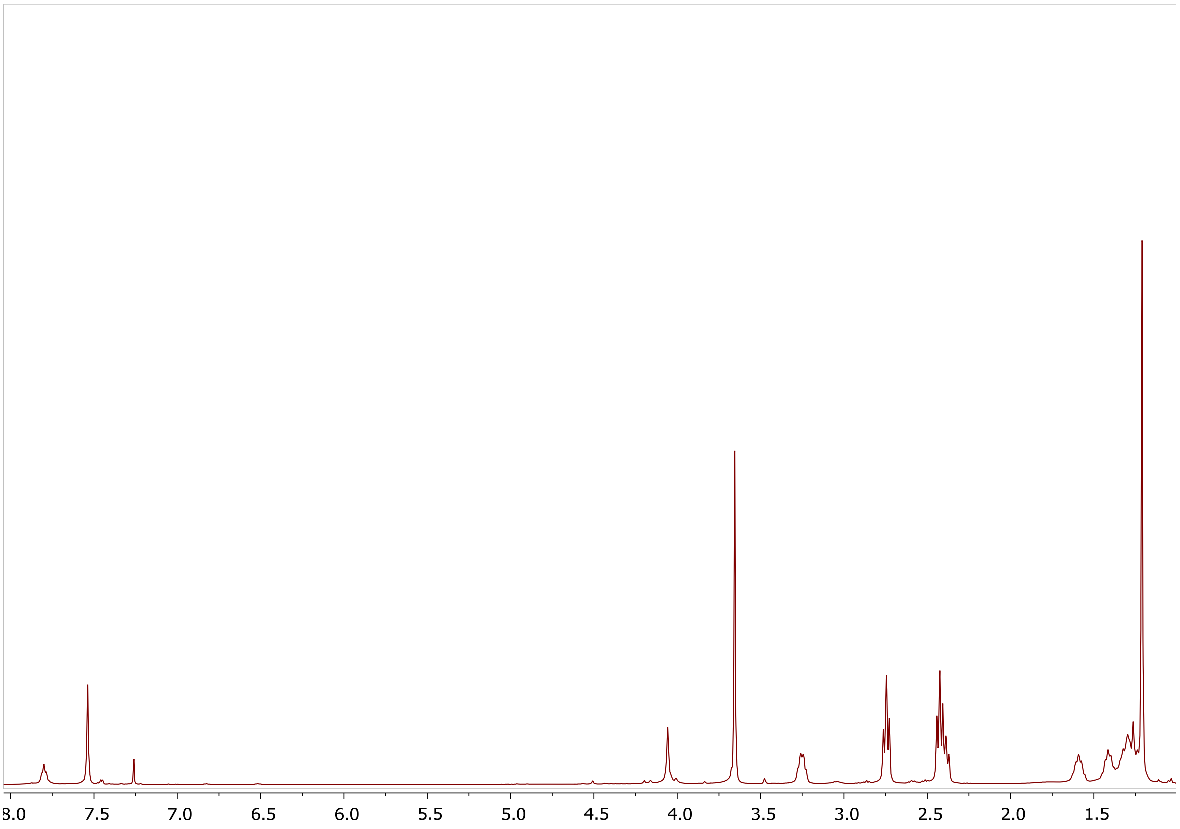

Fig. S9. ^{1}H NMR spectrum of **G0.5-alt**, $CDCl_3$, 298 K, 400 MHz

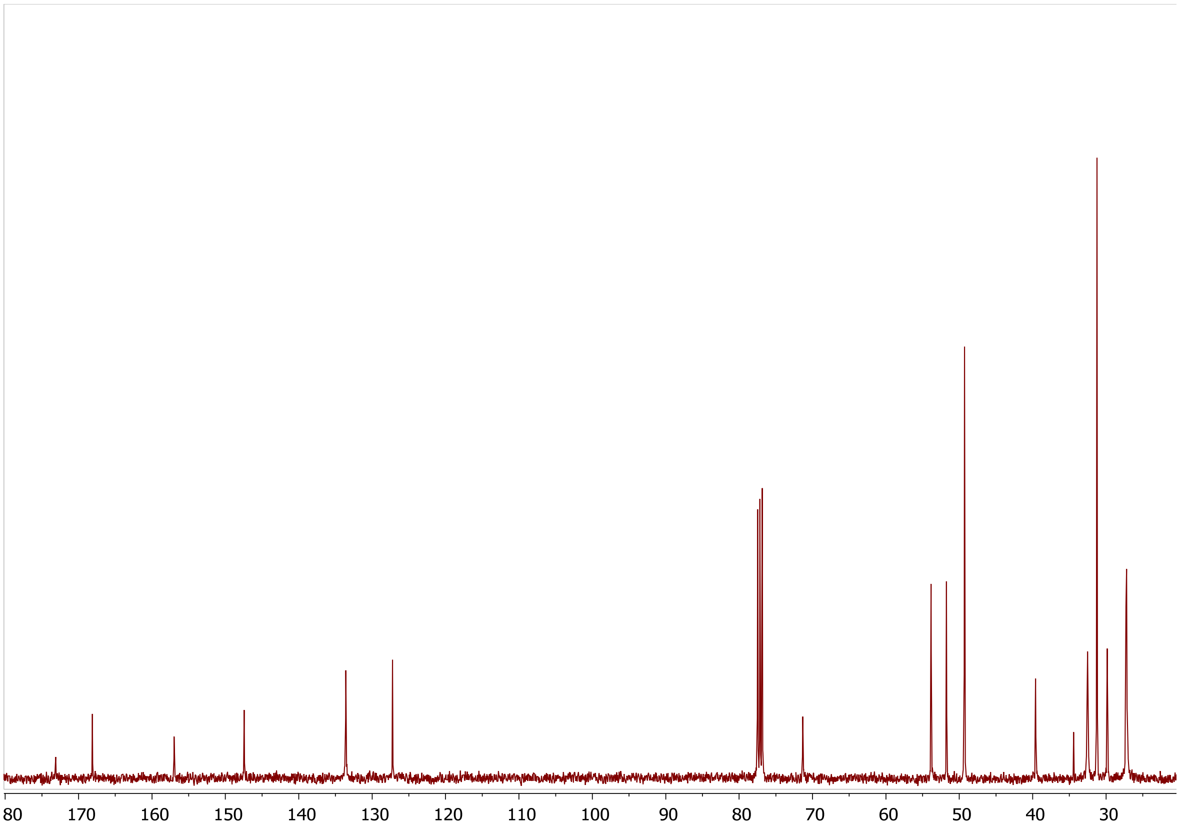

Fig. S10. $^{13}C\{^{1}H\}$ NMR spectrum of **G0.5-alt**, $CDCl_3$, 298 K, 100 MHz

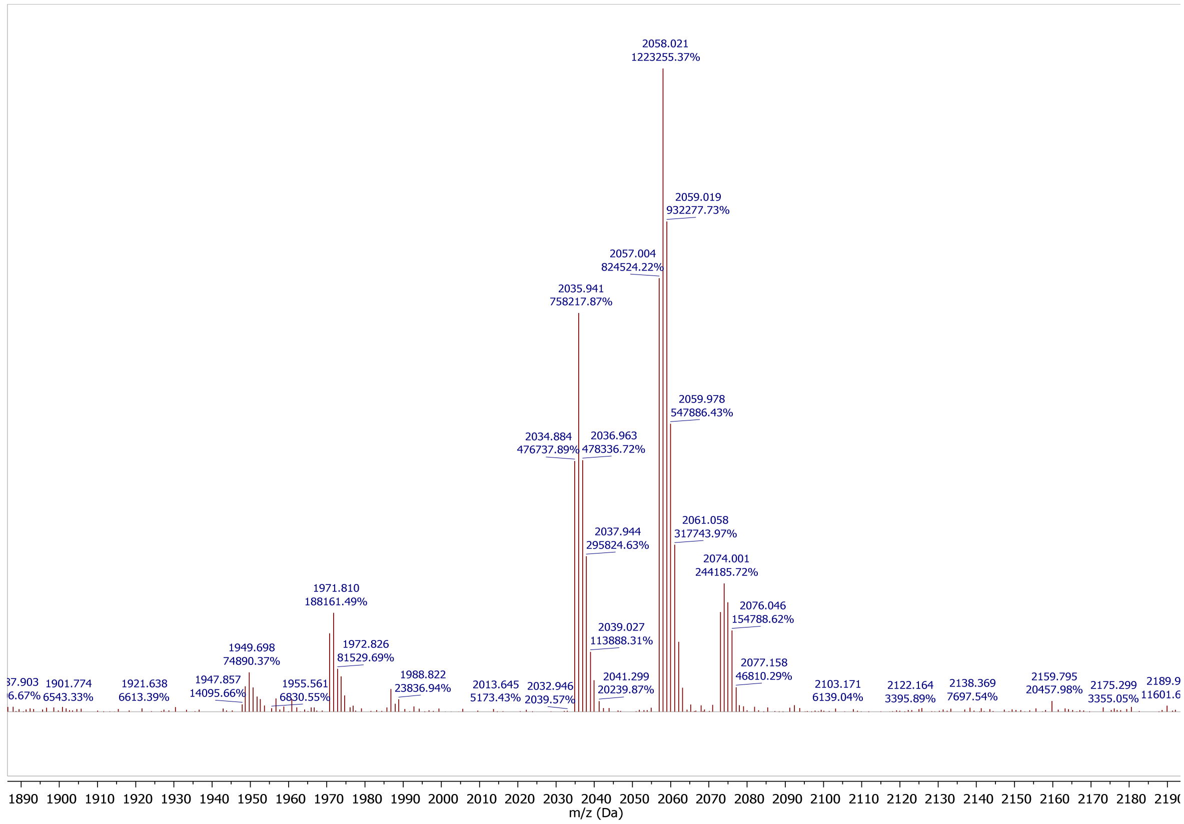

Fig. S11. Mass spectrum (MALDI TOF, 4-nitroaniline matrix) of **G0.5-alt**

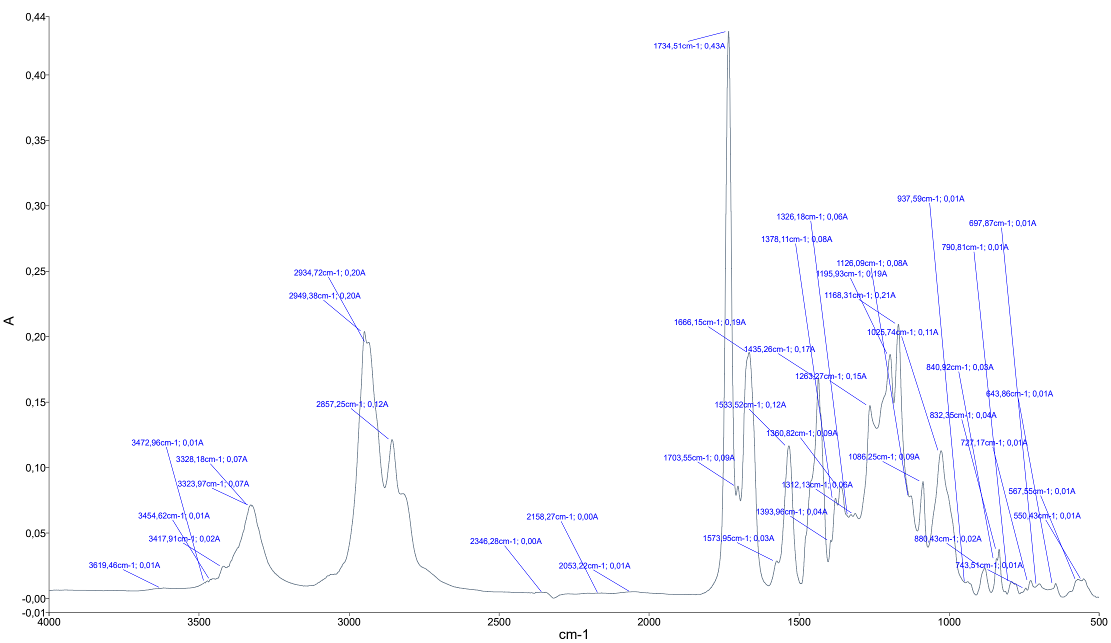

Fig. S12. FTIR-ATR spectrum of **G0.5-alt**

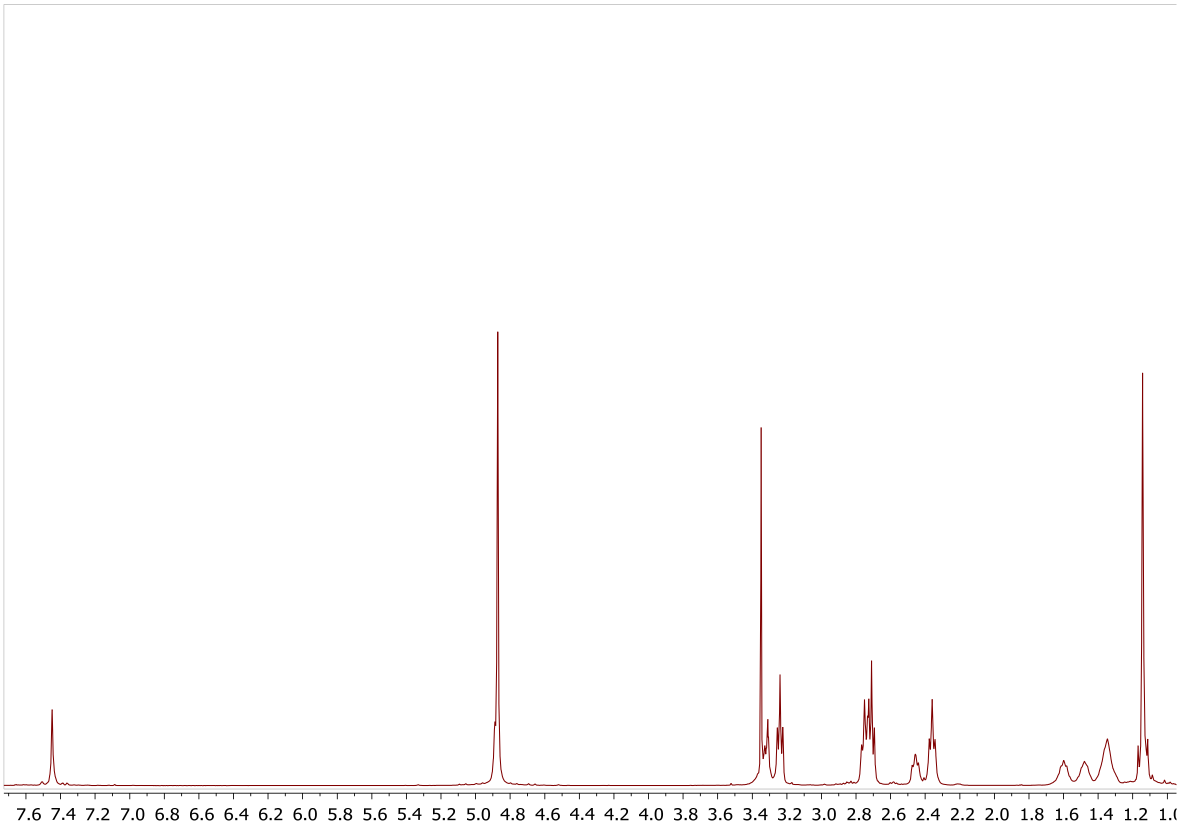

Fig. S13. ^{1}H NMR spectrum of **G1-cone**, CD_3OD, 298 K, 400 MHz

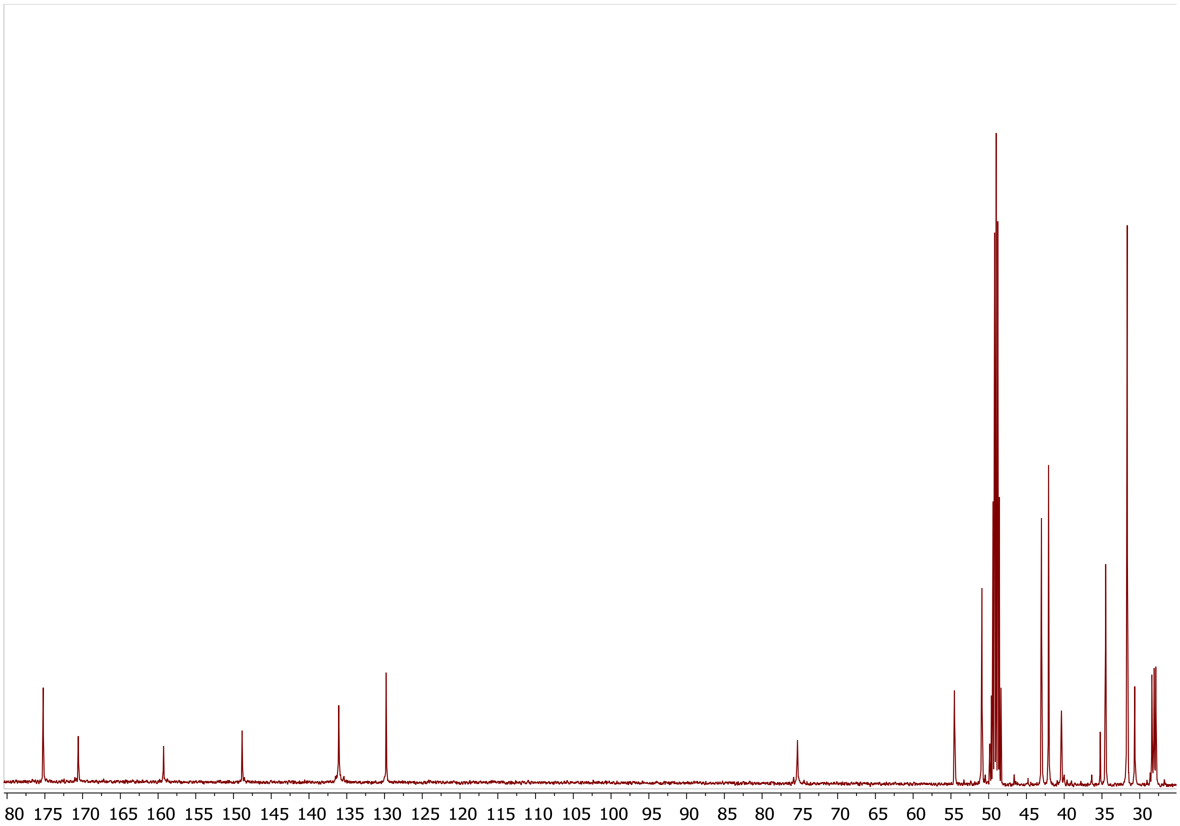

Fig. S14. $^{13}C\{^{1}H\}$ NMR spectrum of **G1-cone**, CD_3OD, 298 K, 100 MHz

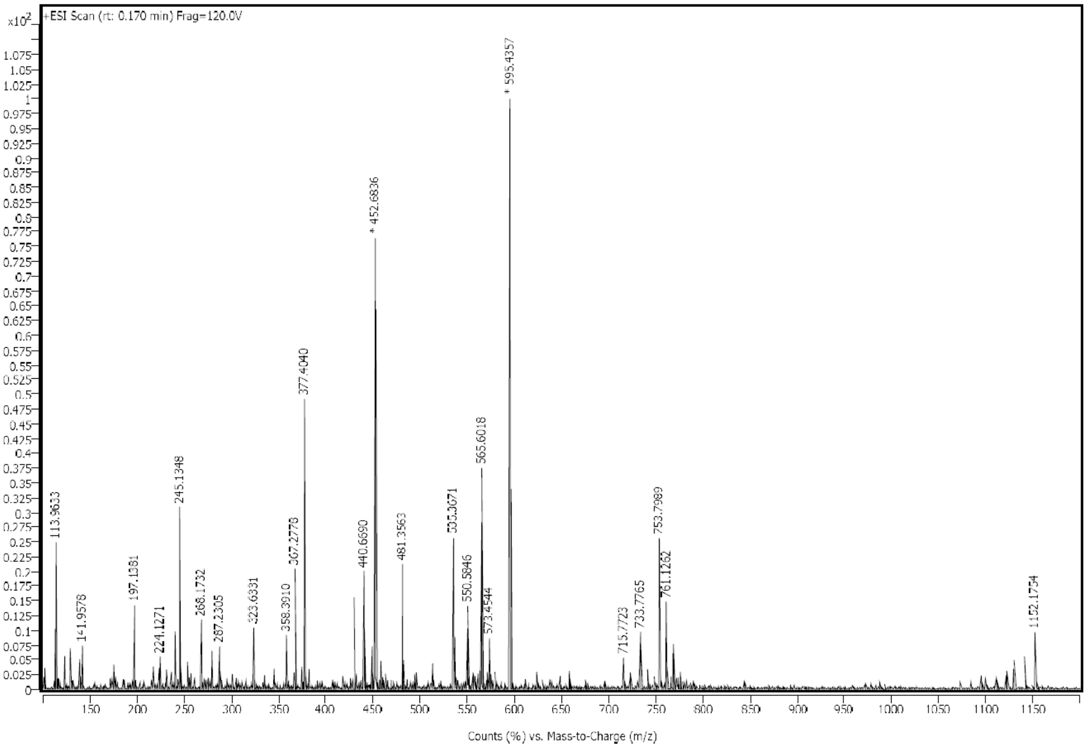

Fig. S15. Mass spectrum (HRESI) of **G1-cone**

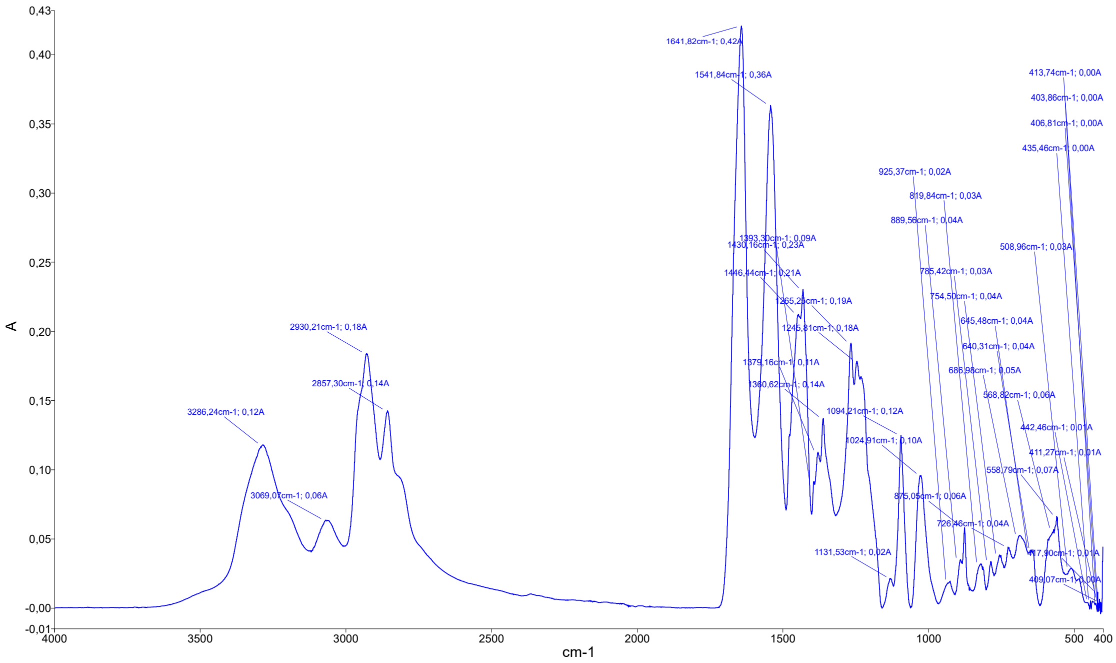

Fig. S16. FTIR-ATR spectrum of **G1-cone**

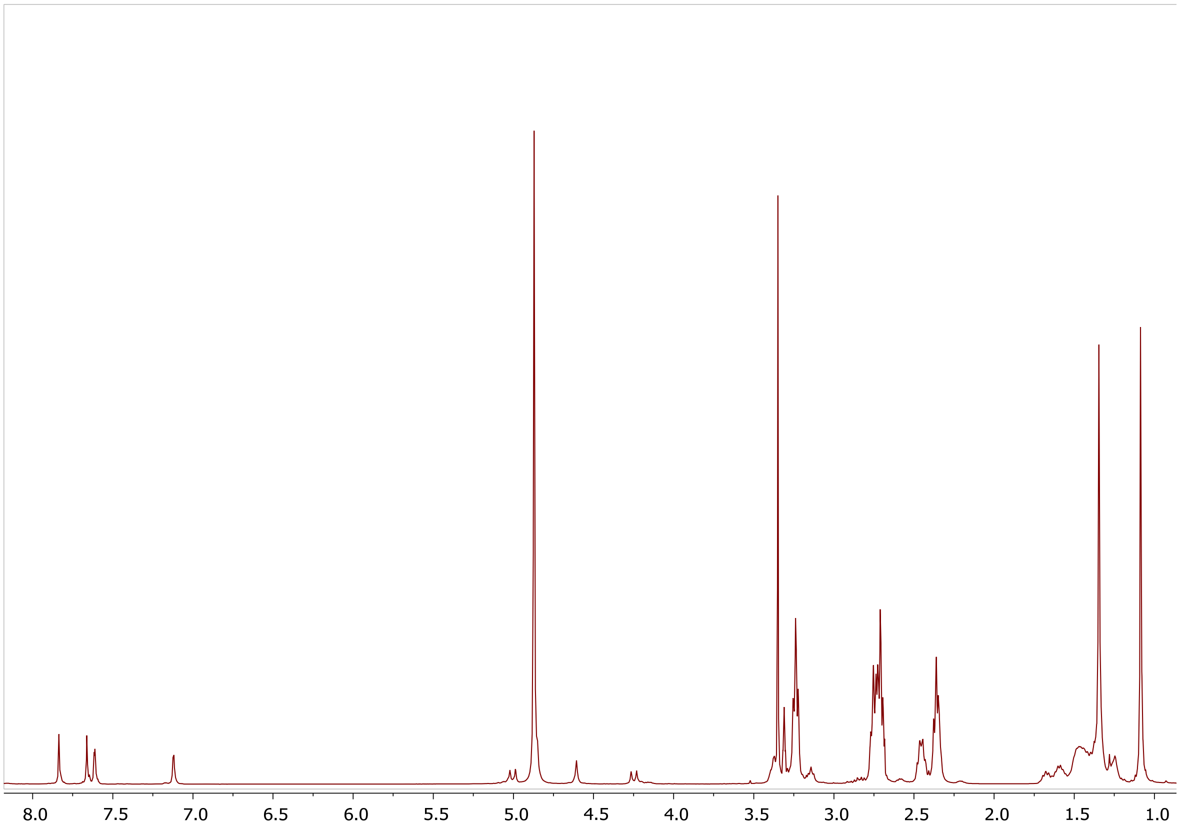

Fig. S17. ^{1}H NMR spectrum of **G1-paco**, CD_3OD, 298 K, 400 MHz

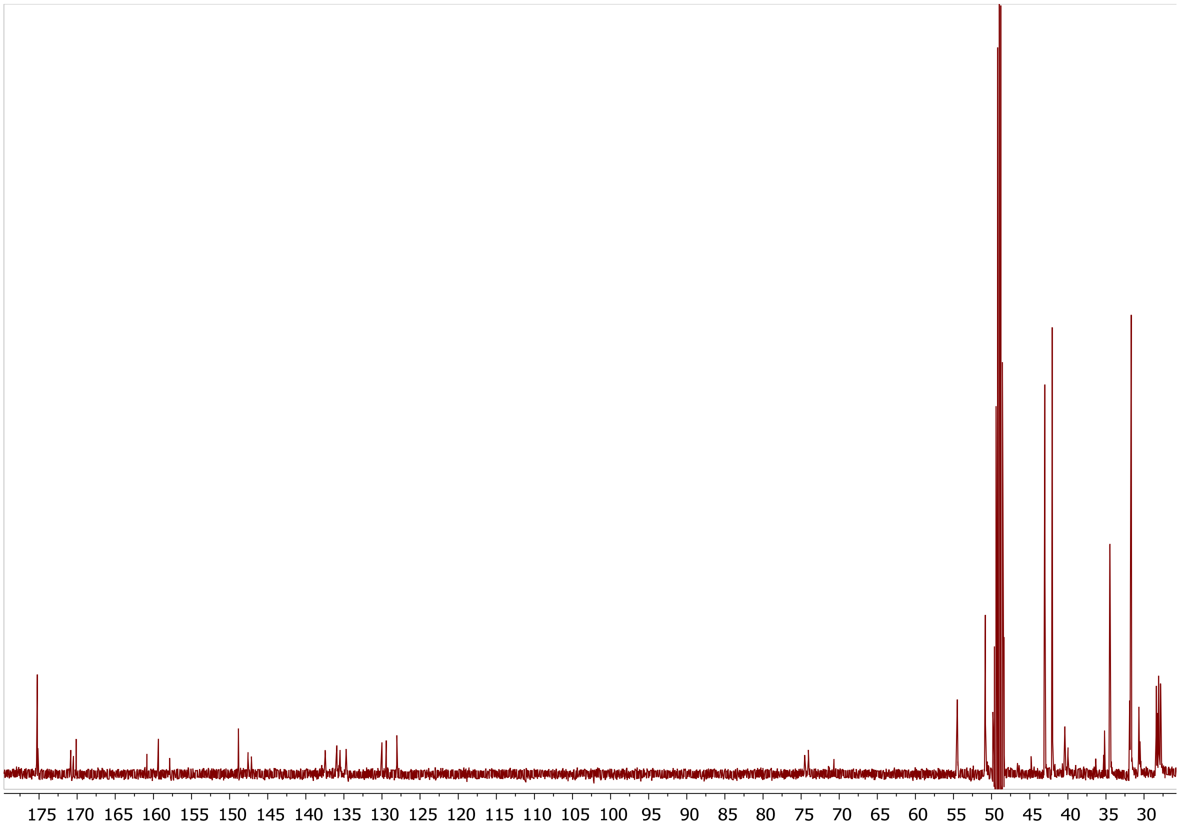

Fig. S18. $^{13}C\{^{1}H\}$ NMR spectrum of **G1-paco**, CD_3OD, 298 K, 100 MHz

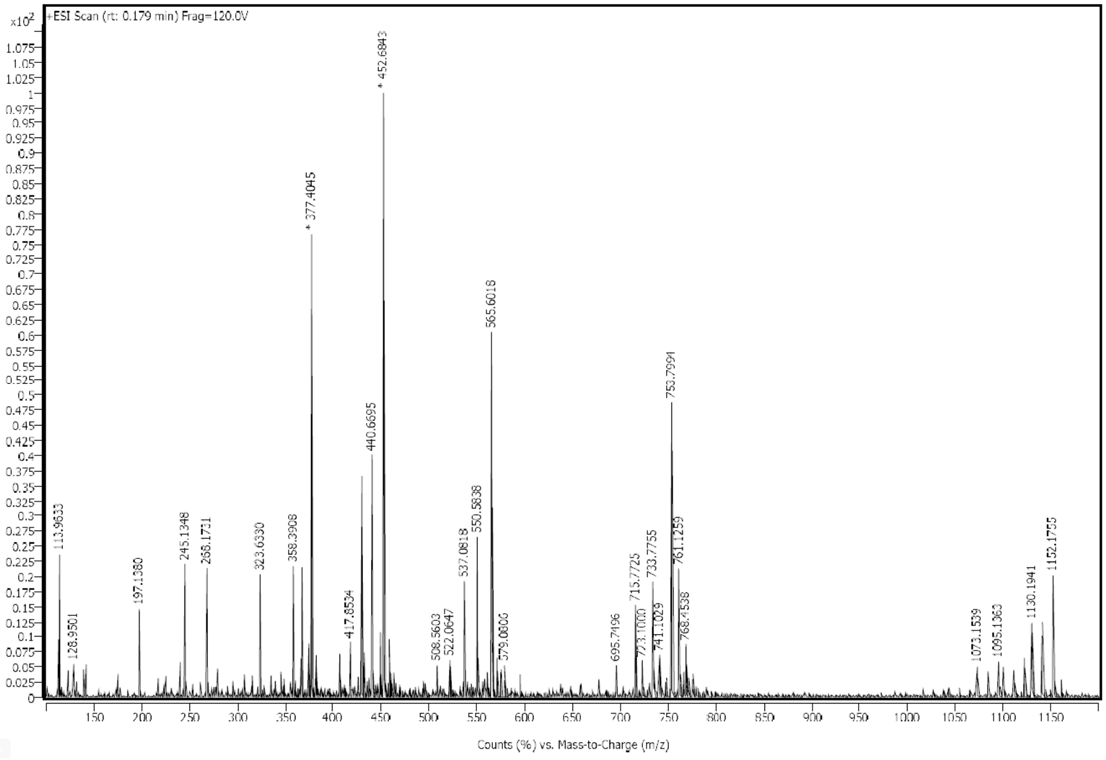

Fig. S19. Mass spectrum (HRESI) of **G1-paco**

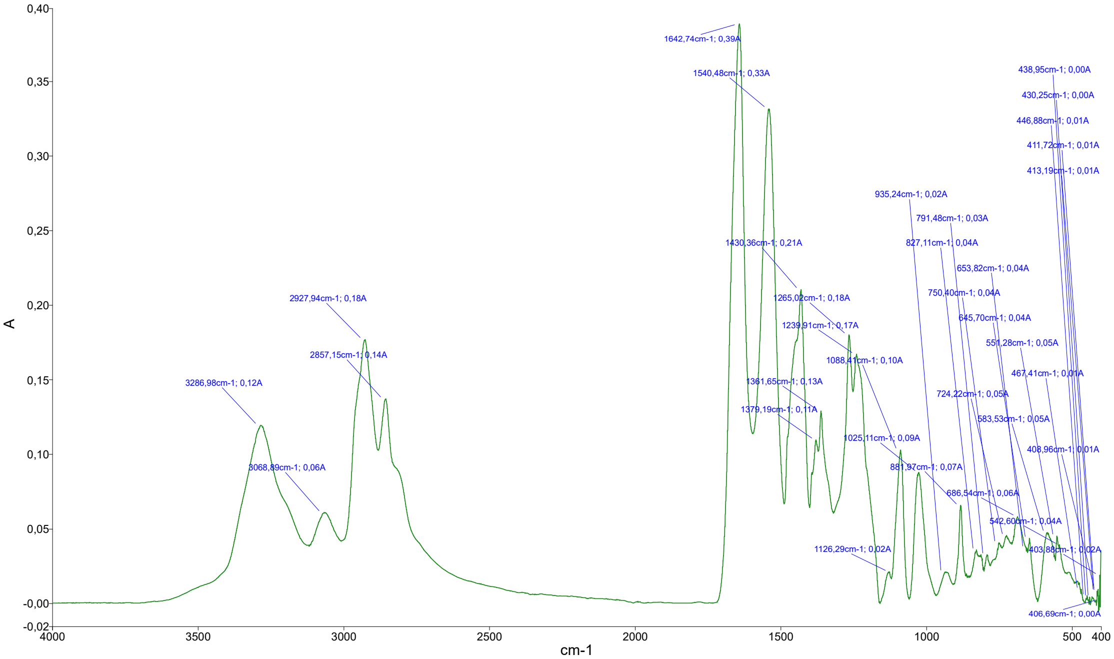

Fig. S20. FTIR-ATR spectrum of **G1-paco**

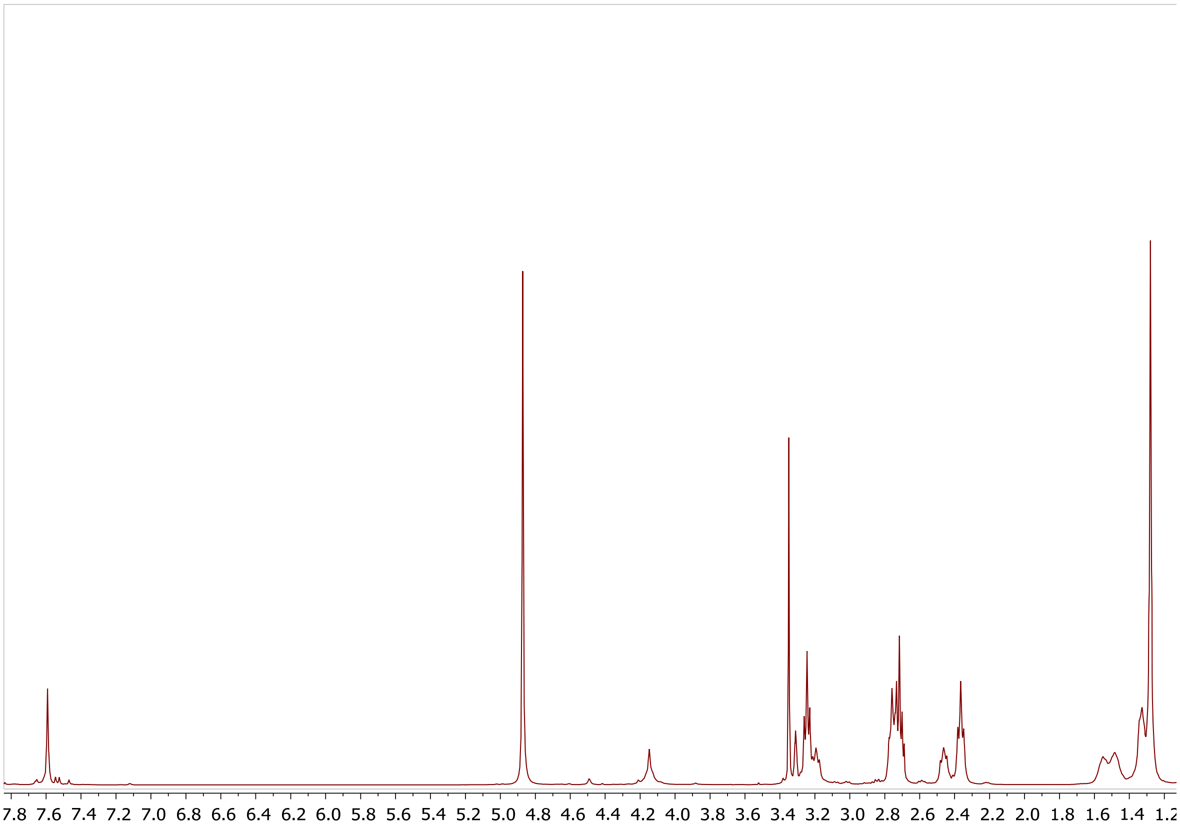

Fig. S21. ^{1}H NMR spectrum of **G1-1,3-alt**, CD_3OD, 298 K, 400 MHz

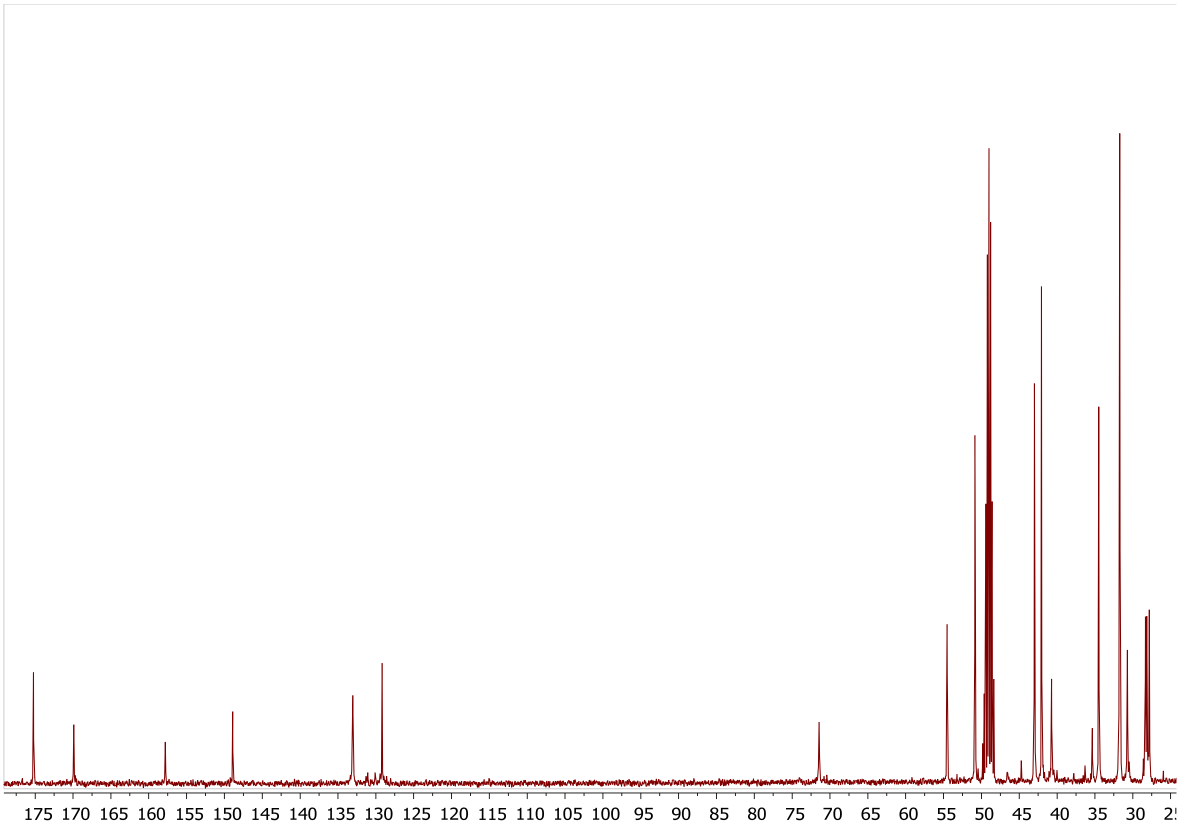

Fig. S22. ^{13}C{^{1}H} NMR spectrum of **G1-1,3-alt**, CD_3OD, 298 K, 100 MHz

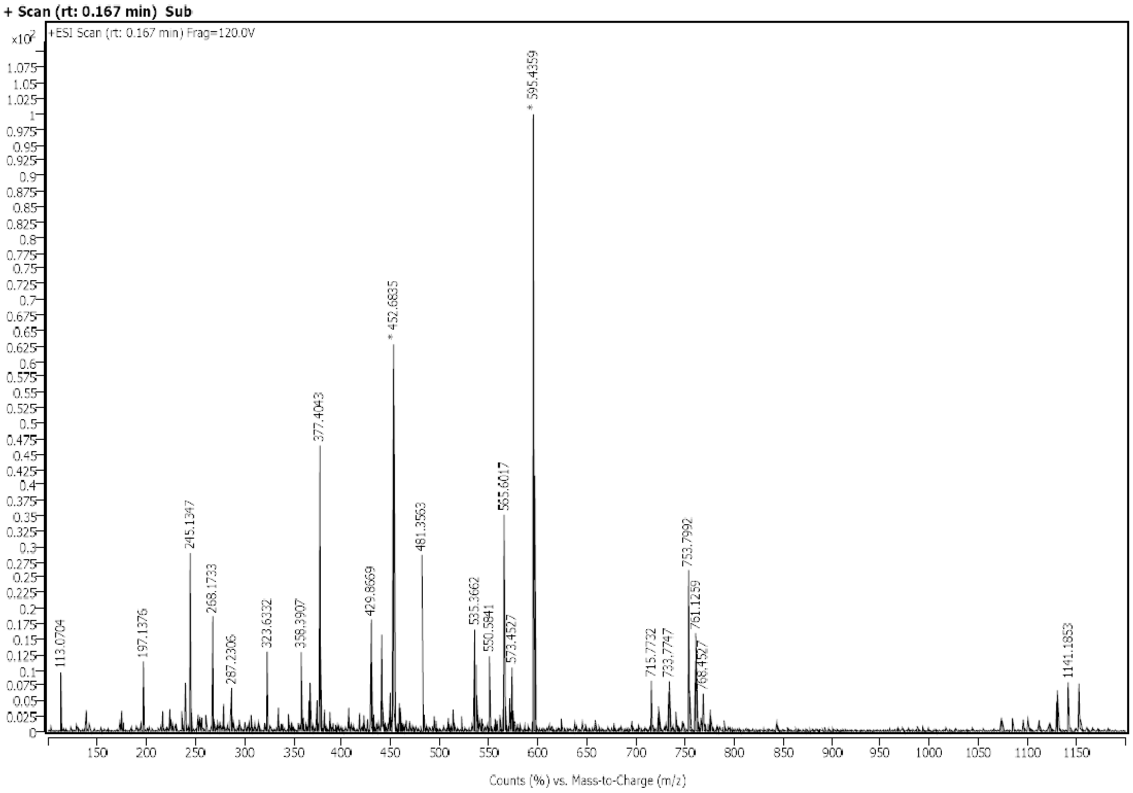

Fig. S23. Mass spectrum (HRESI) of **G1-1,3-alt**

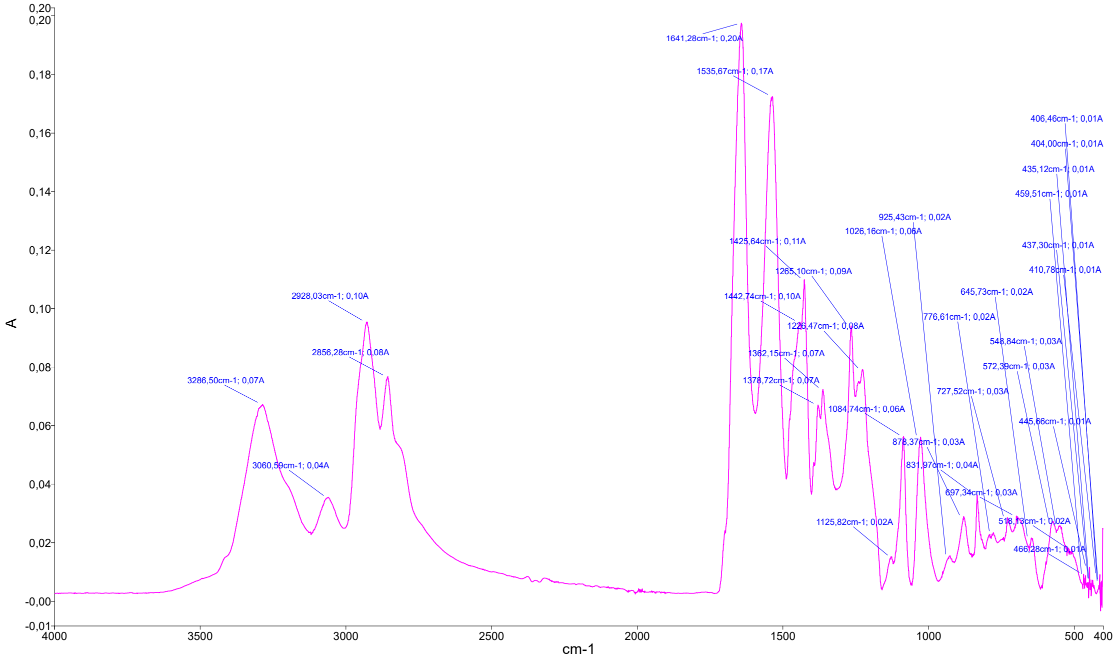

Fig. S24. FTIR-ATR spectrum of **G1-1,3-alt**

2. Complexation investigation

2.1. UV-Vis spectra

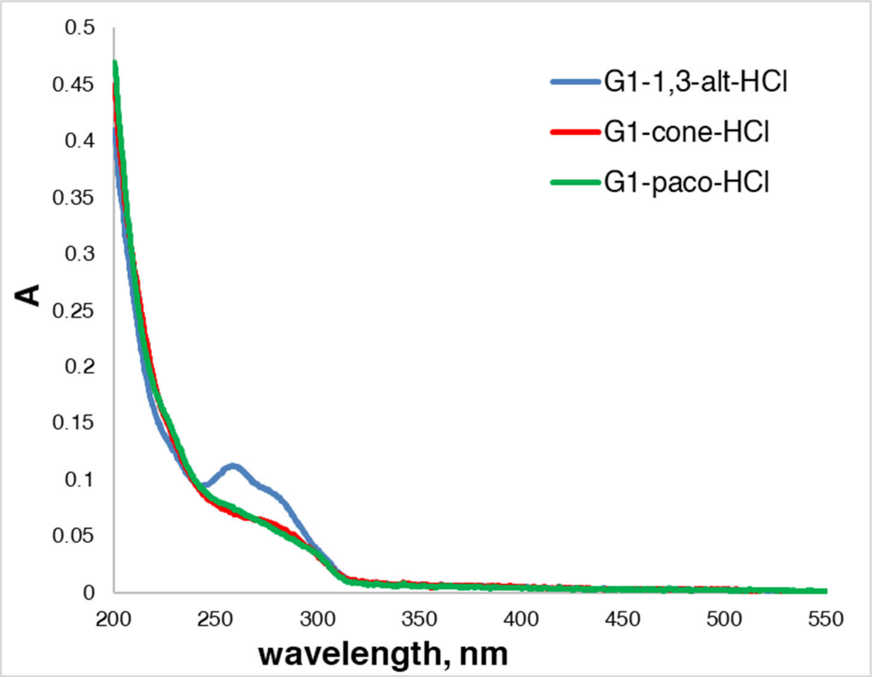

Fig. S25. UV-Vis absorption spectra of **G1-HCl** compounds (3.33 μM) in different conformations, in 10 mM Tris-HCl, pH 7.4.

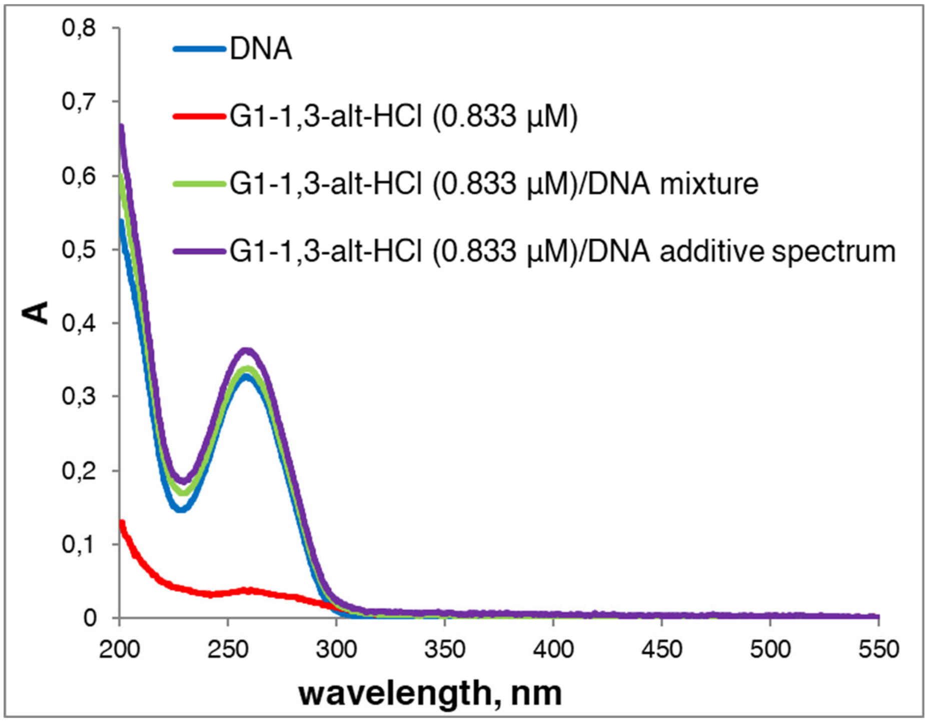

Fig. S26. UV-Vis absorption spectra of DNA (1.855×10^{-5} M base pairs), **G1-1,3-alt-HCl** (0.833 μM), and their mixture in 10 мM Tris-HCl, pH 7.4.

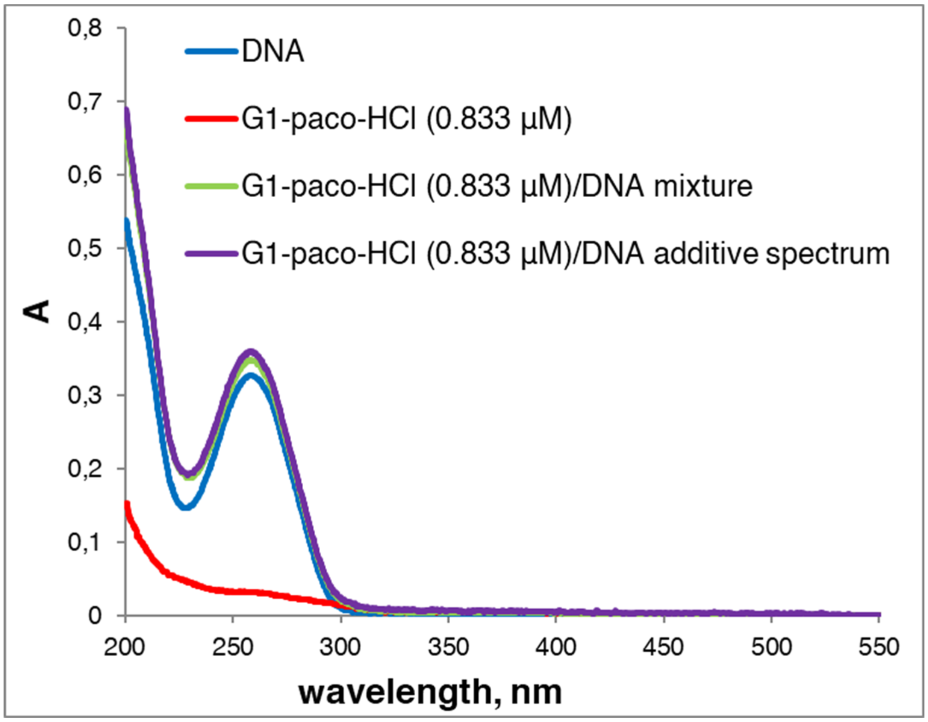

Fig. S27. UV-Vis absorption spectra of DNA (1.855×10^{-5} M base pairs), **G1-paco-HCl** (0.833 μM), and their mixture in 10 мM Tris-HCl, pH 7.4.

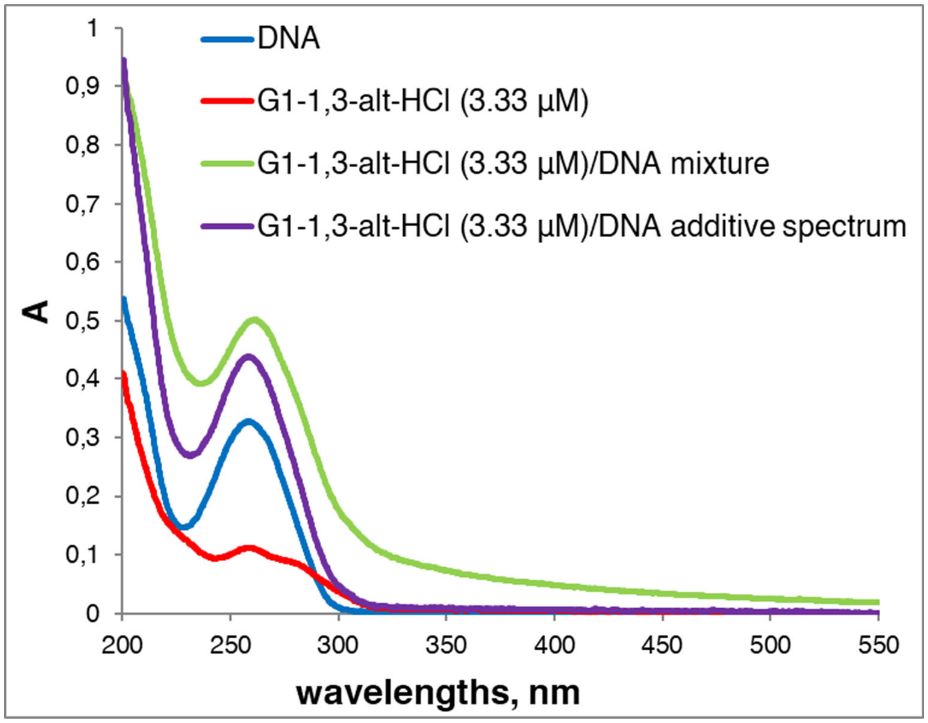

Fig. S28. UV-Vis absorption spectra of DNA (1.855×10^{-5} M base pairs), **G1-1,3-alt-HCl** (3.33 μM), and their mixture in 10 мM Tris-HCl, pH 7.4.

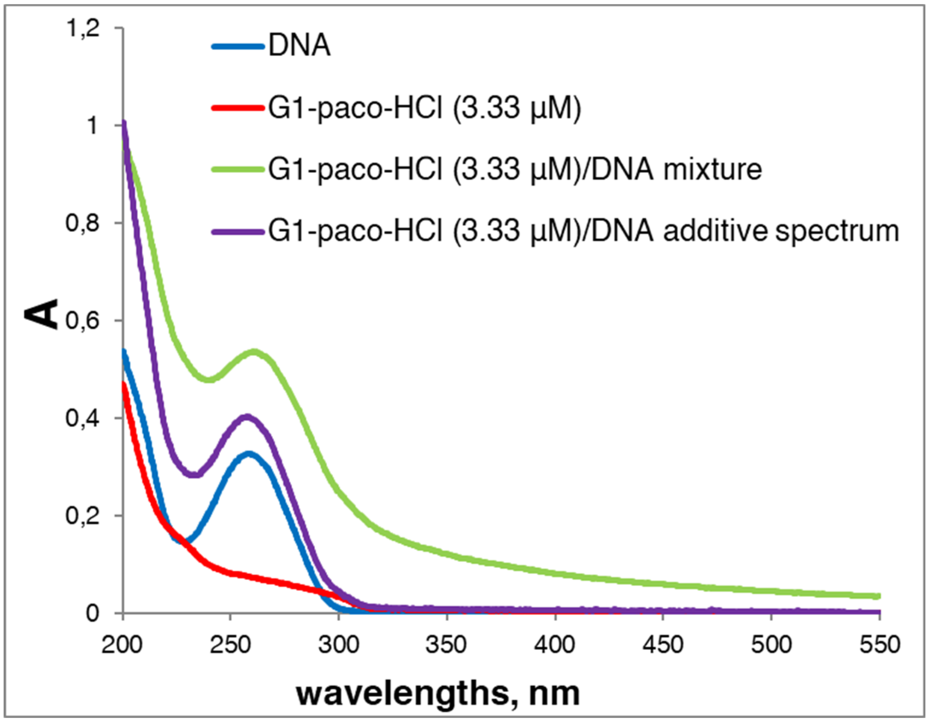

Fig. S29. UV-Vis absorption spectra of DNA (1.855×10^{-5} M base pairs), **G1-paco-HCl** (3.33 µM), and their mixture in 10 мM Tris-HCl, pH 7.4.

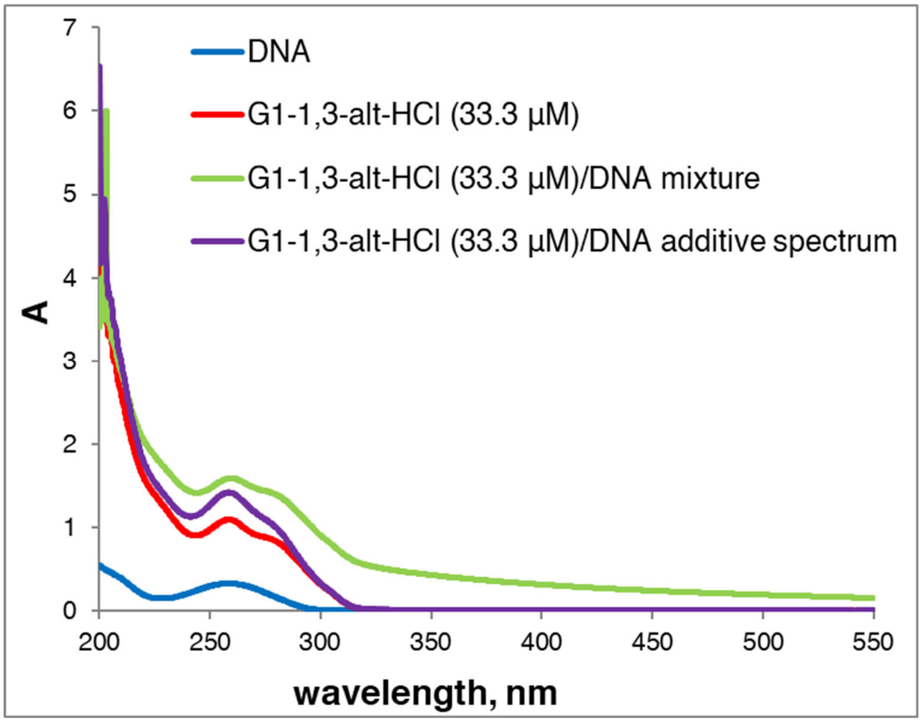

Fig. S30. UV-Vis absorption spectra of DNA (1.855×10^{-5} M base pairs), **G1-1,3-alt-HCl** (33.3 µM), and their mixture in 10 мM Tris-HCl, pH 7.4.

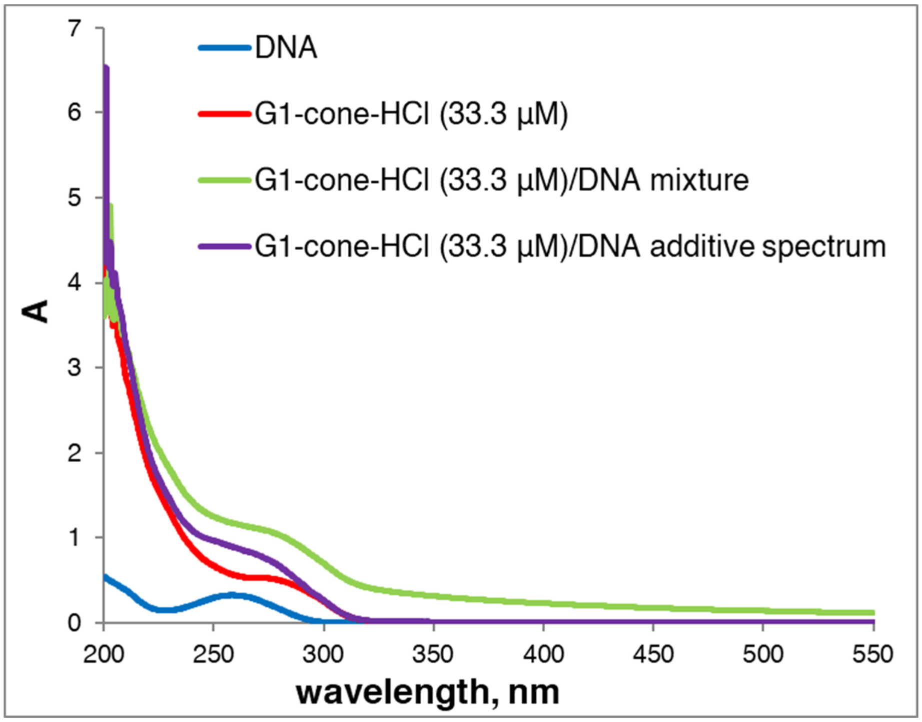

Fig. S31. UV-Vis absorption spectra of DNA (1.855×10^{-5} M base pairs), **G1-cone-HCl** (33.3 μM), and their mixture in 10 мM Tris-HCl, pH 7.4.

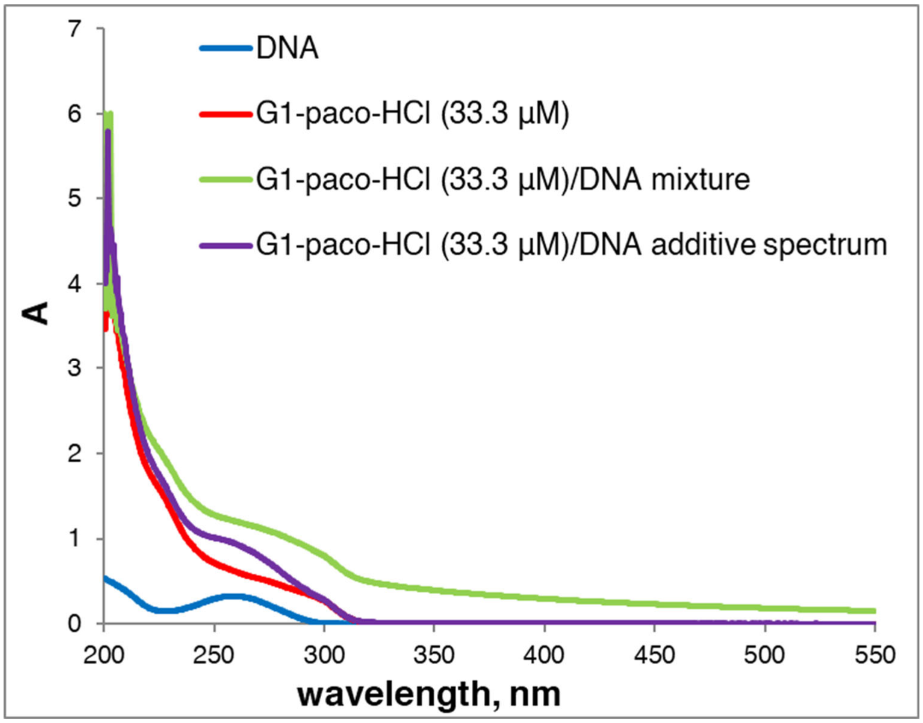

Fig. S32. UV-Vis absorption spectra of DNA (1.855×10^{-5} M base pairs), **G1-paco-HCl** (33.3 μM), and their mixture in 10 мM Tris-HCl, pH 7.4.

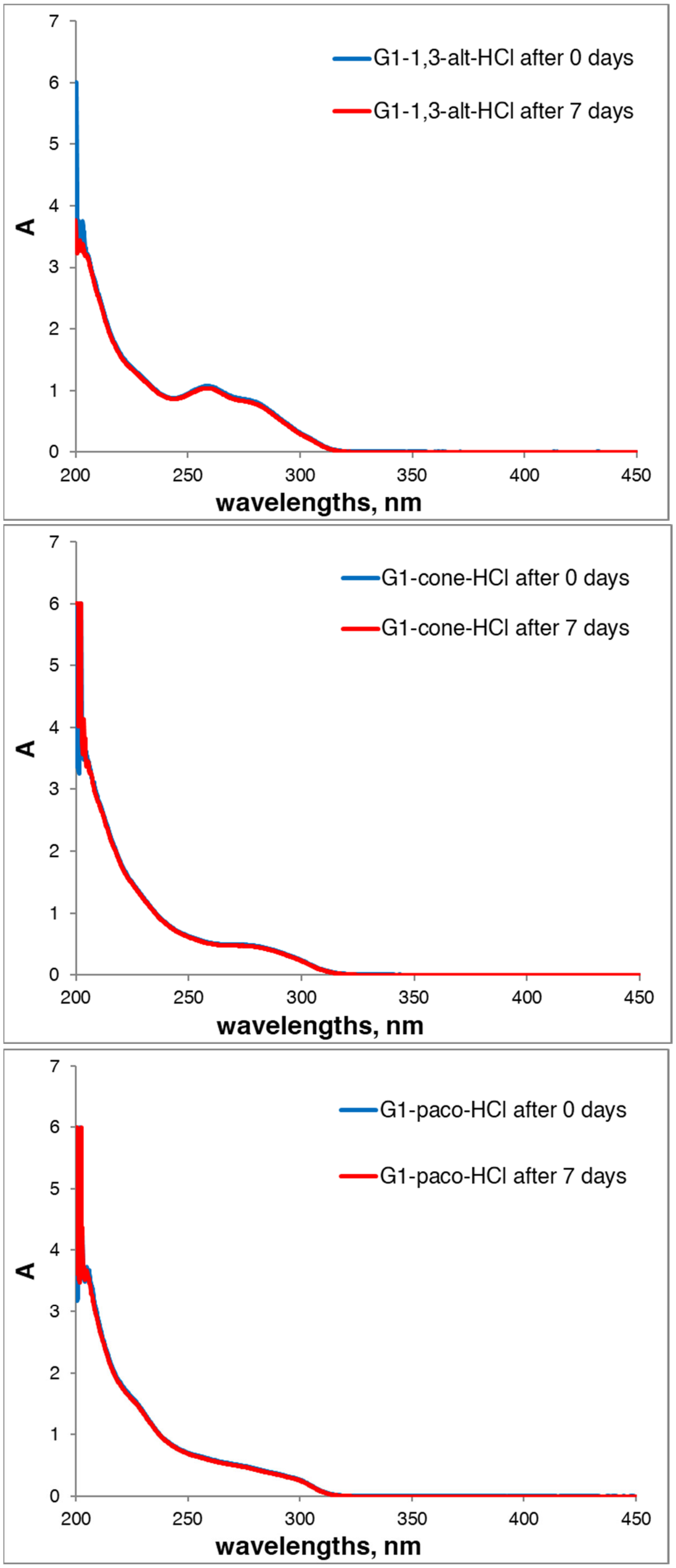

Fig. S33. UV-Vis spectra of compounds **G1-HCl** (33.3 μM) in different conformations in 10 мM Tris-HCl (pH 7.4) immediately and after 7 days' storage at room temperature.

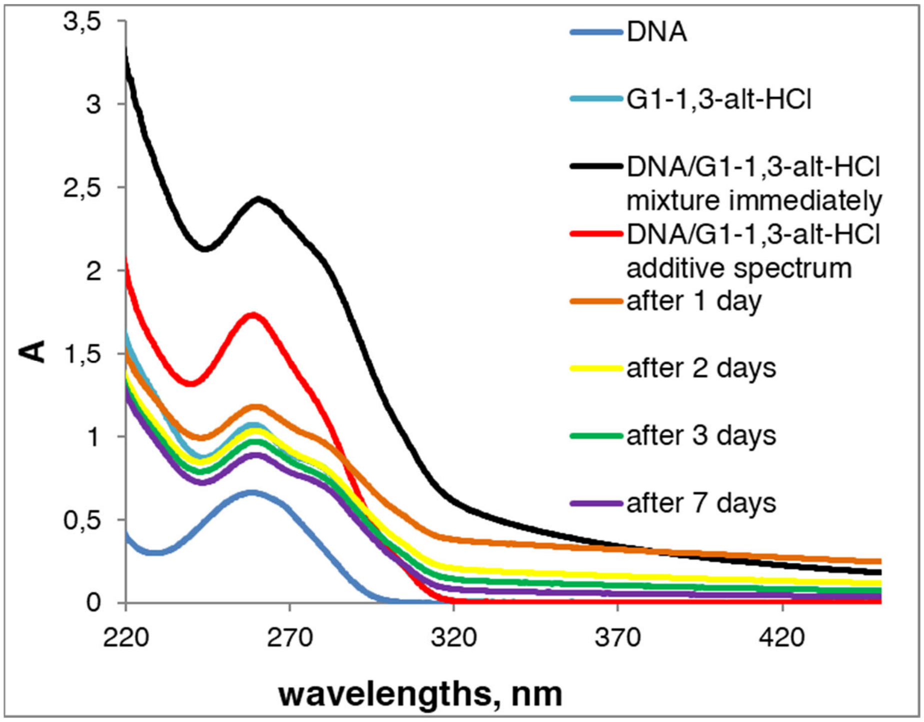

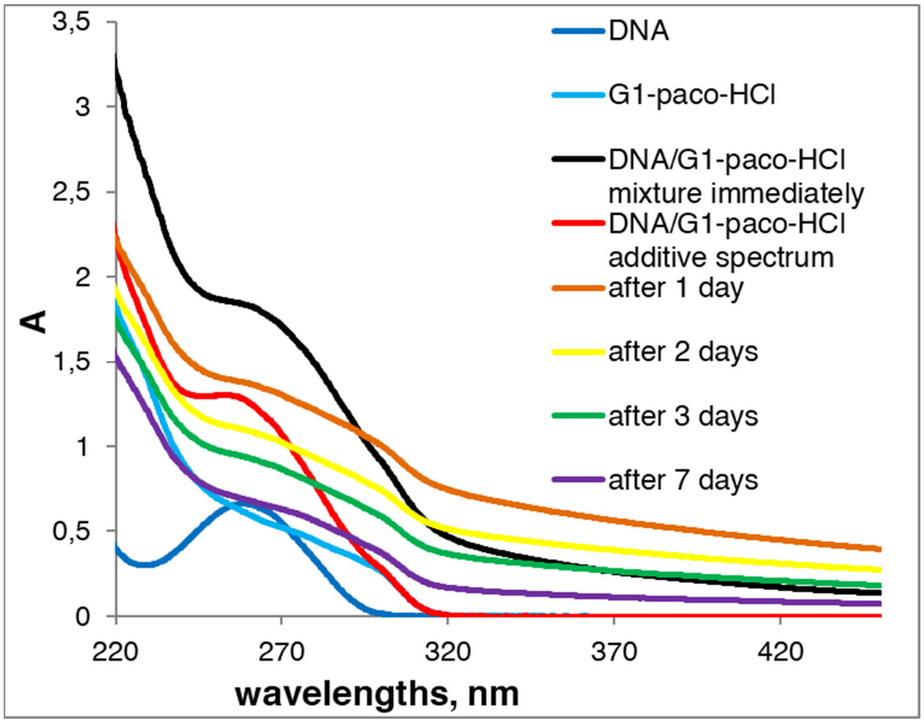

Fig. S34. UV-Vis spectra of compounds **G1-paco-HCl** and **G1-1,3-alt-HCl** (33.3 μM), DNA (3.710×10^{-5} M base pairs) and their mixture (10 мM Tris-HCl, pH 7.4) after storage at room temperature.

2.2 Fluorescence spectra

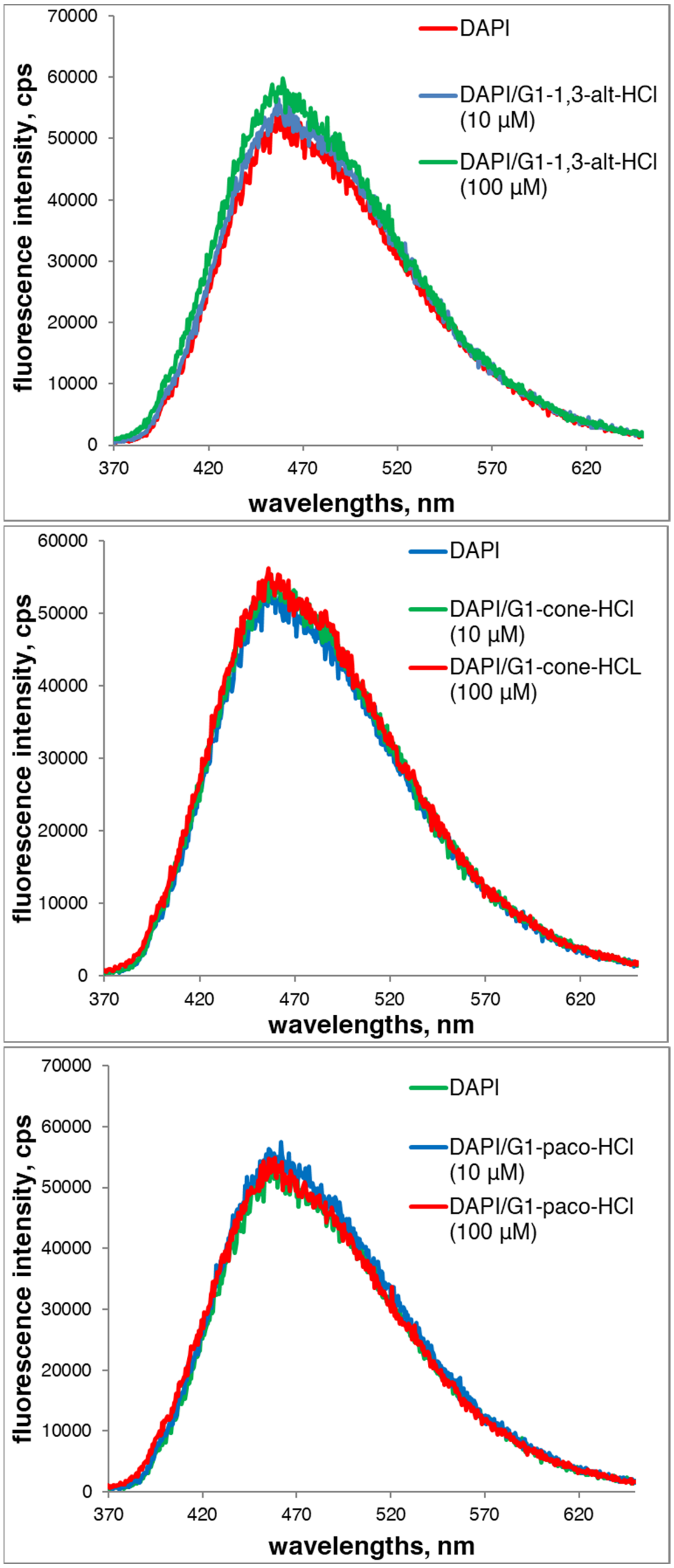

Fig. S35. Emission spectra of DAPI (10 μM) in presence of different conformations **G1-HCl** (10 and 100 μM) in 10 mM Tris-HCl, pH 7.4.

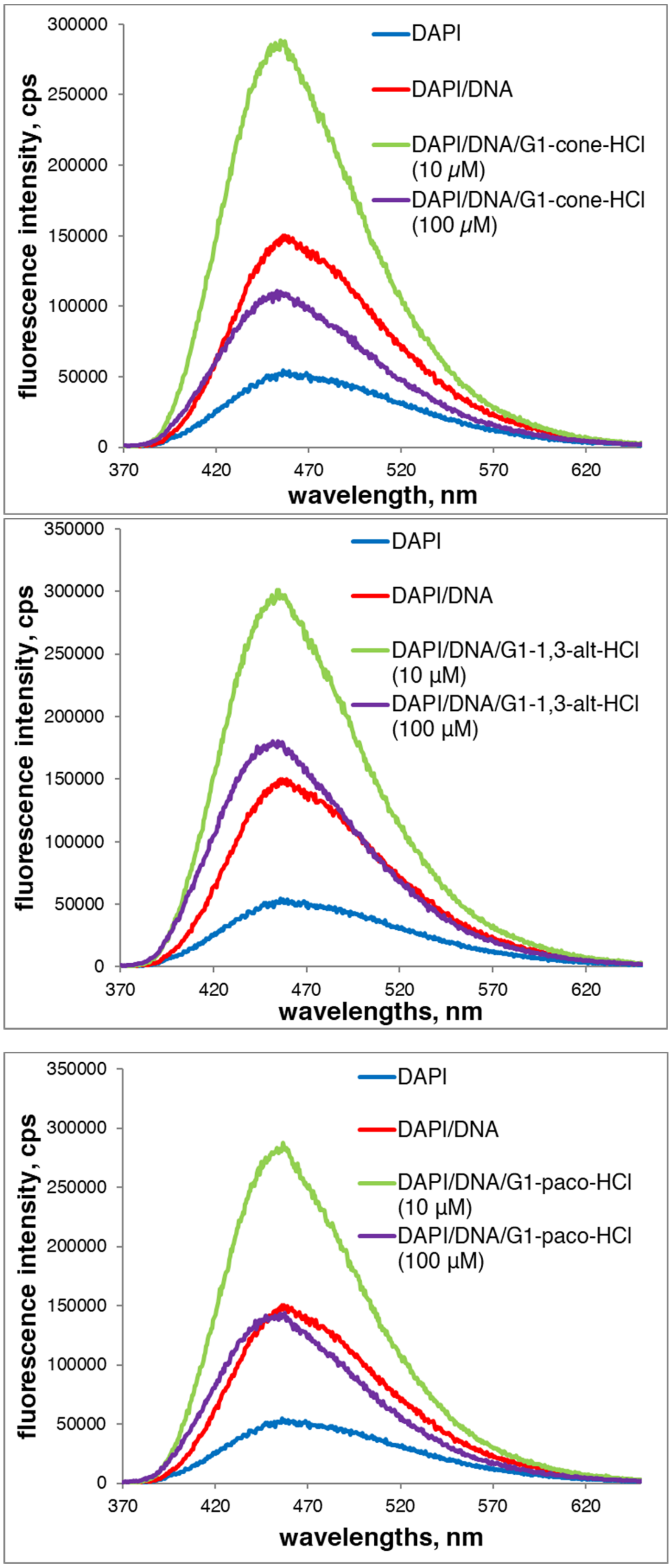

Fig. S36. Emission spectra of DAPI/DNA in presence of 10 and 100 μM **G1-HCl**: DAPI added to DNA/dendrimer mixture.

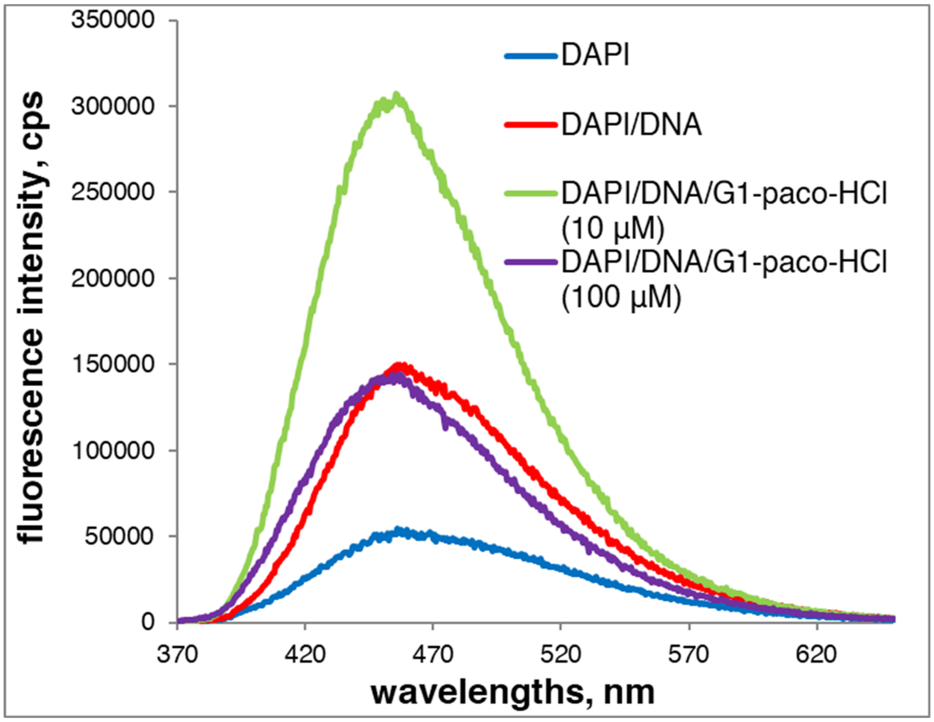

Fig. S37. Emission spectra of DAPI, DAPI/DNA and DAPI/DNA in the presence of 10 µM and 100 µM of **G1-paco-HCl**, in 10 mM Tris-HCl buffer, pH = 7.4: dendrimers added to DNA/DAPI mixture.

2.3. CD spectra

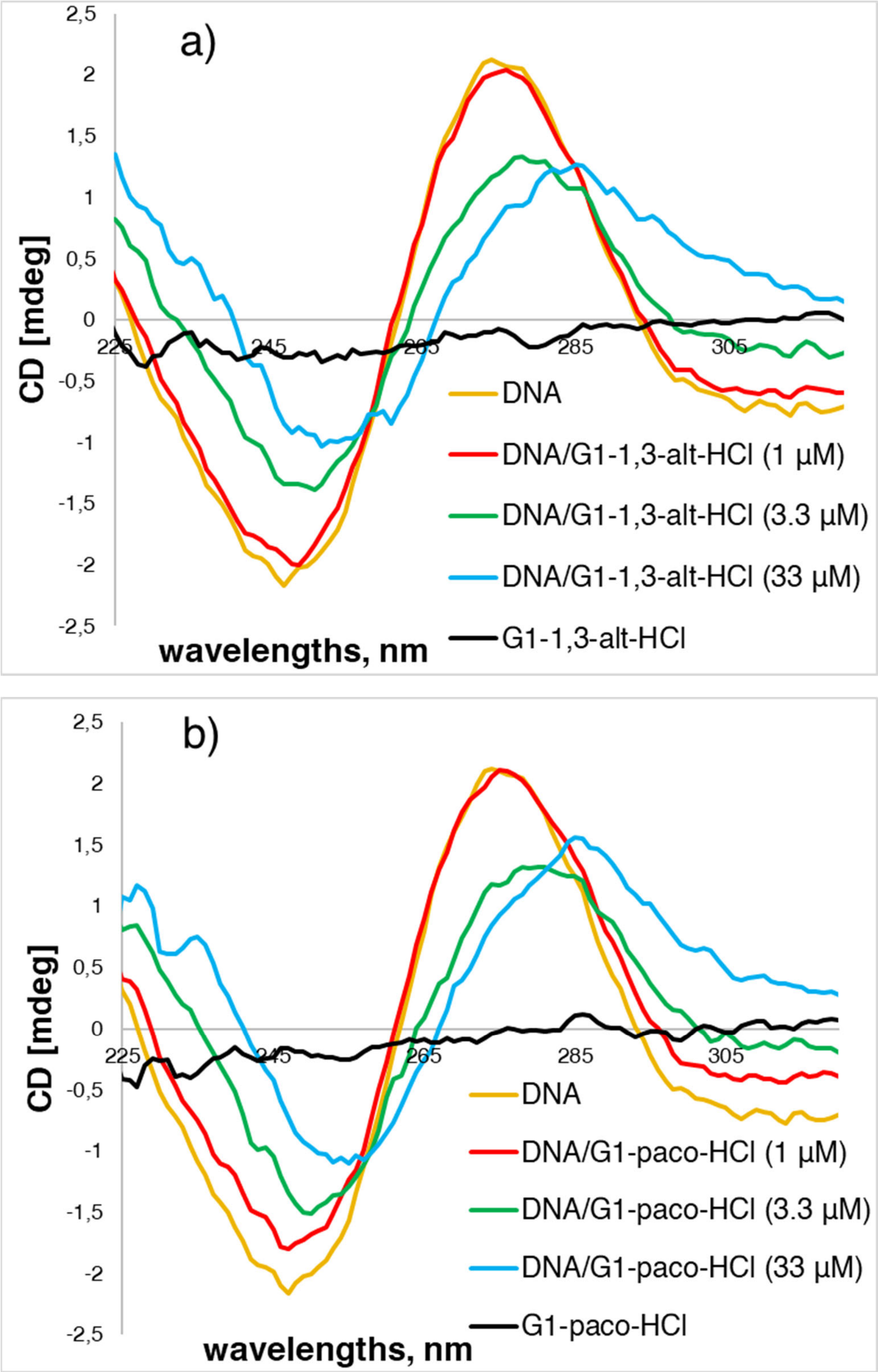

Fig. S38. CD spectra of salmon sperm DNA alone and in the presence of different concentrations of a) **G1-1,3-alt-HCl** and b) **G1-paco-HCl in** 10 mM Tris-HCl, pH 7.4.

2.4. DLS data

Table S1. Size distributions (by intensity) of G1-HCl aggregates (10 mM Tris-HCl, pH 7.4).

Compound	C (**G1-HCl**), μM	d_1, nm(%)	d_2, nm(%)	d_3, nm(%)	PDI
**G1-1,3-alt-HCl**	500	178.9±46.2 (71.3±16.7)	359.7±315.6 (22.5±17.9)	843.1±2056 (3.7±3.1)	0.532±0.087
	100	316.6±127.3 (70.2±19.3)	57.2±59.1 (22.5±17.6)	1823±2818 (5.1±2.0)	0.398±0.053
	50	269.0±72.8 (83.7±7.9)	34.0±35.0 (9.6±4.9)	1799±2765 (3.9±2.2)	0.386±0.096
	10	243.5±52.5 (84.8±9.7)	896.6±2105.0 (10.8±8.5)	3519±2723 (4.5±2.4)	0.362±0.048
	5	260.8±40.0 (71.3±16.7)	4935±82.6 (8.6±1.1)	14.2±21.9 (1.9±3.0)	0.312±0.025
**G1-cone-HCl**	500	6.9±1.9 (45.8±9.4)	119.1±125.1 (26.7±4.2)	149.3±189.2 (18.1±7.2)	0.345±0.104
	100	78.7±176.2 (47.5±6.0)	288.7±159.0 (39.4±5.1)	1802±2708 (9.7±4.1)	0.412±0.199
	50	406.2±136.4 (56.8±7.4)	7.6±2.0 (31.1±9.6)	3376±2612 (10.3±2.6)	0.466±0.188
	10	226.7±23.0 (65.7±9.0)	825.0±1988 (18.9±5.2)	3325±2570 (12.8±3.7)	0.352±0.063
	5	206.3±41.8 (65.5±8.1)	849.0±2035 (20.0±4.6)	2502±2728 (12.4±3.4)	0.347±0.141
**G1-paco-HCl**	500	378.1±36.6 (63.4±6.2)	2.2±0.6 (26.7±7.8)	931.2±2268 (9.0±4.0)	0.554±0.147
	100	439.1±33.7 (64.2±5.7)	3.3±1.0 (19.1±5.2)	27.1±23.3 (10.8±2.8)	0.626±0.120
	50	337.6±53.4(87.8±3.7)	2.9±0.6 (11.9±3.2)	926.7±2270 (0.3±0.7)	0.539±0.064
	10	337.8±61.0 (82.8±6.9)	30.4±4.5 (14.7±4.3)	2.6±5.6 (2.5±5.2)	0.699±0.113
	5	356.3±101.6 (86.8±1.9)	34.1±2.9 (12.7±2.3)	928.9±2269 (0.6±0.9)	0.550±0.074

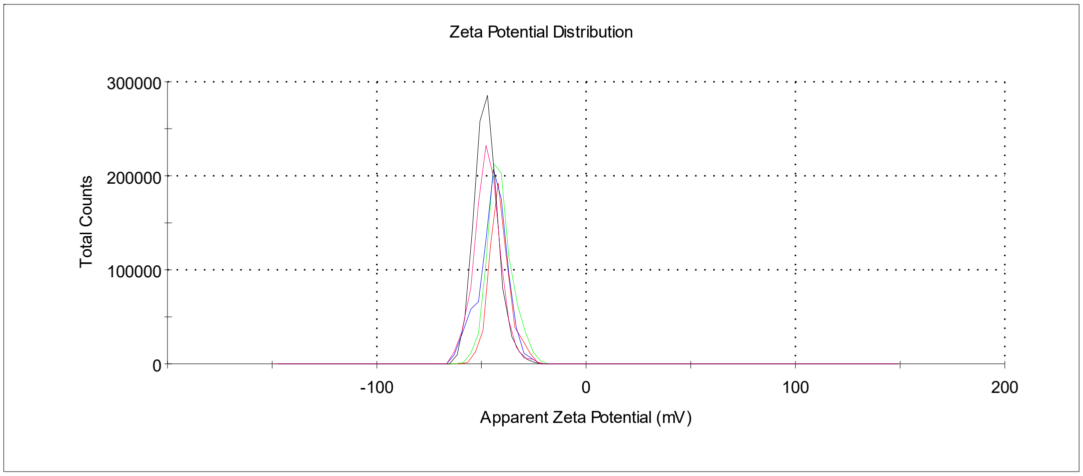

Fig. S39. Zeta-potential distributions of **G1-1,3-alt-HCl** (5 μM) + DNA (5.565×10^{-5} M base pairs) aggregates.

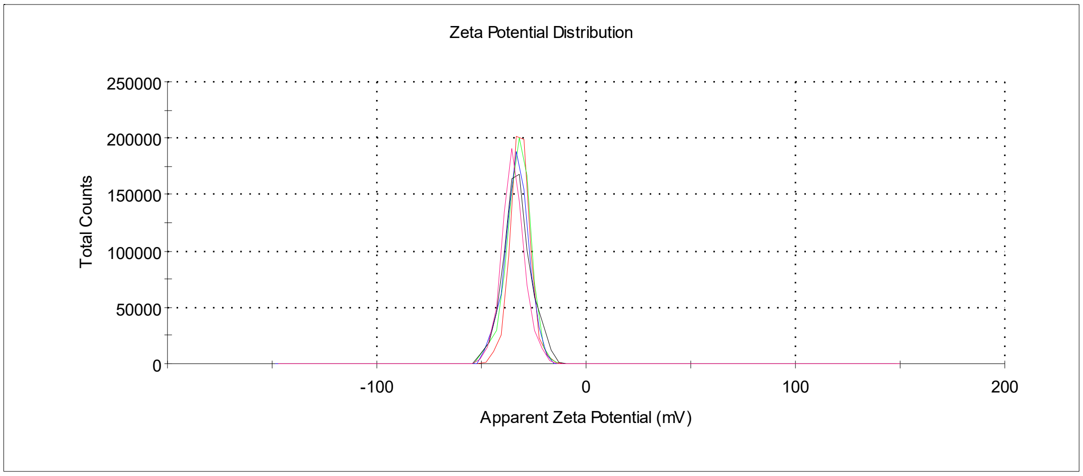

Fig. S40. Zeta-potential distributions of **G1-1,3-alt-HCl** (10 μM) + DNA (5.565×10^{-5} M base pairs) aggregates.

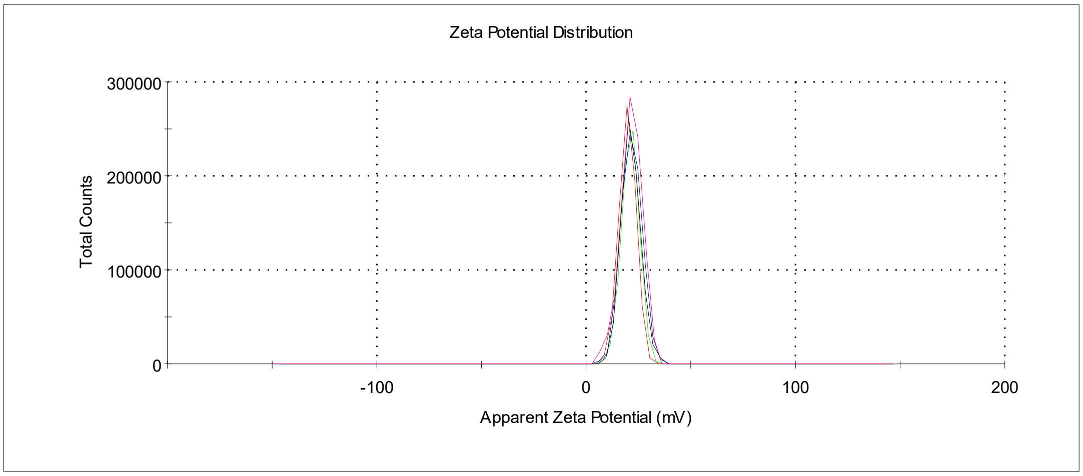

Fig. S41. Zeta-potential distributions of **G1-1,3-alt-HCl** (50 μM) + DNA (5.565×10^{-5} M base pairs) aggregates.

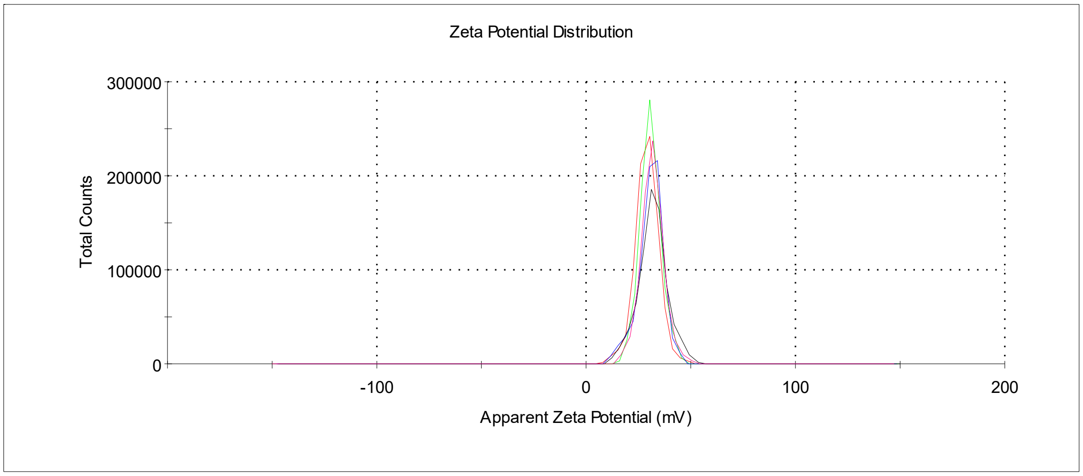

Fig. S42. Zeta-potential distributions of **G1-1,3-alt-HCl** (100 μM) + DNA (5.565×10^{-5} M base pairs) aggregates.

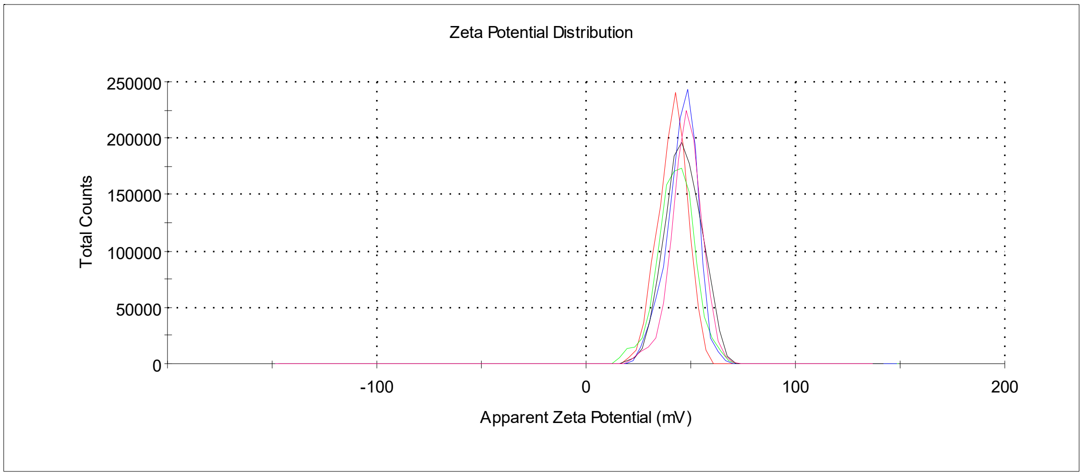

Fig. S43. Zeta-potential distributions of **G1-1,3-alt-HCl** (500 μM) + DNA (5.565×10^{-5} M base pairs) aggregates.

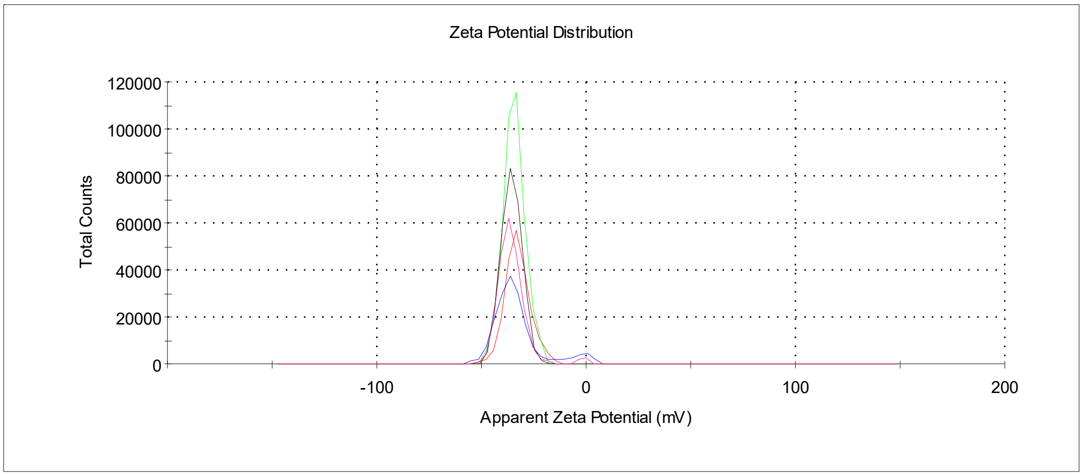

Fig. S44. Zeta-potential distributions of **G1-paco-HCl** (5 μM) + DNA (5.565×10^{-5} M base pairs) aggregates.

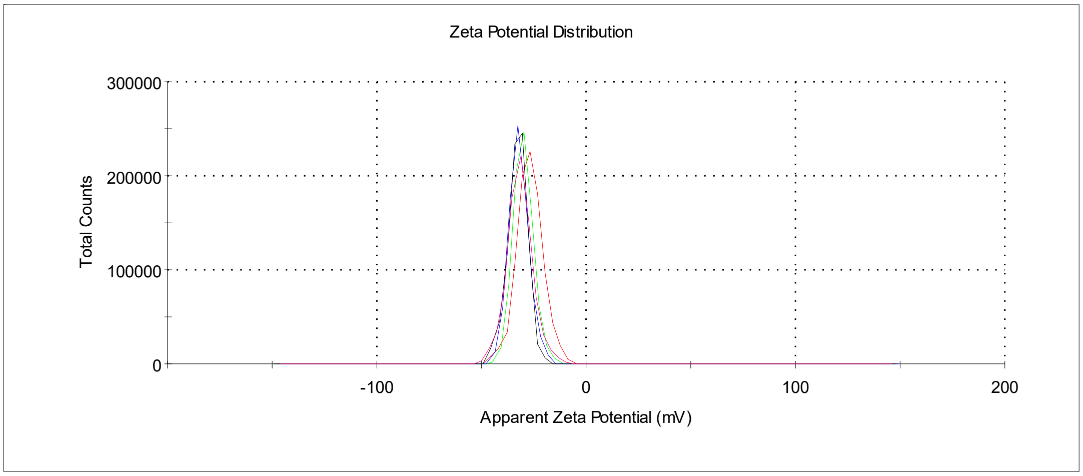

Fig. S45. Zeta-potential distributions of **G1-paco-HCl** (10 μM) + DNA (5.565×10^{-5} M base pairs) aggregates.

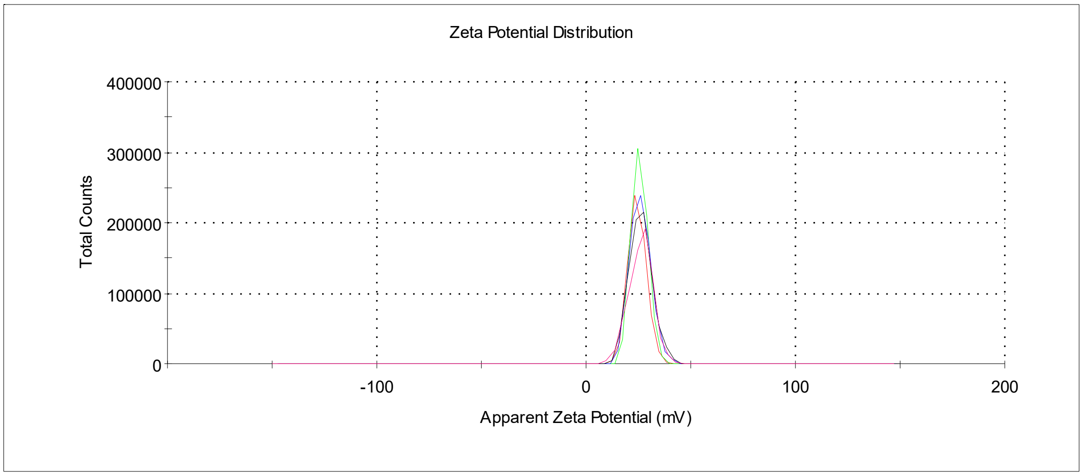

Fig. S46. Zeta-potential distributions of **G1-paco-HCl** (50 μM) + DNA (5.565×10^{-5} M base pairs) aggregates.

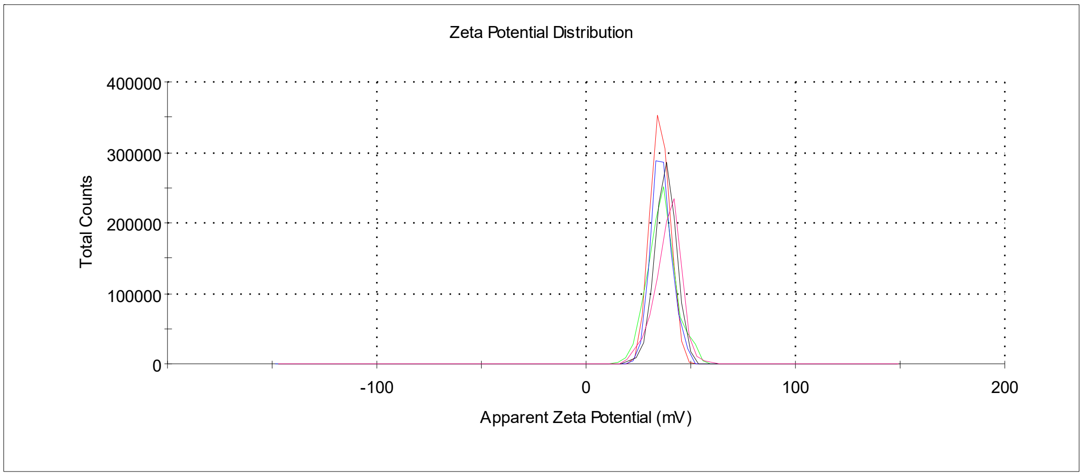

Fig. S47. Zeta-potential distributions of **G1-paco-HCl** (100 μM) + DNA (5.565×10^{-5} M base pairs) aggregates.

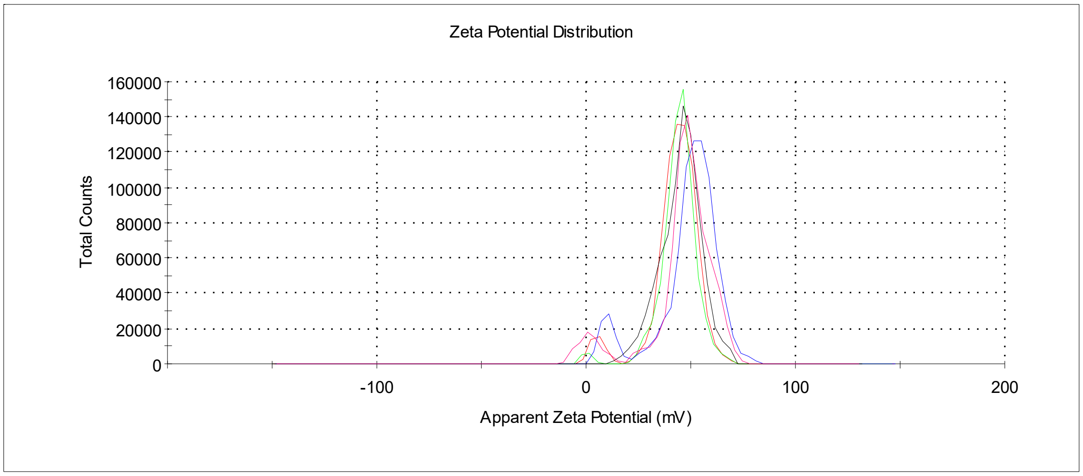

Fig. S48. Zeta-potential distributions of **G1-paco-HCl** (500 μM) + DNA (5.565×10^{-5} M base pairs) aggregates.

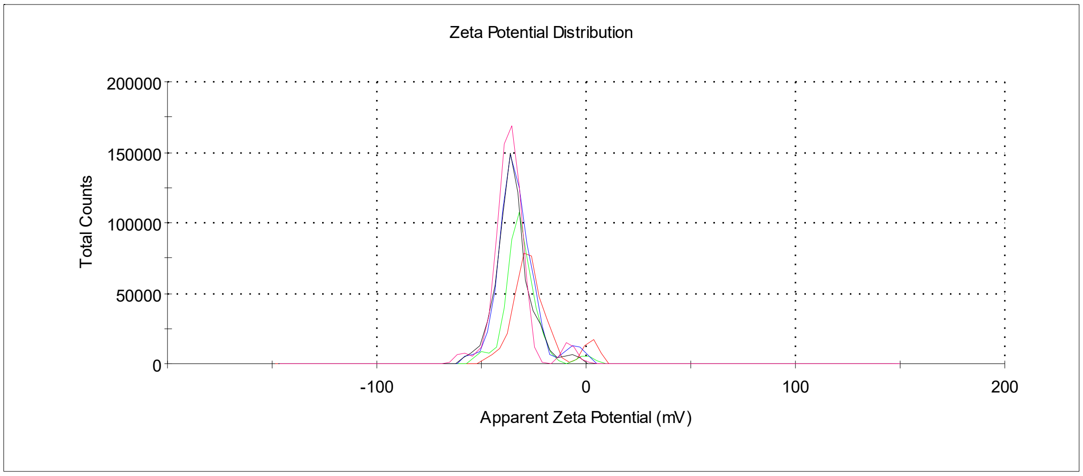

Fig. S49. Zeta-potential distributions of **G1-cone-HCl** (5 μM) + DNA (5.565×10^{-5} M base pairs) aggregates.

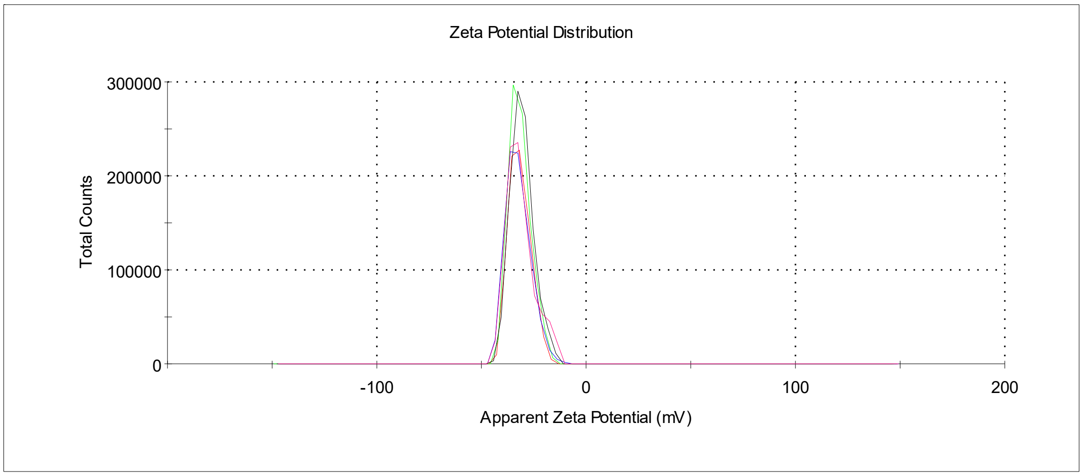

Fig. S50. Zeta-potential distributions of **G1-cone-HCl** (10 μM) + DNA (5.565×10^{-5} M base pairs) aggregates.

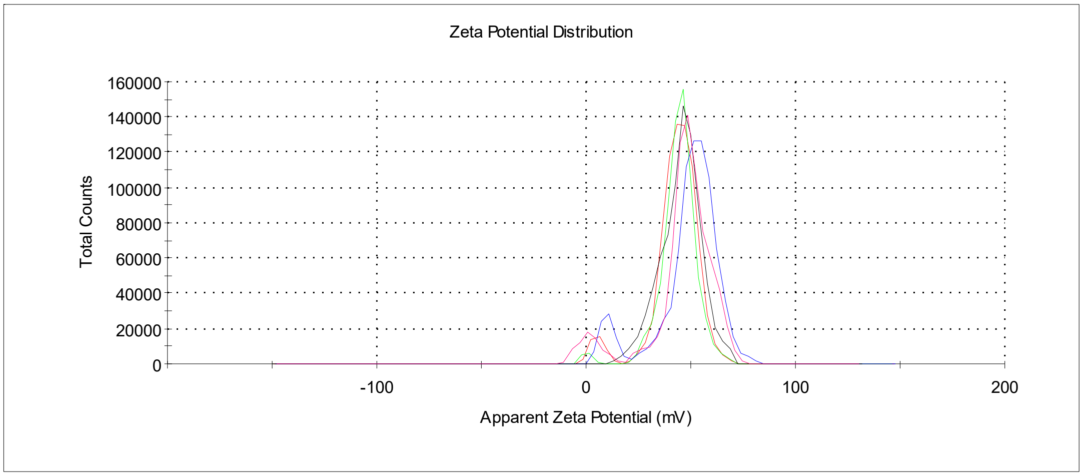

Fig. S51. Zeta-potential distributions of **G1-cone-HCl** (50 μM) + DNA (5.565×10^{-5} M base pairs) aggregates.

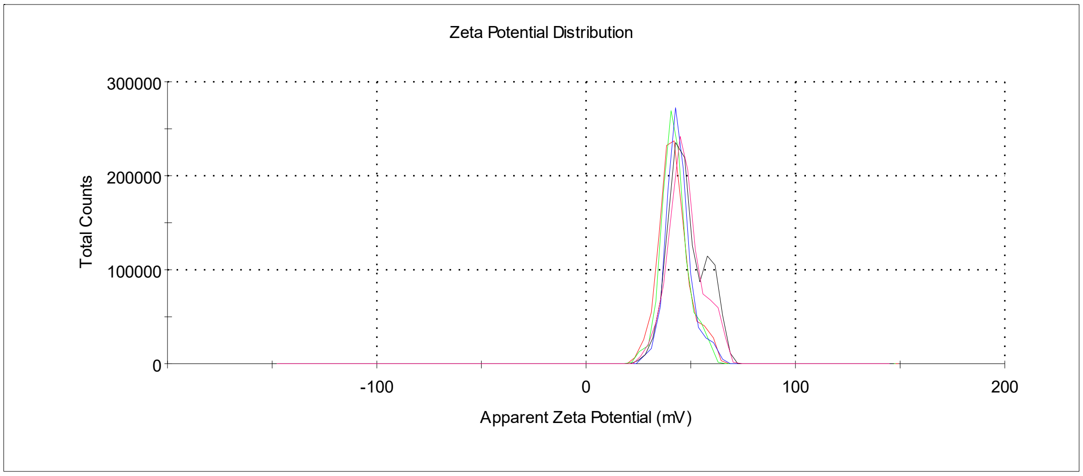

Fig. S52. Zeta-potential distributions of **G1-cone-HCl** (100 μM) + DNA (5.565×10^{-5} M base pairs) aggregates.

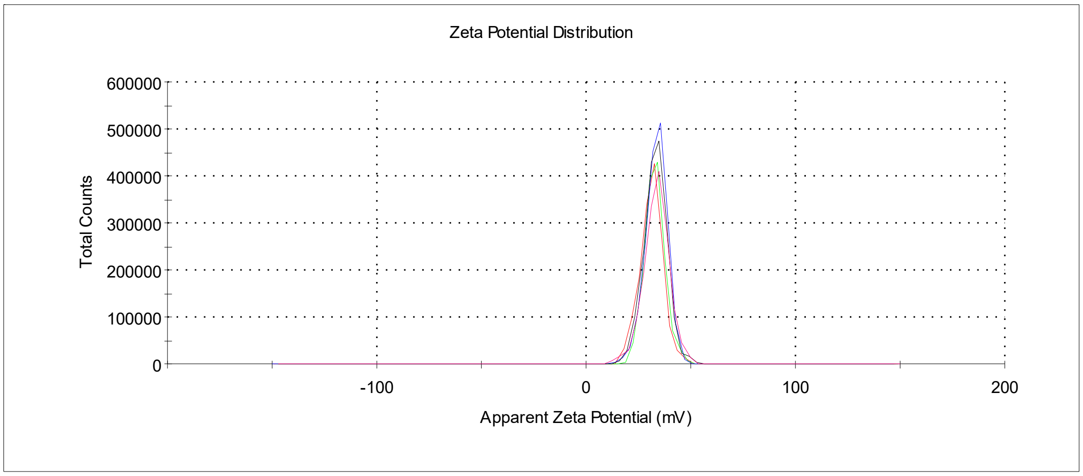

Fig. S53. Zeta-potential distributions of **G1-cone-HCl** (500 μM) + DNA (5.565×10^{-5} M base pairs) aggregates.

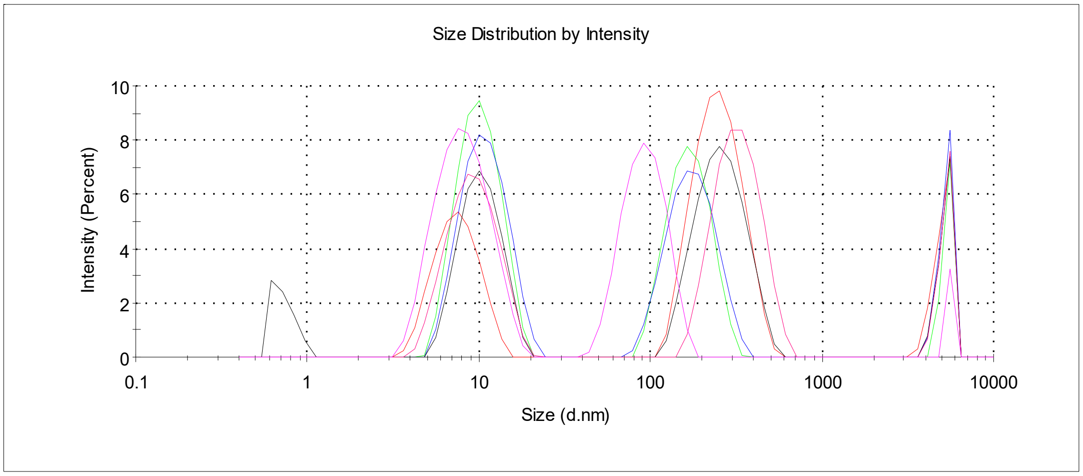

Fig. S54. Size distributions of DNA (5.565×10^{-5} M base pairs).

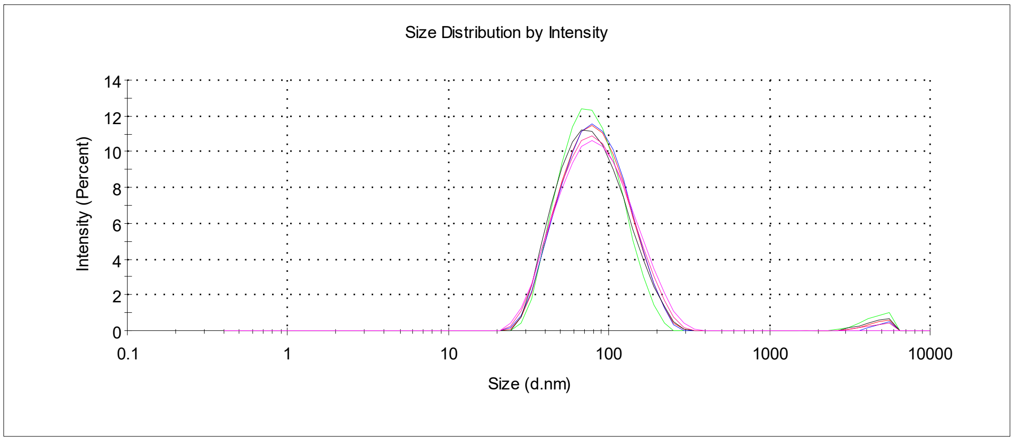

Fig. S55. Size distributions of **G1-1,3-alt-HCl** (500 μM) + DNA (5.565×10^{-5} M base pairs) aggregates.

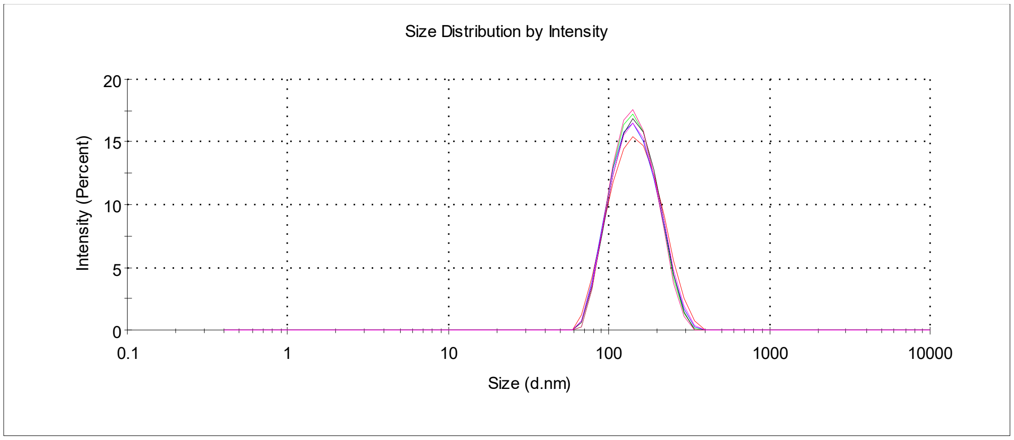

Fig. S56. Size distributions of **G1-1,3-alt-HCl** (100 μM) + DNA (5.565×10^{-5} M base pairs) aggregates.

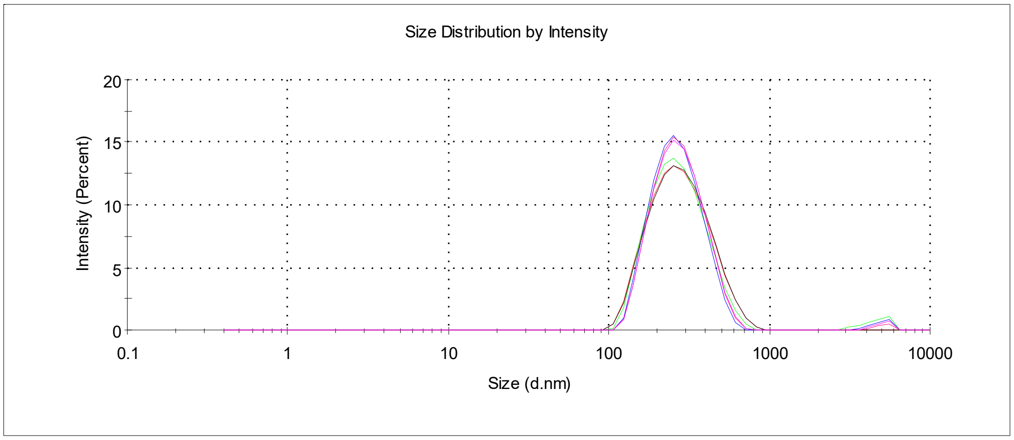

Fig. S57. Size distributions of **G1-1,3-alt-HCl** (50 μM) + DNA (5.565×10^{-5} M base pairs) aggregates.

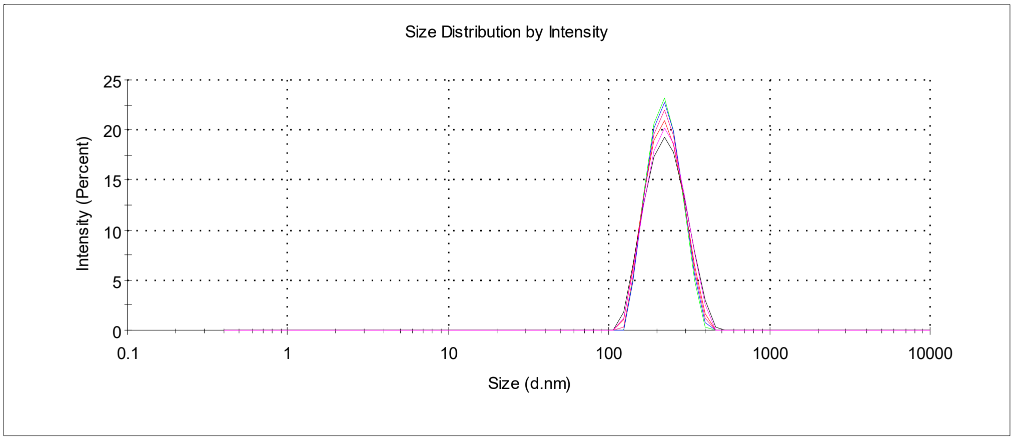

Fig. S58. Size distributions of **G1-1,3-alt-HCl** (10 μM) + DNA (5.565×10^{-5} M base pairs) aggregates.

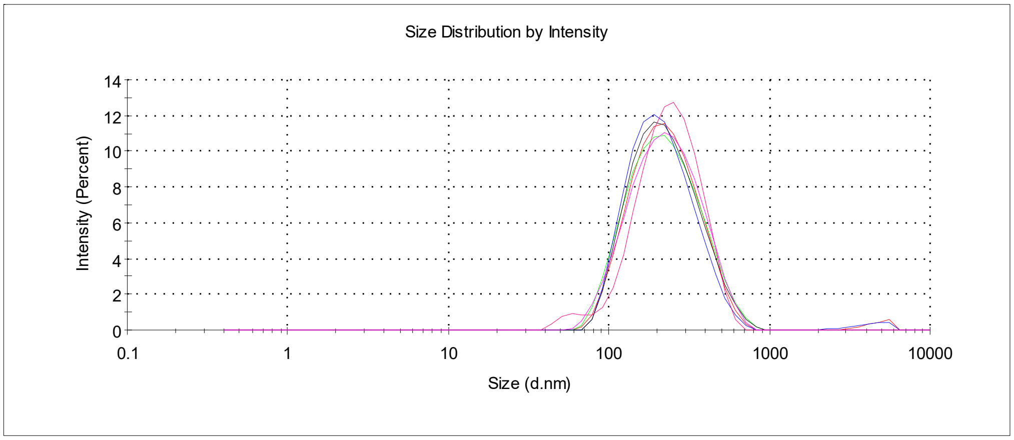

Fig. S59. Size distributions of **G1-1,3-alt-HCl** (5 μM) + DNA (5.565×10^{-5} M base pairs) aggregates.

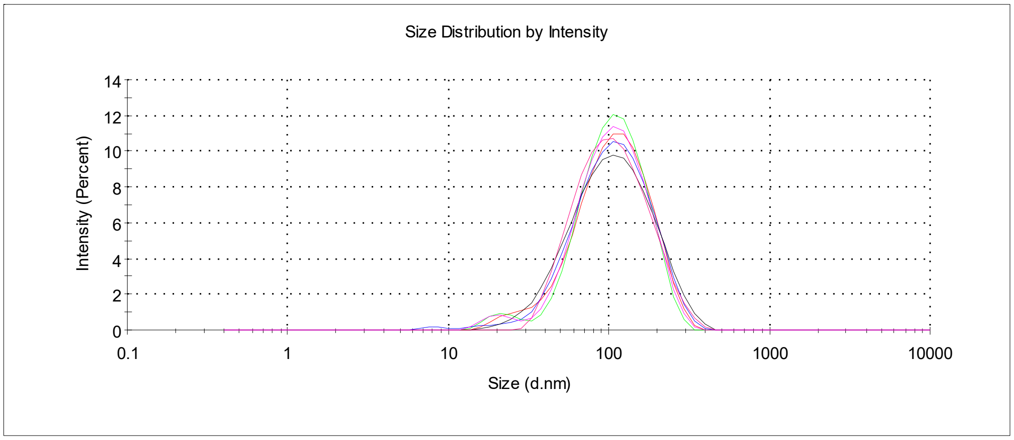

Fig. S60. Size distributions of **G1-cone-HCl** (500 μM) + DNA (5.565×10^{-5} M base pairs) aggregates.

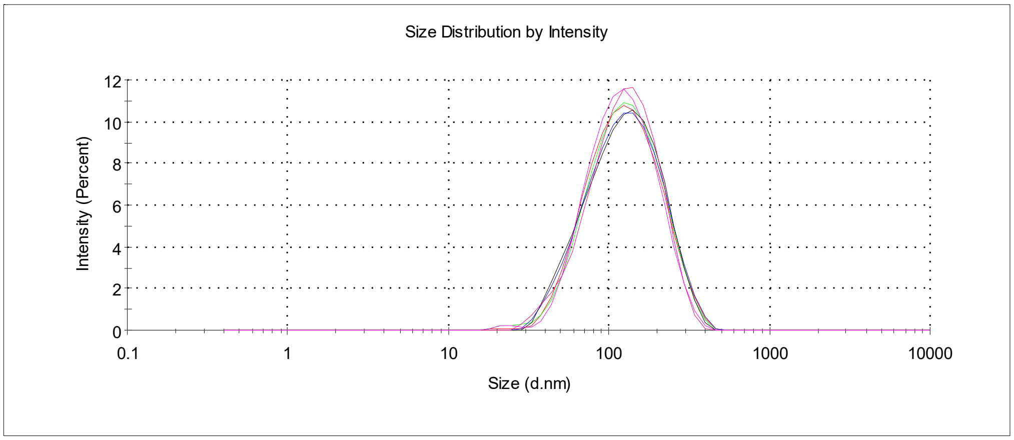

Fig. S61. Size distributions of **G1-cone-HCl** (100 μM) + DNA (5.565×10^{-5} M base pairs) aggregates.

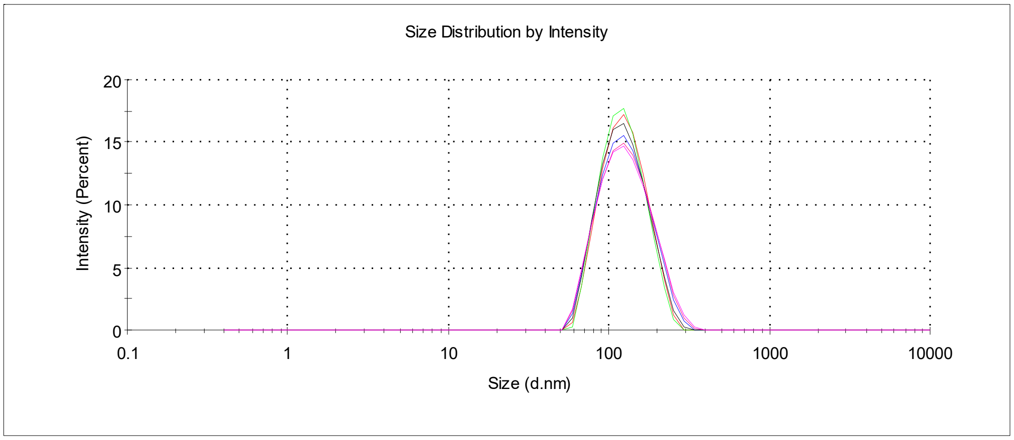

Fig. S62. Size distributions of **G1-cone-HCl** (50 μM) + DNA (5.565×10^{-5} M base pairs) aggregates.

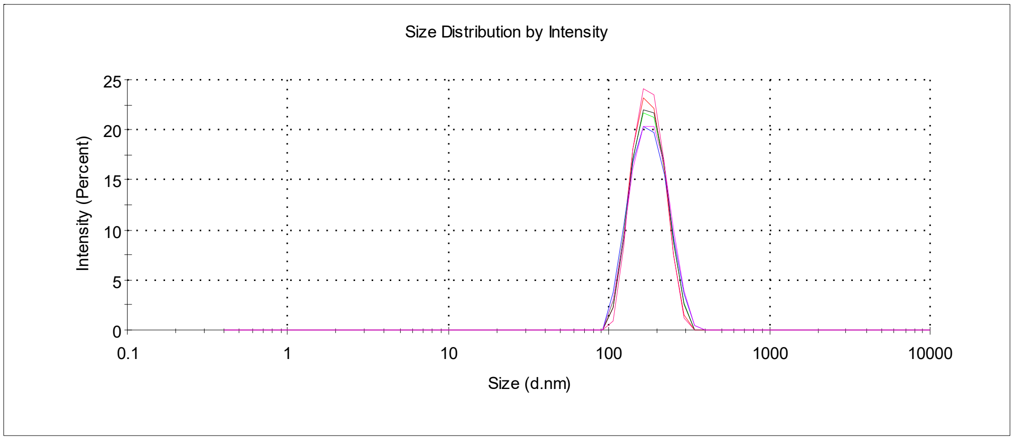

Fig. S63. Size distributions of **G1-cone-HCl** (10 μM) + DNA (5.565×10^{-5} M base pairs) aggregates.

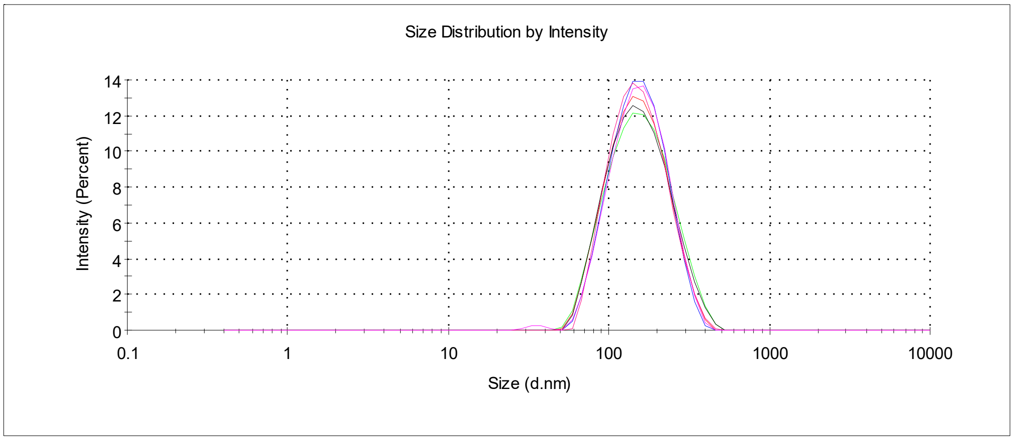

Fig. S64. Size distributions of **G1-cone-HCl** (5 μM) + DNA (5.565×10^{-5} M base pairs) aggregates.

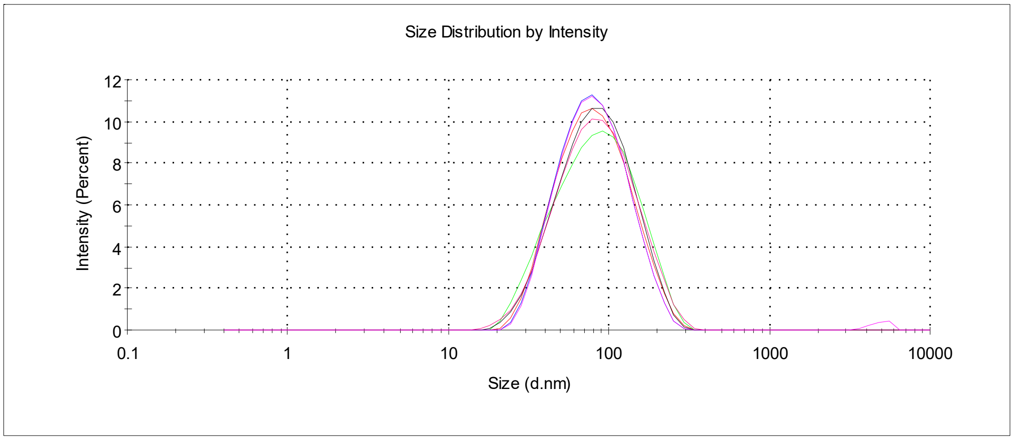

Fig. S65. Size distributions of **G1-paco-HCl** (500 μM) + DNA (5.565×10^{-5} M base pairs) aggregates.

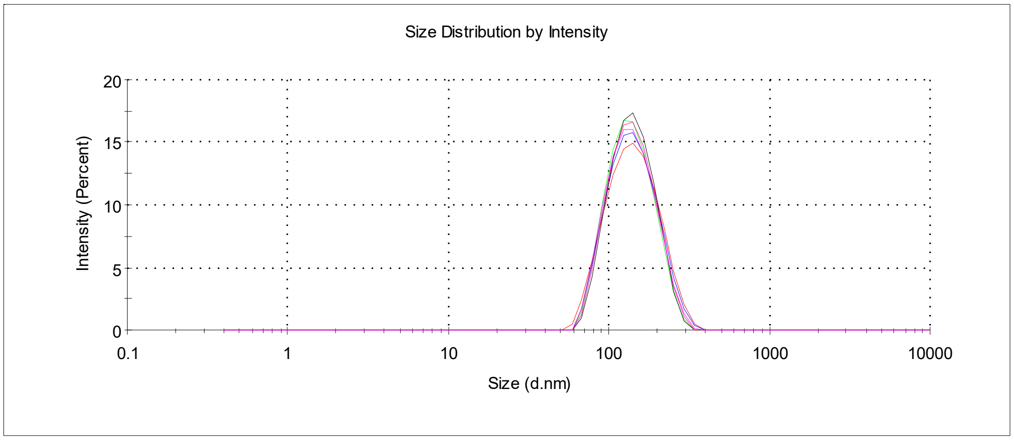

Fig. S66. Size distributions of **G1-paco-HCl** (100 μM) + DNA (5.565×10^{-5} M base pairs) aggregates.

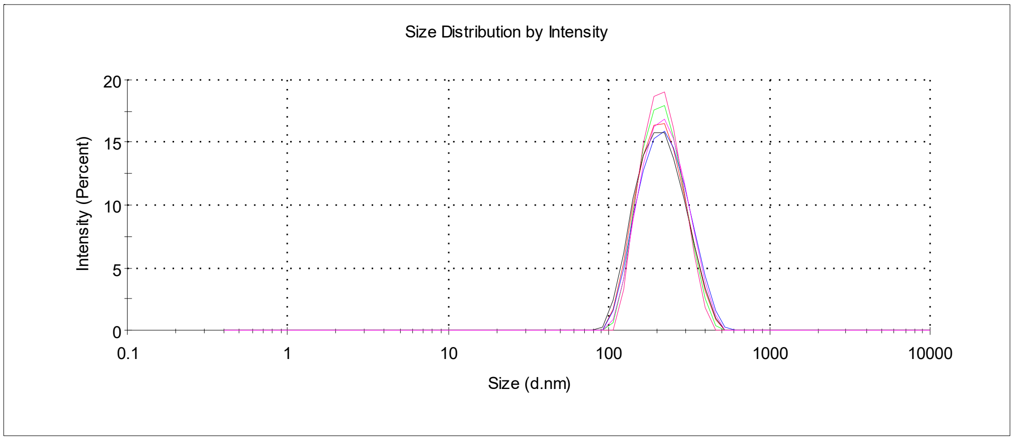

Fig. S67. Size distributions of **G1-paco-HCl** (50 μM) + DNA (5.565×10^{-5} M base pairs) aggregates.

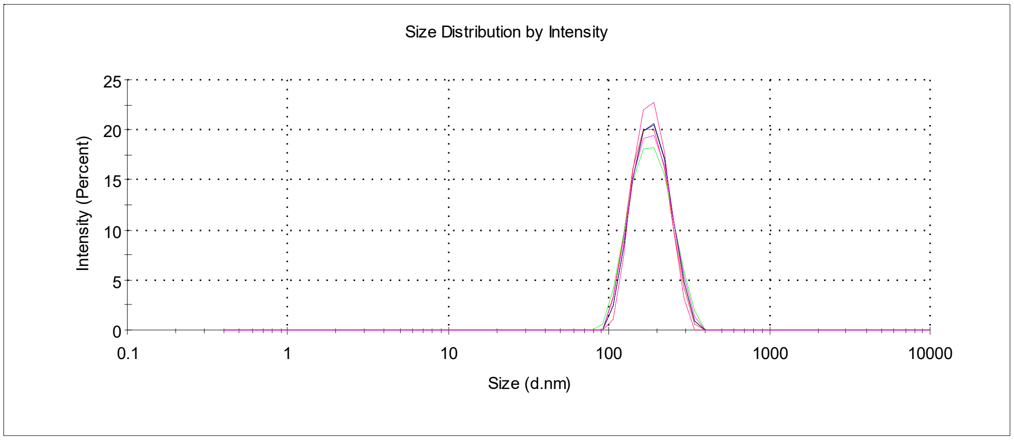

Fig. S68. Size distributions of **G1-paco-HCl** (10 μM) + DNA (5.565×10^{-5} M base pairs) aggregates.

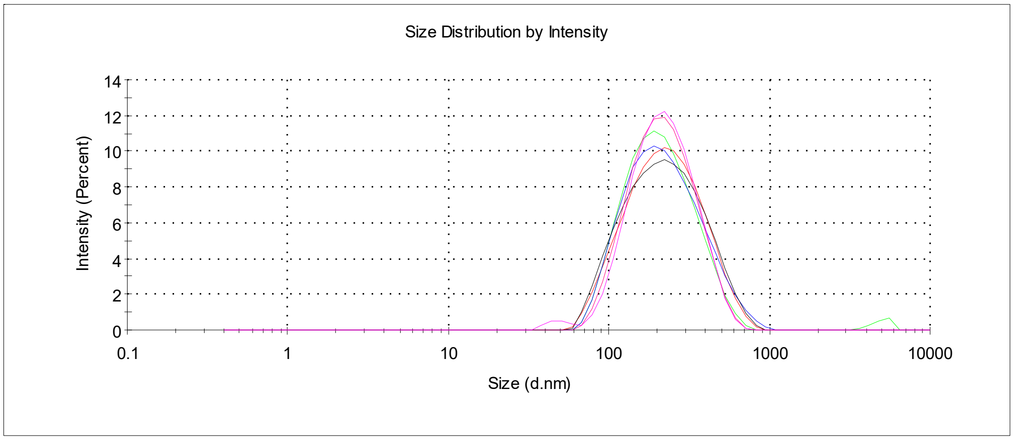

Fig. S69. Size distributions of **G1-paco-HCl** (5 μM) + DNA (5.565×10^{-5} M base pairs) aggregates.

2.5. TEM images

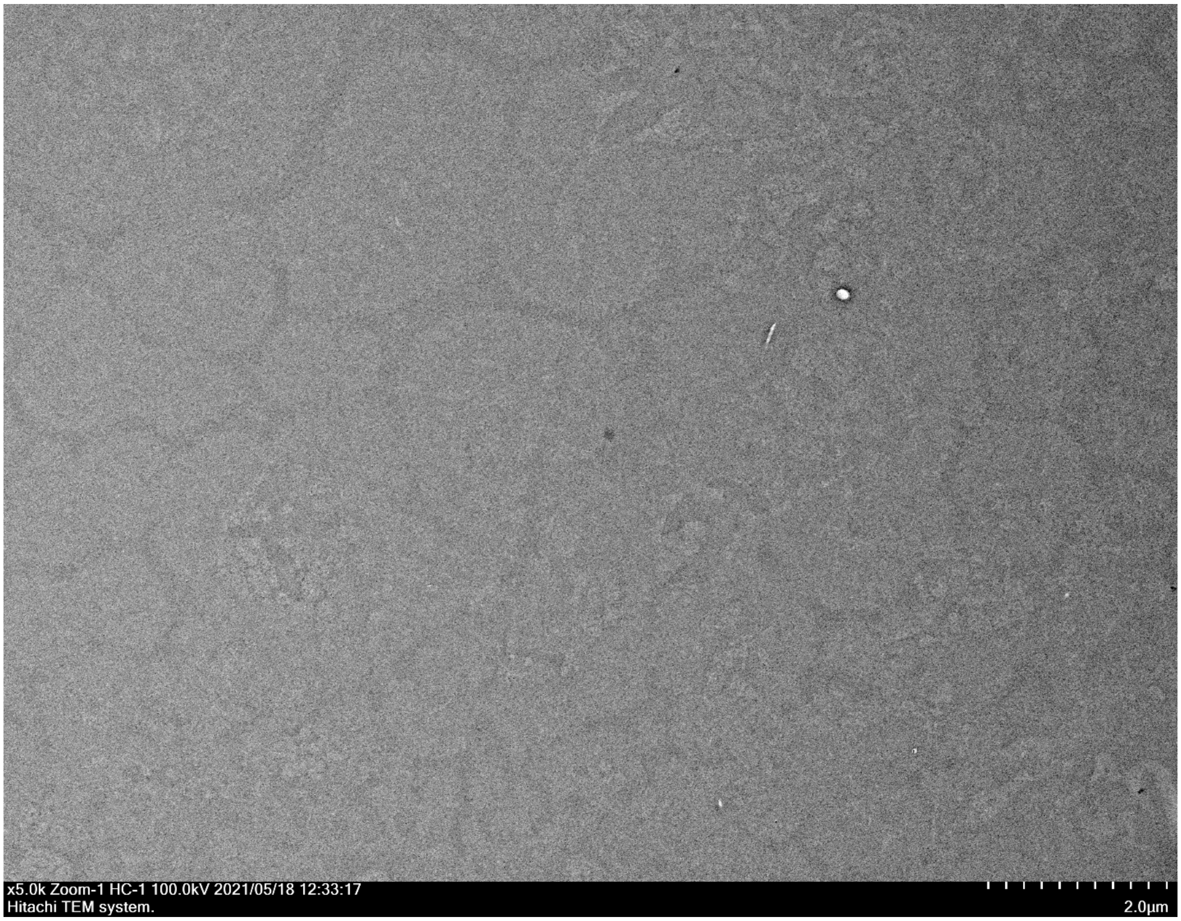

Fig. S70. TEM images of pure **G1-cone-HCl** (5×10^{-5} M). Scale bar 2 μm.

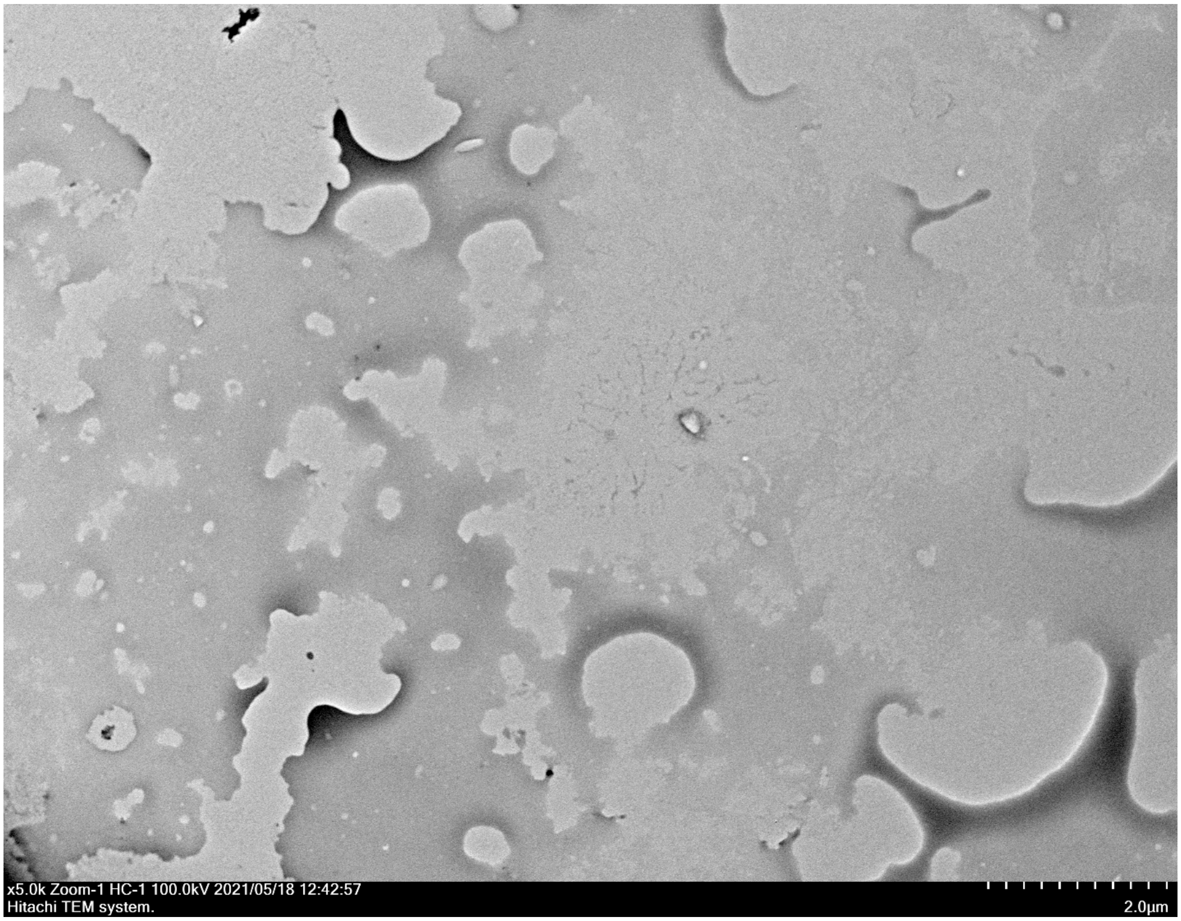

Fig. S71. TEM images of pure DNA (5.565×10^{-5} M base pairs). Scale bar 2 μm.
